# Leveraging Coupled Solvatofluorochromism and Fluorescence Quenching in Nitrophenyl‐Containing Thiazolothiazoles for Efficient Organic Vapor Sensing

**DOI:** 10.1002/advs.202205729

**Published:** 2023-04-26

**Authors:** Andrew R. Brotherton, Abhishek Shibu, Jared C. Meadows, Nickolas A. Sayresmith, Chloe E. Brown, Ana Montoya Ledezma, Thomas A. Schmedake, Michael G. Walter

**Affiliations:** ^1^ Department of Chemistry University of North Carolina at Charlotte Charlotte NC 28223 USA

**Keywords:** asymmetric, chemo‐responsive sensing, fluorophores, organic solvent vapor sensing, push‐pull, solvatofluorochromic, solvent‐dependent Stokes shift, Thiazolo[5,4‐d]thiazole

## Abstract

Solvatofluorochromic molecules provide strikingly high fluorescent outputs to monitor a wide range of biological, environmental, or materials‐related sensing processes. Here, thiazolo[5,4‐d]thiazole (TTz) fluorophores equipped with simple alkylamino and nitrophenyl substituents for solid‐state, high‐performance chemo‐responsive sensing applications are reported. Nitroaromatic substituents are known to strongly quench dye fluorescence, however, the TTz core subtly modulates intramolecular charge transfer (ICT) enabling strong, locally excited‐state fluorescence in non‐polar conditions. In polar media, a planar ICT excited‐state shows near complete quenching, enabling a twisted excited‐state emission to be observed. These unique fluorescent properties (spectral shifts of 0.13 – 0.87 eV and large transition dipole moments Δ*µ* = 20.4 – 21.3 D) are leveraged to develop highly sought‐after chemo‐responsive, organic vapor optical sensors. The sensors are developed by embedding the TTz fluorophores within a poly(styrene‐isoprene‐styrene) block copolymer to form fluorescent dye/polymer composites (*Φ*_F_ = 70 – 97%). The composites respond reversibly to a comprehensive list of organic solvents and show low vapor concentration sensing (e.g., 0.04% solvent saturation vapor pressure of THF – 66 ppm). The composite films can distinguish between solvent vapors with near complete fluorescent quenching observed when exposed to their saturated solvent vapor pressures, making this an extremely promising material for optical chemo‐responsive sensing.

## Graphical abstract

Thiazolo[5,4‐5]thiazole push‐pull fluorophores with nitrophenyl substituents demonstrate unique dual fluorescence properties and strong solvatofluorochromism. The high‐performance dyes are embedded in a block copolymer and utilized in a solid‐state, solvent vapor optical sensing application. TTz/polymer sensors show reversible, low‐concentration vapor sensing, and the ability to distinguish between different organic vapors.

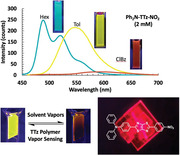

## 1 Introduction

The breadth and variety of optical fluorescence sensing using small‐molecule fluorescent dye sensors are significant.^[^ ^1^, ^2^, ^3^, ^4^ ^]^ Recent examples include fluorescent probes for single‐molecule arrays to detect biological substrates,^[^ ^5^ ^]^ pH‐sensitive fluorescent probes for determining dopamine uptake,^[^ ^6^ ^]^ voltage sensitive dyes to probe cellular membrane potentials,^[^ ^7^, ^8^ ^]^ and temperature probes for therapeutic processes like photothermal therapy.^[^ ^9^ ^]^ In particular, organic solvent vapor sensing using changes in molecular probe fluorescence is an area of intense development.^[^ ^10^, ^11^, ^12^, ^13^, ^14^, ^15^ ^]^ The challenges include enabling high sensitivity to a range of organic solvent vapors, sensor stability/reproducibility, and the ability to distinguish between compounds, often accomplished with cross‐reactive arrays.^[^ ^15^ ^]^ Although a variety of promising dye‐embedded polymer or metal‐organic framework systems have been evaluated,^[^ ^11^, ^14^ ^]^ chemo‐response dyes for these and related applications need further optimization to increase sensing function and versatility.

TTz materials have shown great promise as highly fluorescent, multi‐functional fluorophores.^[^ ^16^, ^17^, ^18^ ^]^ The fused‐thiazolothiazole bicyclic aromatic ring structure is highly planar, thermodynamically stable, and easily synthesized. The rigidity and planarity allow for good *π*‐orbital overlap and rotational inhibition, which limits nonradiative decay pathways and increases fluorescent quantum (QYs) yields.^[^ ^16^ ^]^ Pyridinium‐substituted TTzs have shown chromogenic properties in hydrogel devices demonstrating high‐performance electrochromic, photochromic, and electrofluorochromic capabilities.^[^ ^17^ ^]^ TTzs have also been utilized in metal‐organic frameworks for studying excited‐state energy transfer,^[^ ^19^ ^]^ as a photon upconverter,^[^ ^20^ ^]^ in covalent organic frameworks for hydrogen generation,^[^ ^21^ ^]^ and in polymer photovoltaics.^[^ ^22^, ^23^ ^]^ Asymmetrically substituted, push‐pull TTzs (aTTzs), have shown significant promise for sensing solvent polarity, temperature, pH, and cell membrane potential sensitivities (fractional fluorescence, Δ*F/F* = 9.8%).^[^ ^18^ ^]^ The push‐pull molecular arrangement increases their QYs due to a simultaneous increase in the energy of the singlet state and a decrease in the energy of the triplet state.^[^ ^24^, ^25^ ^]^ In addition, the push‐pull structure creates a strong ICT excited state, and therefore, a large transition dipole moment, resulting in a strong solvatofluorochromic effect whereby emission red‐shifts as the polarity of the surrounding environment increases.^[^ ^26^ ^]^ This is advantageous for improving fluorescence imaging resolution due to an observed large Stokes shift that minimizes the overlap between excitation and emission.

**Figure 1:**
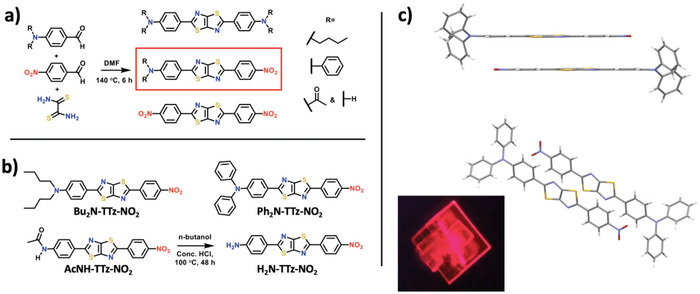
a) Single‐step, synthetic reaction to form asymmetric amino/nitrophenyl TTz fluorophores, b) the four aTTz compounds explored in this work, and c) crystal structure and packing of the Ph_2_N‐TTz‐NO_2_ derivative and single crystal fluorescence microscope image.

To fully utilize the advantages provided by the TTz heterocycle in a fluorescent, optical organic vapor sensing application, we pursued push‐pull aTTzs derivatives with strong intramolecular charge transfer coupled with programmable fluorescence quenching and strong transition dipole moments (Δ*µ*). This enabled greater optical sensing flexibility due to the fluorescence quenching in a polar environment (as opposed to only spectra shifts). We developed a new series of aTTzs synthesized with various donor groups (diphenylamine, dibutylamine, acetamide, and amino), and arguably the strongest organic electron‐withdrawing group, a nitrophenyl substituent (**Figure** **1**). The addition of a nitrophenyl group to a chromophore promotes fluorescence quenching by intersystem crossing (ISC) to a non‐radiative triplet state.^[^ ^27^, ^28^, ^29^ ^]^ However, the TTz bridge enables the nitrophenyl‐containing push‐pull aTTzs to show a selectively high fluorescence emission in non‐polar solvents, and near complete fluorescence quenching in polar solvents due to the presence of the nitrophenyl group. We report the full photophysical properties and solvatofluorochromic behavior with Lippert‐Mataga plots to derive the transition dipole moments (Δ*µ*) of the new compounds. Solvatothermochromic properties are also observed and quantified across a wide range of temperatures. A computational analysis of the aTTz spectra reveals a new phenomenon for amino/nitrophenyl push‐pull fluorescent chromophores whereby long wavelength emission is suppressed revealing a higher energy, twisted intramolecular charge transfer (TICT) state. A polymer film organic vapor sensor was developed using the new aTTz fluorophores co‐dissolved (1 wt.%) in a polystyrene‐block‐polyisoprene‐block‐polystyrene (SIS) copolymer and spin cast onto glass substrates. The sensors detect low concentrations of volatile organic solvents by monitoring both the spectral shift and intensity changes in their fluorescence. Solvents with a variety of polarities can be identified based on the solvatofluorochromic shifts in the solid state and strong quenching when exposed to organic solvent vapors , demonstrating the high‐performance, dual fluorescence sensing capabilities of these materials.

## 2 Results and Discussion

### 2.1 Photophysical Characteristics

Push‐pull dyes with the TTz *π*‐bridge were synthesized using the previously reported mixed substituent reaction conditions with the ratio of donor aldehydes to acceptor aldehydes to dithiooxamide of 3.5:1:1.25 (Figure 1a,b).^[^ ^18^ ^]^ Dye purification was accomplished using column chromatography with 20.9 – 58.5% recovery and overall yields of 9.7 – 25.3% (Supporting Information). The crystal structure of the Ph_2_N‐TTz‐NO_2_ derivative indicates a highly planar phenyl/TTz core, and the molecular packing diagram shows an alternating alignment of neighboring aTTz electron‐donating and withdrawing groups, and bright red emission of the single crystal in the solid state (Figure 1c).

**Figure 2:**
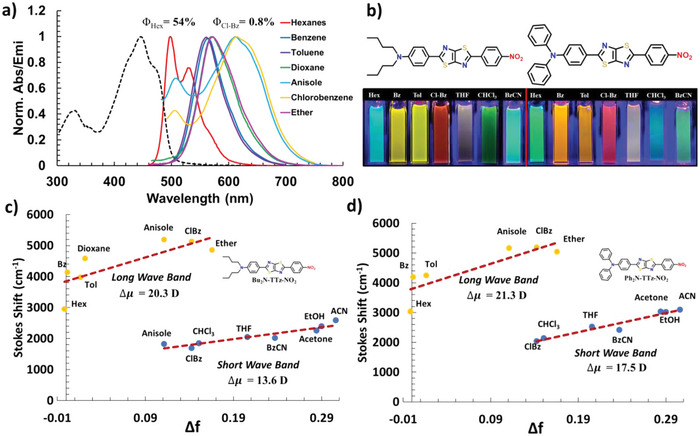
a) Absorbance of Bu_2_N‐TTz‐NO_2_ in hexane (—–) and emission in several solvents. Emissions are obtained with excitation of the absorbance max of the respective solvent. b) Images of the emission of Bu_2_N‐TTz‐NO_2_ and Ph_2_N‐TTz‐NO_2_ in various solvents (400 nm excitation). The images are at various concentrations and light intensities to provide the visible coloration of the excited state emissions. Lippert‐Mataga plots of c) Bu_2_N‐TTz‐NO_2_ and (d) Ph_2_N‐TTz‐NO_2_.

Absorbance, fluorescence, molar absorptivity, fluorescence quantum yield, and fluorescence lifetimes of the aTTzs were obtained in various organic solvents (Table S1, Supporting Information; **Figure** **2**). Molar absorptivity (*ε*) of the four aTTzs ranged from 7000 – 58600 m ^−1^ cm^−1^. Bu_2_N‐TTz‐NO_2_ and Ph_2_N‐TTz‐NO_2_ have an absorbance max (*λ*_abs_) range of 436 – 462 nm in various solvents which are red‐shifted relative to AcNH‐TTz‐NO_2_ and H_2_N‐TTz‐NO_2_. The bathochromic shift between the aTTz derivatives is likely due to an increase in donor strength of the dibutyl and diphenylamino groups resulting in varying electron density across the system. For all solvents except ethanol, there is little variation of *λ*_abs_ indicating minimal solvent effects on the neutral ground state dipole moment. The anomalous behavior of their absorbances in ethanol can be attributed to the presence of hydrogen bonding and solubility differences.^[^ ^26^, ^30^ ^]^ Unlike other push‐pull dyes,^[^ ^31^ ^]^ the *λ*_abs_ has a narrow range (436 – 462 nm) compared to the broad range (487 – 614 nm) of the emission. This demonstrates the charge transfer only in the excited state, making the dyes solvatofluorochromic as opposed to solvatochromic. This has also been confirmed with other aTTzs computationally.^[^ ^32^ ^]^ Cyclic voltammetry measurements were obtained to characterize redox behavior and calculate HOMO/LUMO levels (Figures S51–S59, Tables S3–S5, Supporting Information).

The Ph_2_N‐TTz‐NO_2_ and Bu_2_N‐TTz‐NO_2_ aTTz derivatives exhibit strong solvatofluorochromism (Stoke shifts between 0.13 – 0.65 eV) with high fluorescence quantum yields (QYs) in nonpolar solvents and low QYs in polar solvents (e.g., for Ph_2_N‐TTz‐NO_2_: *Φ*_Hex_ = 69%, *Φ*_BzCN_ = 0.4%) (Figure 2). Decreasing QYs is common for push‐pull fluorophores with strong ICT character in increasingly polar solvents,^[^ ^18^ ^]^ however, the effect here is considerably magnified due to the presence of the nitrophenyl group which favors intersystem crossing (ISC) to a non‐radiative triplet state.^[^ ^33^ ^]^ For instance, diphenylamino/pyridyl aTTz derivative (Ph_2_N‐TTz‐Py) with no nitrophenyl substituents exhibited a QY in CHCl_3_ of *Φ*_F_ = 0.54,^[^ ^18^ ^]^ while the similar Ph_2_N‐TTz‐NO_2_ dye shows a quantum yield of *Φ*_F_ = 0.05. The increase in QY for Ph_2_N‐TTz‐NO_2_ and Bu_2_N‐TTz‐NO_2_ in ethanol is attributed to hydrogen bonding in the excited state.^[^ ^30^ ^]^ Interestingly, AcNH‐TTz‐NO_2_ has a QY between that of the symmetric TTzs, (AcNH)_2_TTz (*Φ* ${}_{{\mathrm{CHCl}}_{3}}$ = 0.37) and (NO_2_)_2_TTz (*Φ* ${}_{{\mathrm{CHCl}}_{3}}$ < 0.01). H_2_N‐TTz‐NO_2_ has a broad range of emission with onsets from 420 nm to 650 nm (Figure S26, Supporting Information) demonstrating the presence of a strong ICT state with low QYs similar to fluorophores containing alkyne or triphenyl *π*‐bridges.^[^ ^34^, ^35^ ^]^ Fluorescence lifetimes (*τ*_F_) in various solvents show an increase when increasing the polarity from hexane to toluene (Bu_2_N‐TTz‐NO_2_, *τ*_F_ = 2.18 to 2.74 ns and Ph_2_N‐TTz‐NO_2_, *τ*_F_ = 2.26 to 2.87 ns). This is representative behavior for increasing ICT character in the excited state,^[^ ^26^ ^]^ however, beyond this solvent polarity, the *τ*_F_ becomes shorter as the ICT state is almost completely quenched by the nitrophenyl group. The non‐radiative rate (Bu_2_N‐TTz‐NO_2_, *k* **_nr_**_CHCl3_ = 8.50 × 10^8^ s^−1^) is therefore faster than the radiative rate (*k* **_r_**_CHCl3_ = 2.72×10^7^ s^−1^) in more polar solvents.

The highly planar aTTzs featured in this work (Figure 1c) favor electron density on the nitrophenyl groups in the ICT excited state, resulting in a strong, push‐pull solvatofluorochromic effect. For instance, the fluorescence of Bu_2_N‐TTz‐NO_2_ is red‐shifted 115 nm (*λ*_emi_) (Bu_2_N‐TTz‐NO_2_, *λ*_emi hex_ = 499 to *λ*_emi ClBz_ 614 nm). To quantify this, we evaluated the fluorescence emission intensities in a variety of solvents with a range of polarities and used their Stokes shifts to evaluate their excited‐state dipole behavior (Figure 2). An important feature observable in the emission spectra for several polar solvents (e.g., chlorobenzene, anisole) are two bands, a short wavelength band (SWB ≈500 nm) and a long wavelength band (LWB ≈625 nm) (Figure 2a). Previous studies on similar amino‐nitro push‐pull dyes associated the multiple band, dual fluorescence to a twisted ICT (TICT) state and a locally excited (LE) state.^[^ ^24^, ^36^, ^37^, ^38^ ^]^ Compared to initial studies of aTTz compounds,^[^ ^18^ ^]^ the SWB is considerably more visible due to strong quenching of the ICT (LWB) state by the nitrophenyl group in polar media. Only the LE (SWB) is observable in strongly polar solvents (CHCl_3_ and benzonitrile – Figures S18 and S22, Supporting Information), which gives the appearance of a blue‐shifted solvatofluorochromism (Figure 2b). To confirm the presence of the ICT state, strong acid (TFA) was used to protonate the aTTz derivative, limiting the shift of electron density and increasing polarity in the excited state (Figure S19, Supporting Information).^[^ ^18^, ^39^ ^]^ Due to the dual solvatofluorochromic effect in Bu_2_N‐TTz‐NO_2_ and Ph_2_N‐TTz‐NO_2,_ the excited‐state dipole moments (**Table** **1**) were calculated separately for the SWB and LWB bands using the Lippert‐Mataga (LM) equation (Equation 1):

(1) $$\begin{array}{c} \nu_{a}-\nu_{f}=\frac{2{\left(\mu^{*}-\mu \right)}^{2}}{4\pi \varepsilon_{0}hca^{3}}\Delta f+const.;\Delta f=\left(\frac{\varepsilon -1}{2\varepsilon +1}-\frac{\eta^{2}-1}{2\eta^{2}+1}\right) \end{array}$$

where *ν*_*a*_ and *ν*_*f*_ are the absorption and emission peaks in cm^−1^, µ* and *µ* are the excited state and ground state dipoles, *ε*_0_ is the vacuum permittivity, *h* is Planck's constant, *c* is the speed of light, *a* is the Onsager cavity radius, Δ*f* is the orientation polarizability, *ε* is the relative permittivity, and *η* is the refractive index. The ground state dipole and Onsager cavity radius were calculated using Gaussian software. The 4*πε*_0_ constant comes from the reaction field factor.^[^ ^40^ ^]^

The transition state dipole moments (Δ*µ* of the LWB) of the TTz compounds (Δ*µ* = 20.4 – 21.3 D – Table 1) are larger than previous aTTz materials,^[^ ^18^ ^]^ and comparable to some of the largest push‐pull dyes reported including fluorene‐based Prodan derivatives (Δ*µ* = 14 D),^[^ ^41^ ^]^ ladder‐type dyes (Δ*µ* = 19 D),^[^ ^42^ ^]^ flavonoid dyes (Δ*µ* = 15.4 D),^[^ ^43^ ^]^ and aryl‐hydroxychromones (Δ*µ* = 15 D).^[^ ^41^, ^44^ ^]^ The aTTz dyes reported here also have a wide spectral Stokes shift range between 0.13 – 0.87 eV (1050 – 7020 cm^−1^). Arylaminothiazole dyes have also shown strong Stokes shifts in CHCl_3_ (≈5900 cm^−1^), but smaller changes in spectral shift versus solvent polarity result in a lowered Δ*µ* = 11 D.^[^ ^45^ ^]^ Importantly, the aTTz materials include a strongly quenching nitrophenyl substituent resulting in an observable LE (SWB) which is unique among these push‐pull dyes. Therefore, we have quantified the Δ*µ* separately for both LWB and SWB excited states associated with each aTTz dye reported. This analysis was possible due to the excited‐state characteristics of the TTz core, and the strongly electron‐withdrawing nitrophenyl group enabling large ICT excited states to be observed for both the LWB and SWB transitions. The LWB transition (Δ*µ* = 20–21 D) is considerably more polar than the SWB (Δ*µ* = 10–17 D) excited state, however, LM fit analyses of the LWB show a slightly lower degree of linearity (Figure 2c,d). In addition, the LM plots do not account for solute‐solvent specific interactions, nor the polarizability of solute, which likely impact the LWB/ ICT photophysical dynamics.^[^ ^26^ ^]^

**Table 1:** Ground and Excited State Dipole Moments

Compound	Onsager Cavity Radius *a* [Å]^a^	Ground State Dipole *µ* [D]^a^	Excited State Dipole µ* [D]^b^	Change in Dipole Δ*µ* [D]^b^
Bu_2_N‐TTz‐NO_2_ (LWB)	7.88	12.3	32.7	20.4
Bu_2_N‐TTz‐NO_2_ (SWB)	7.88	12.3	25.9	13.6
Ph_2_N‐TTz‐NO_2_ (LWB)	7.88	9.26	30.6	21.3
Ph_2_N‐TTz‐NO_2_ (SWB)	7.88	9.26	26.9	17.7
AcNH‐TTz‐ NO_2_ (LWB)	7.87	9.23	29.8	20.6
AcNH‐TTz‐NO_2_ (SWB)	7.87	9.23	19.7	10.4

^a^ Calculated using DFT PBE1PBE/6‐311G+(d,p) with tight SCF, fine grid integral, and volume keyword

^b^ Semi‐empirically calculated using the Lippert‐Mataga Equation

^a,b^ The small change in absorbance results in a small change in *µ* when the aTTzs are dissolved in various solvents. Therefore, the calculated µ is sufficient for Δ*µ* and *µ* ^*^.

**Figure 3:**
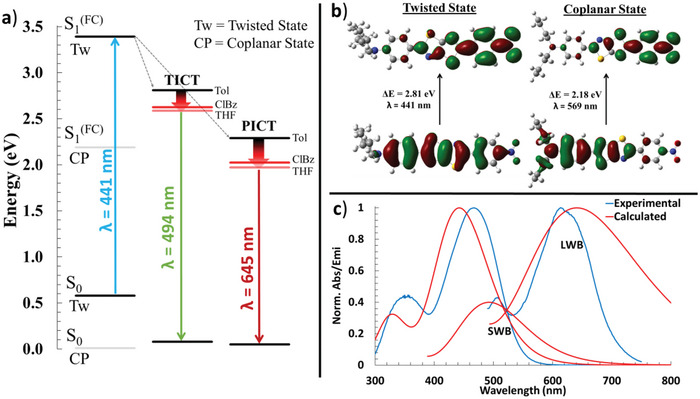
a) Modified relative energy Jablonski diagram showing the ground state optimized coplanar and twisted state, the Frank‐Codon excited states (in THF), the excited state minima's of the twisted (90°) and coplanar states in various solvents, and their ground state upon emission (in THF). b) The HOMO and excited state (FC) MOs of the twisted and coplanar states. c) Experimental and calculated spectra of Bu_2_N‐TTzNO_2_ in ClBz.

Further insight into the large shift in excited‐state dipole moments was obtained computationally. Excited states were modeled in Gaussian using a hybrid functional PBE0 (PBE1PBE) with a 6–311+G(d,p) basis set and integral equation formalism model (IEFPCM) for solvation.^[^ ^36^, ^46^, ^47^ ^]^ Initial optimization of the TTz derivatives in a vacuum demonstrates the push‐pull nature of the molecules with HOMOs residing on the aminophenyl donating groups, and LUMOs on the nitrophenyl groups (Figures S41–S43, Supporting Information). To observe both SWB and LWB bands, Bu_2_N‐TTz‐NO_2_ was modeled in chlorobenzene (ClBz), however, time‐dependent DFT (TDDFT) calculations did not properly reflect the experimental absorbance spectra (Figure S48, Supporting Information). The potential for both a planarized and a twisted intramolecular charge transfer state (PLATICT) was explored due to the possibility of a twisted (amino‐carbon phenyl) bond in the excited state.^[^ ^36^, ^37^ ^]^ Rotating and fixing the TTz amino‐phenyl dihedral bond of Bu_2_N‐TTz‐NO_2_ to 90° (Figure S44, Supporting Information), and modeling the absorbance spectra provided a good fit of the experimental absorbance and emission spectra (**Figure** **3**c). This is strong evidence for the TTz dyes to be in a twisted ground state. The Frank‐Codon excited states were found, and geometrically optimized to determine the excited state minima (ESM), and an emission spectrum was calculated. A twisted ESM was optimized by also holding in place the dihedral angles. The coplanar ESM most closely fits the LWB emission while a twisted ESM fits the emission of the SWB (Figure 3c; Figures S49 and S50, Supporting Information). Therefore, the LWB is associated with a planar intramolecular charge transfer (PICT) state, while the SWB is associated with a twisted ICT (TICT) state. Bu_2_N‐TTz‐NO_2_ was also modeled in toluene, where only the PICT state is observed, and in THF, where only the TICT state is observed (Figures S49 and S50, Supporting Information). A calculated bathochromic shift in emission is also observed, following the experimental solvatofluorochromic effects (Figure 3a).

### 2.2 High‐Performance Optical Sensing Applications

**Figure 4:**
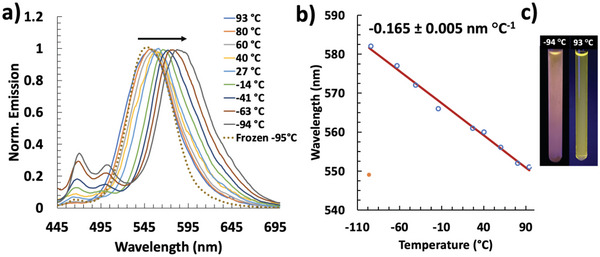
a) Normalized emission intensity spectra of Bu_2_N‐TTz‐NO_2_ and b) temperature‐wavelength correlation profile of Bu_2_N‐TTz‐NO_2_ in Toluene when *T* > −96 °C (blue) and *T* ≤ −96°C (orange/red).

Having obtained insights into the excited state characteristics of these new TTz dye systems, we studied their strong solvatofluorochromism in a Bu_2_N‐TTz‐NO_2_ thermofluorochromic application and exploited the tunable nitrophenyl fluorescence quenching properties in a solid‐state organic vapor polymer sensing platform. Thermochromism is complementary to solvatofluorochromism where the solvent polarity and therefore emission of a push‐pull dye is dependent on temperature.^[^ ^18^, ^48^ ^]^ Bu_2_N‐TTz‐NO_2_ was chosen because of a large Stokes shift, good solubility, and representative photophysical characteristics. The normalized fluorescence emission of Bu_2_N‐TTz‐NO_2_ solutions in toluene were monitored from −94 to 94 °C (**Figure** **4**). Toluene was chosen for its wide liquid temperature window (−94.9 – 110 °C) and ability to form a glass upon freezing.^[^ ^49^ ^]^ Bu_2_N‐TTz‐NO_2_ also has a large QY in toluene and a single LWB (ICT) peak, which simplified monitoring changes in emission. The low‐temperature studies were achieved using various liquid N_2_ cooling baths (Supporting Information).^[^ ^18^ ^]^

The emission of Bu_2_N‐TTz‐NO_2_ redshifts as the temperature decreases from 551 nm at 94 °C to 582 nm at −94 °C (Figure 4a–c), demonstrating a high linear temperature sensitivity (−0.17 nm °C^−1^), and allowing this platform to be used for a variety of temperature‐sensing applications.^[^ ^50^, ^51^, ^52^ ^]^ Higher temperatures prevent alignment of solvent dipoles leading to the observed blue shift in the emission spectra of the aTTz dyes.^[^ ^18^, ^53^ ^]^ Unique to the nitrophenyl aTTz dyes, is the appearance and strengthening of the SWB (TICT) emission (450 – 500 nm) as the temperature decreased (−14 to −94 °C) (Figure 4a). Upon freezing the solvent (< −94 °C), the hypsochromic emission shift (to ≈550 nm) observed is attributed to the complete inhibition of solvent relaxation. In highly polar solvents like Me‐THF, less sensitivity to temperature was observed (−0.06 nm °C^−1^) (Figures S38 and S39, Supporting Information – tracking SWB *λ*_emi_). Interestingly, a strong excimer emission is also observed as a broad peak at 620 nm , and upon cooling or dilution, the intensity of the excimer emission is reduced (Figure S37, S38, Supporting Information).

**Figure 5:**
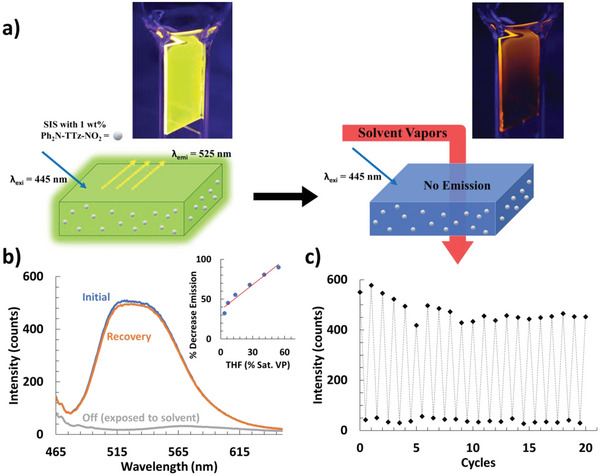
a) Fluorescent film on glass of TTz (1 wt.% Ph_2_N‐TTz‐NO_2_) embedded in SIS polymer, and exposure to organic solvent vapors to quench the fluorescence (inset 1 cm x 2.5 cm composite film on glass slide, 390 nm excitation). b) Emission spectrum of a single exposure of Ph_2_N‐TTz‐NO_2_ THF (at the saturated solvent vapor pressure) with an inset of detecting 7 – 67% of saturated solvent vapor pressure (Standard deviation percent fluorescence decrease, 45 ± 0.05% at 6.7% saturated THF vapor). c) Cycling plot of the max emission during sequential exposure (*λ*_emi_ = 520 nm and *λ*_exi_ = 445 nm).

We investigated the solid‐state sensing performance of TTz nitro‐containing asymmetric dyes by embedding the Ph_2_N‐TTz‐NO_2_ derivative in a block copolymer for organic solvent vapor sensing. Fluorescent dyes have shown sensitivity to organic vapors by relying on aggregation‐induced emission through polymer swelling,^[^ ^10^ ^]^ fluorescence changes of molecular solids on filter paper,^[^ ^54^, ^55^ ^]^ in nanomaterials,^[^ ^56^ ^]^ or in printed arrays.^[^ ^57^, ^58^, ^59^ ^]^ Fluorescent organic dyes embedded in a variety of polymeric materials have also shown promising platforms as organic dye‐polymer phosphor layers.^[^ ^60^, ^61^ ^]^ The advantages of using Ph_2_N‐TTz‐NO_2_ in a polymer vapor sensor are the dual properties of solvatofluorochromism due to the strong excited‐state dipole change, and the fluorescence quenching via ICS induced by the nitrophenyl group under exposure to polar solvent vapors. We compared the optical sensing performance and properties of these composites between two TTz dyes systems; the Ph_2_N‐TTz‐NO_2_ reported here and a previously reported TTz dye (Ph_2_N‐TTz‐Py) with no nitrophenyl group.^[^ ^18^ ^]^ Both dyes show very similar crystal structures with a highly planar phenyl/TTz core (Figure 1c). We developed a simple dye‐polymer composite optical fluorescence sensor platform by embedding the push‐pull TTz dyes in styrene‐isoprene‐styrene (SIS) block copolymers (**Figure** **5**a). The SIS polymer material is a low‐cost, commercially available thermoplastic elastomer that shows excellent processability and can be sprayed on or hot‐melted to form adhesive layers. Optical vapor sensing platforms were developed by dissolving ≈1 wt.% aTTz dyes and SIS block copolymer into toluene and spin‐casting onto a glass slide to produce a highly fluorescent thin polymer film (Figure 5a). The fluorescence of the dye‐polymer composite films shows emission spectra that indicate the polymer environment is similar to the solvatofluorochromic dyes dissolved in toluene (Ph_2_N‐TTz‐Py *λ*_emi_ ≈ 490 nm and Ph_2_N‐TTz‐NO_2_ *λ*_emi_ ≈ 520 nm). The embedded dye films also show very high fluorescent quantum yields exceeding those observed in non‐polar media (Ph_2_N‐TTz‐Py *Φ*_F_ = 97% and Ph_2_N‐TTz‐NO_2_ *Φ*_F_ = 70%) and surprisingly long‐lived fluorescent lifetimes (Ph_2_N‐TTz‐Py *τ*_f_ = 3.18 ns and Ph_2_N‐TTz‐ NO_2_ *τ*_f_ = 2.64 ns) indicating well‐dispersed, polymer‐embedded TTz dyes.

Organic vapor sensitivity was evaluated by exposing both TTz dye/polymer composite films to a variety of organic solvent vapors in a closed spectrofluorometric cell (Figure 5; Tables S6 and S7, Figures S60–S84, Supporting Information). The emission of the Ph_2_N‐TTz‐NO_2_ polymer composite decreased significantly when exposed to increasingly more polar solvent vapors with a 12% decrease when exposed to toluene and a near 100% decrease when exposed to THF or chloroform at their saturated solvent vapor pressures (Figure 5b, Figure S60, Supporting Information). There was also a visible red shift from the polymer's emission at 520 nm to when exposed to polar solvents (*λ*_emi tol_ = 544 nm and *λ*_emi THF_ = 570 nm) demonstrating how the solvatofluorochromic effect enables the films to distinguish between toluene, ether, THF and DCM solvent vapors (Figure S74, Supporting Information). Additionally, there is a red spectral shift due to solvent vapor concentration, likely due to the varying degrees of polarity in the mixed non‐polar (SIS polymer) and polar (solvent vapor) environments (Figure S75, Supporting Information). The quenching effect of the nitrophenyl allows a distinct contrast of emission to be easily detected (Figure 5a). The fluorescence response speed was less than 1 s when the films were exposed to volatile solvents like DCM and THF, while thicker drop‐cast films had slower fluorescence changes, (20–30 s). To further evaluate the durability of the sensor, films were exposed to 20 consecutive cycles of THF and DCM vapors (Figure 5c; Figures S70–S73, Supporting Information). Minimal fluorescence fluctuations and stable cycling were obtained after 3–4 on/off cycles. Organic solvent vapors of THF were detected by the Ph_2_N‐TTz‐NO_2_ dye/polymer composite film at low concentrations of saturated vapor pressure (0.04%, 66 ppm) with excellent reproducibility when sensing at a range of organic vapor concentrations (Figure 5b). Previously reported optical organic vapor sensors using polymeric swelling induced variation and fluorescence indicated a methanol or acetone solvent vapor detection limit of 100 ppm,^[^ ^10^, ^11^ ^]^ while a dye‐incorporate, highly porous metal–organic framework (MOF) was reported to detect acetone with a detection limit of 60 ppm.^[^ ^14^ ^]^ Interestingly, unlike the Ph_2_N‐TTz‐NO_2_, the Ph_2_N‐TTz‐Py dye retains good fluorescence QY in solvents with increasing polarity such as DCM, ethyl acetate, and CHCl_3_.^[^ ^18^ ^]^ Therefore, polymer sensing using a Ph_2_N‐TTz‐Py dye/polymer composite indicated strong solvatochromic shifts while maintaining strong fluorescence emission intensity when exposed to vapors of solvents with a variety of polarities (Tables S6– S8, Figures S78–S83, Supporting Information).

**Figure 6:**
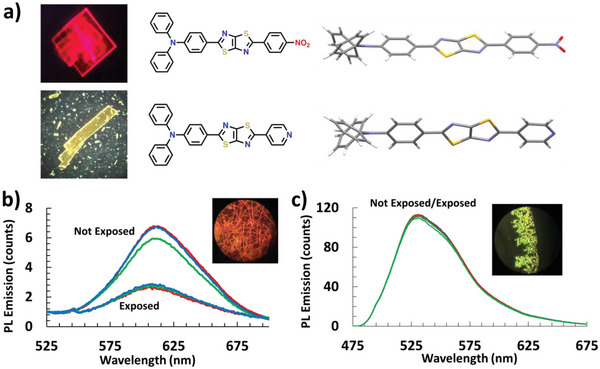
a) Single crystal fluorescence microscope images of Ph_2_N‐TTz‐NO_2_ and Ph_2_N‐TTz‐Py derivatives, crystal structure and b) thin compact crystalline film and emission spectrum of Ph_2_N‐TTz‐NO_2_ exposed to DCM vapors and c) thin compact crystalline film and emission spectrum of Ph_2_N‐TTz‐PY exposed to DCM vapors.

Thin film fluorescence and solvent vapor sensing were also evaluated using compact crystalline films, that were prepared by drop‐casting DCM solutions onto glass substrates (**Figure** **6**a–c). Although the two TTz derivatives show nearly identical crystal structures and packing, the fluorescence of the Ph_2_N‐TTz‐NO_2_ derivative is significantly red‐shifted (*λ*_NO₂_ = 613 nm and *λ*_py_ = 531 nm – Figure 6a). In addition, when the crystalline films of the Ph_2_N‐TTz‐Py derivative are exposed to solvent vapors, there is little or no solvatofluorochromic response, whereas the Ph_2_N‐TTz‐NO_2_ derivative shows a significant reduction in the PL emission (Figure 6b,c).

## 3 Conclusion

A new family of asymmetric, thiazolothiazole (aTTz) amino‐nitro push‐pull dyes allowed for high sensitivity in a variety of applications, including solution‐state sensing, temperature sensing, and chem‐responsive, solid‐state organic solvent vapor sensing. Transition dipole moments were determined from Lippert‐Mataga plots and photophysical measurements indicate a dual fluorescent, secondary excited state modeled using computational studies to elucidate the ICT nature of the dual fluorescence. The transition dipole moments are among the highest ever reported for small‐molecule fluorescent probes. The fluorescence quenching of the nitrophenyl group significantly increases the environmental sensitivity of the aTTzs. When embedded in a porous polymer, aTTzs can be used for sensing a variety of solvent vapors with a range of vapor pressures and functional groups (ketones, amines, alcohols, and aromatic compounds). The optical sensing thin films demonstrated good cyclability and low detection limits. The fundamental spectroscopic insights into the unique properties of nitrophenyl‐containing push‐pull aTTz dye compounds will enable further improvements in molecular probe sensitivity.

## Conflict of Interest

The authors declare no conflict of interest.

## Supporting information

Supporting Information

## Data Availability

The data that support the findings of this study are available in the supplementary material of this article.
