# Supplementary material for: Leveraging Coupled Solvatofluorochromism and Fluorescence Quenching in Nitrophenyl‐Containing Thiazolothiazoles for Efficient Organic Vapor Sensing
Source: Adv Sci (Weinh). 2023 Apr 26;10(18):2205729. doi: 10.1002/advs.202205729 (PMC10288239; doi:10.1002/advs.202205729)
Supplement: Supplementary file 1 — Supporting Information [file ADVS-10-2205729-s001.pdf]

ADVANCED SCIENCE
Open Access

Supporting Information

for *Adv. Sci.*, DOI 10.1002/advs.202205729

Leveraging Coupled Solvatofluorochromism and Fluorescence Quenching in Nitrophenyl-Containing Thiazolothiazoles for Efficient Organic Vapor Sensing

*Andrew R. Brotherton, Abhishek Shibu, Jared C. Meadows, Nickolas A. Sayresmith, Chloe E. Brown, Ana Montoya Ledezma, Thomas A. Schmedake and Michael G. Walter**

Supporting Information

Leveraging Coupled Solvatofluorochromism and Fluorescence Quenching in Nitrophenyl-Containing Thiazolothiazoles for Efficient Organic Vapor Sensing

*Andrew R. Brotherton, Abhishek Shibu, Jared C. Meadows, Nickolas A. Sayresmith, Chloe E. Brown, Ana Montoya Ledezma, Thomas A. Schmedake, and Michael G. Walter**

Table of Contents

Section 1: Materials and Methods 2

Section 1.1: Materials and Instrumentation 2

Section 1.2: Cyclic Voltammetry 3

Section 1.3: Solvent Vapor Polymer Sensing Studies Experimental 3

Section 1.4: Synthesis 4

Section 1.4.1: 2-(N,N-dibutyl-4-aminophenyl)-5-(4-nitrophenyl) thiazolo[5,4-d]thiazole (Bu_2N-TTz-NO_2) 4

Section 1.4.2: 2-(N,N-diphenyl-4-aminophenyl)-5-(4-nitrophenyl) thiazolo[5,4-d]thiazole (Ph_2N-TTz-NO_2) 5

Section 1.4.3: 2-(4-acetoaminophenyl)-5-(4-nitrophenyl) thiazolo[5,4-d]thiazole (AcHN-TTz-NO_2) 5

Section 1.4.4: 2-(4-aminophenyl)-5-(4-nitrophenyl) thiazolo[5,4-d]thiazole (H_2N-TTz-NO_2) 6

Section 1.4.5: Bis-(4-acetamidophenyl)-thiazolo[5,4-d]thiazole ($(AcNH)_2$TTz) 6

Section 1.4.6: Bis-(4-aminophenyl)-thiazolo[5,4-d]thiazole ($(H_2N)_2$TTz) 7

Section 2: Spectra Characterization 7

Section 2.1: NMR 7

Section 2.2: MALDI-MS 12

Section 2.3: Optical Properties of amino/nitrophenyl aTTz Dyes in Various Solvents 15

Section 3: Solvent and Temperature Sensitivity 16

Section 3.1: Absorbance and Emission 16

Section 3.2: Fluorescence Lifetimes 22
Section 3.3: Quantum Yield and Molar Absorptivity 26
Section 3.4: Lippert-Mataga Plots 27
Section 3.5: Temperature Sensitivity 29
Section 3.6: Polarizability 30
Section 4: Computational Studies 31
Section 5: Cyclic Voltammetry Figures and Tables 36
Section 5.2: Diffusion Calculations 39
Section 6: Organic Vapor Polymer Sensor 41
Section 6.1: Solvent Vapor Sensing with Ph_2N-TTz-NO_2/SIS 41
Section 6.2: Solvent Vapor Sensing with Ph_2N-TTz-Py/SIS 48
Section 7: Solid-State Characterization 52
Section 7.1: Lifetimes 52
Section 7.2: Absolute Solid State Fluorescence Quantum Yield 53
Section 7.3: Compact aTTz Film Solvent Vaper Sensing 53
Section 8: Crystal Structure Data and Refinement 54

Section 1: Materials and Methods

Section 1.1: Materials and Instrumentation

4-nitrobenzaldehyde, 4-(dibutylamino)benzaldehyde, 4-(diphenylamino)benzaldehyde, 4-acetamidobenzladehyde, dithiooxamide, tetrabutylammonium hexafluorophosphate (TBAH) and all solvents used for spectroscopic measurements were purchased from Sigma-Aldrich and used without further purification. ^{1}H and ^{13}C NMR measurements were obtained with either a JEOL 300 MHz NMR or a JEOL 500 MHz NMR. High resolution mass spectra were obtained using a Voyager Matrix Assisted Laser Desorption/Ionization Time-of-Flight (MALDI-TOF) mass spectrometer, using Anthracene-1,8,9-triol as a matrix. Solution-state UV-Vis spectra were collected on a Cary 300 UV-Vis spectrophotometer. Time-resolved fluorescence lifetime measurements (time-correlated single-photon counting - TCSPC) were taken on a Jobin Yvon-Spex Fluorolog equipped with a 389 nm diode laser for time-resolved PL decay measurements. All decays were calculated with a $\mathcal{X}^2 \leq 1.2$. Igor Pro 6.3 software was used to fit

PL(*t*) decay data to single/multiple exponential decays. Quantum yields were calculated using perylene orange, perylene, and 9,10-diphenylanthracene as a references (quantum yield [Φ_F] in chloroform = 0.99 ± 0.05, [Φ_F] in cyclohexane = 0.94, and [Φ_F] in cyclohexane = 0.90, respectively).[1] Temperature studies were conducted using a 10 µM Bu_2N-TTz-NO_2 Toluene solution placed inside of a Norrell 502 NMR tube and sequentially submerged in the following liquid N_2 baths: octanol (-16 ^oC), acetonitrile (-41 ^oC), acetone (-94 ^oC), chloroform (-63 ^oC) and liquid N_2 itself (-196 ^oC). Unless otherwise noted, all experiments were conducted at room temperature. Due to the condensation of atmospheric moisture during low-temperature testing, only the normalized emission intensities are reported.

Section 1.2: Cyclic Voltammetry

A Gamry Reference 600 potentiostat was used for cyclic voltammetry to obtain the redox potentials and reversibility of the a-TTzs. All solutions were purged with argon. A platinum working, platinum foil counter, and Ag/$AgNO_3$ reference electrodes were used with ferrocene (fc) as an internal standard. An a-TTz was dissolved into a 0.1 M TBAH 4 mL solution of DCM until an adequate signal was observed. Scan rates of 50, 100, 150, 200, 250, 300, 400, 500 mV s^{-1} were used, and Fc was used at the end. The DCM was removed, and the solid was dissolved in toluene to determine the concentration using the molar absorptivity. The diffusion coefficient was determined using the Randles-Sevcik Equation.

Section 1.3: Solvent Vapor Polymer Sensing Studies Experimental

Organic vapor sensing studies (using a fixed volume) were conducted using 0.45 mM Ph_2N-TTz-NO_2 and Ph_2N-TTz-Py in toluene for SIS. A density of 100 mg/mL of SIS (20% styrene by wt.) was used and 100 µL of each were spin cast (500 rpm) onto glass microscope slides. Emission was measured in a glass cuvette chamber (**Figure S83**) before, during, and after exposure. 100 µL of each solvent was used and the ppm was calculated using the ideal gas law

from the vapor pressure of the solvent (at 25 °C) and the volume of the chamber (23.3 mL). The spin cast polymer was cycled on and off by repeat exposures of the solvent to the slide. The chamber was dried upon each cycle with nitrogen gas. Total exposure to solvent each cycle was 1.5 min with 3 min of aerated drying time in between each measurement. For emission scans, Ph_2N-TTz-NO_2 was excited at 445 nm and Ph_2N-TTz-Py was excited at 400 nm. A single polymer/dye composite prepped slide was used for an entire iteration where the slide was exposed to increasing concentrations of solvents with the ppm calculated from the vapor pressure (**Figures S75 – S78 and S82 – S83)**. A lower detection limit for THF was determined using a larger volume flask (4.5 L) and longer exposure time (5 min) to THF solvent vapors.[2, 3]

Section 1.4: Synthesis

Section 1.4.1: 2-(N,N-dibutyl-4-aminophenyl)-5-(4-nitrophenyl) thiazolo[5,4-d]thiazole (Bu_2N-TTz-NO_2)

Dithiooxamide (0.2883 g, 2.399 mmol), 4-(diphenylamino)benzaldehyde (1.9975 g, 8.566 mmol), and 4-nitrobenzaldehyde (0.3079 g, 2.037 mmol) were mixed in 18 mL of DMF and heated to 140 °C for 6 h in an aerated environment. The reaction was cooled to room temperature and left to sit in a fridge for 24 h. The solution was vacuum filtered, rinsed with water, and dried under vacuum to give a brownish solid (0.3190 g). Using an eluent of hexanes: ethyl acetate, 6:1, 13.8 mg of the crude product was purified by silica gel column chromatography (Silica Flash M60). The eluent was removed under vacuum, yielding a brown solid (4.0 mg, 29.0% recovery yield) giving an overall 9.7% yield. **^{1}H NMR** (300 MHz, d-$CDCl_3$, δ): 8.32 (d, *J* = 8.8 Hz, 2H), 8.13 (d, *J* = 8.8 Hz, 2H), 7.83 (d, *J* = 8.8 Hz, 2H), 6.67 (d, *J* = 8.8 Hz, 2H), 3.35 (t, *J* = 7.9 Hz, 4H), 1.61 (p, *J* = 7.6 Hz, 4H), 1.38 (h, *J* = 7.6 Hz), 0.98 (t, *J* = 7.6 Hz, 6H). **^{13}C NMR** (126 MHz, $CDCl_3$, δ): 172.51, 163.13, 152.61, 150.52, 150.23, 148.27, 139.89, 128.30, 126.56, 124.53, 120.52, 111.38, 50.89, 29.47, 20.39, 14.06. **UV-Vis λ_{max} ($CHCl_3$, $M^{-1}cm^{-1}$):** 462 nm (ε = 19,900). **MALDI-TOF-MS:** m/z calculated for $C_{24}H_{26}N_4O_2S_2$ 466.618, found 466.601.

Section 1.4.2: 2-(N,N-diphenyl-4-aminophenyl)-5-(4-nitrophenyl) thiazolo[5,4-d]thiazole (Ph_2N-TTz-NO_2)

4-nitrophenylbenzaldehyde (0.3079 g, 2.039 mmol), dithiooxamide (0.3028 g, 2.523 mmol), and 4-(diphenylamino)benzaldehyde (2.3984 g, 8.785 mmol) were mixed in 18 mL of DMF for 6 h at 140 °C in an aerated environment. The reaction was cooled to RT and sat overnight, a red-orange precipitate was collected via vacuum filtration and rinsed with water (0.3696 g). Using an eluent of DCM: hexanes, 2:1, 15.9 mg of the crude product was purified by silica gel column chromatography (Silica Flash M60). The eluent was removed under vacuum, yielding a red solid (9.3 mg, 58.5% recovery yield) giving an overall 20.9% yield. **^{1}H NMR** (500 MHz, d-$CDCl_3$, δ): 8.33 (d, *J* = 8.9 Hz, 2H), 8.15 (d, *J* = 8.9 Hz, 2H), 7.83 (d, *J* = 8.9 Hz, 2H), 7.32 (t, *J* = 8.9 Hz, 4H), 7.17 (d, *J* = 8.9 Hz, 4H), 7.13 (t, *J* = 8.9 Hz, 2H), 7.08 (d, *J* = 8.9 Hz, 2H). **^{13}C NMR** (126 MHz, $CDCl_3$, δ):171.12, 164.45, 152.45, 151.06, 150.70, 148.55, 146.77, 139.62, 129.68, 127.70, 126.80, 125.70, 124.56, 124.43, 124.09, 121.50. **UV-Vis λ_{max} ($CHCl_3$, $M^{-1}cm^{-1}$):** 446 nm (ε = 11,400). **MALDI-TOF-MS:** m/z calculated for $C_{28}H_{18}N_4O_2S_2$ 506.598, found 506.570.

Section 1.4.3: 2-(4-acetoaminophenyl)-5-(4-nitrophenyl) thiazolo[5,4-d]thiazole (AcHN-TTz-NO_2)

4-nitrophenylbenzaldehyde (0.600 g, 3.97 mmol), 4-acetamidobenzaldehyde (0.648 g, 3.97 mmol), and dithiooxamide (0.477 g, 3.97 mmol), were mixed in 70 mL anhydrous DMF for 6 h at 140 °C. The reaction mixture was cooled to RT and left overnight, whereby a yellow-orange solid precipitated out of solution. The precipitate was collected via vacuum filtration and rinsed with water (0.6808 g). Using an eluent of $CHCl_3$: ethyl acetate, 1:1, (1% triethylamine), 50.1 mg of the precipitate was purified by silica gel column chromatography (Silica Flash M60). A yellow solid (29.3 mg, 58.5% recovery yield) was collected after chromatographic separation giving an overall 25.3% yield. **^{1}H NMR** (300 MHz, d-DMSO, δ): 10.25 (s, 1H), 8.35 (d, *J* = 9.2, 2H), 8.25 (d, *J* =9.1 Hz, 2H), 7.97 (d, *J* = 8.7 Hz, 2H), 7.75 (d, *J* = 8.8 Hz, 2H), 2.06 (s, 2H). **^{13}C NMR** (126 MHz, DMSO): 170.71, 169.48, 165.50, 152.34, 151.02, 148.82,

142.87, 139.10, 127.90, 127.81, 127.66, 125.27, 119.71, 24.71. **UV-Vis λ_{max} ($CHCl_3$, $M^{-1}cm^{-1}$):** 400 nm (ε = 44,500).

Section 1.4.4: 2-(4-aminophenyl)-5-(4-nitrophenyl) thiazolo[5,4-d]thiazole (H_2N-TTz-NO_2)

AcNH-TTz-NO_2 (0.0530 g, 0.134 mmol), and 4 mL of conc. HCl were refluxed in 16 mL of n-butanol for 48 h. The reaction was cooled to room temperature, vacuum filtered and rinsed with hexanes. The crude red product was resuspended in a 0.1 M NaOH solution and heated to 70 °C for 1 h. The solution was then vacuum filtered, rinsed with water, and dried under reduced pressure to give a red/orange solid, 0.0185 g (39.4% yield). **^{1}H NMR** (300 MHz, DMSO, δ): 8.34 (d, *J* = 9.2 Hz, 2H), 8.22 (d, *J* = 9.2, 2H), 7.70 (d, *J* = 8.6 Hz, 2H), 6.64 (d, *J* = 8.7 Hz, 2H), 5.99 (s, 2H). **^{13}C NMR** (126 MHz, DMSO): 172.50, 163.62, 153.17, 152.38, 149.69, 148.53, 139.36, 128.66, 127.31, 125.30, 125.24, 120.63, 114.21. **UV-vis λ_{max} ($CHCl_3$, ε = M^{-1} cm^{-1}): 409 nm (ε = 12,600) MALDI-TOF-MS:** calcd for $C_{16}H_{10}N_4O_2S_2$: 354.402; found, 354.536.

Section 1.4.5: Bis-(4-acetamidophenyl)-thiazolo[5,4-d]thiazole ($(AcNH)_2$TTz)

Dithiooxamide (0.9263 g, 7.707 mmol) and 4-acetamidobenzaldehyde (3.1894 g, 19.546 mmol) were heated in 40 mL of DMF at 140 ^{o}C for 8 h in an aerated environment. The reaction was cooled to room temperature and left to sit in a fridge overnight. The solution was then vacuum filtered and rinsed with cooled ether and hexanes. The solid was dried in a vacuum oven and gave a bright yellow solid (1.2082 g, 38.9% yield). **^{1}H-NMR** (300 MHz, d-DMSO), δ 10.20 (s, 2H), 7.92 (d, J = 8.7 Hz, 4H), 7.72 (d, J =8.7 Hz, 4H), 2.05 (s, 6H) ppm. **^{13}C NMR** (126 MHz, DMSO): 169.30, 168.60, 150.20, 142.41, 128.23, 127.46, 119.72. **UV-vis λ_{max} ($CHCl_3$, ε = M^{-1} cm^{-1}): 380 nm (ε = 14,100) MALDI-TOF-MS:** calcd for $C_{20}H_{16}N_4O_2S_2$ 408.494; found, 408.517. **Φ_{CHCl3}** = 0.367

Section 1.4.6: Bis-(4-aminophenyl)-thiazolo[5,4-d]thiazole ($(H_2N)_2TTz$)

$(AcNH)_2TTz$ (0.5058 g, 1.572 mmol) was added to 165.5 mL of n-butanol and 33.75 mL conc. HCl and heated to 100 ºC for 96 h. The solution was vacuum filtered and rinsed with hexanes. The solid was dried via vacuum oven, dissolved in 0.1 M NaOH, and heated to 50 ºC for 1 h. The solution was then vacuum filtered and rinsed with water. The product was vacuum dried and placed in a Schlenk tube where it was sublimed under vacuum overnight at 250 ºC. The product on the cold finger was collected to give a bright yellow solid (365.1 mg, 90.9% yield). ^{1}H-NMR (300 MHz, d-ACN), δ 7.70 (d, J = 8.9 Hz, 4H), 6.69 (d, J = 8.9 Hz, 4H), 4.62 (s, 4H) ppm. **UV-vis λ_{max} ($CHCl_3$, ε = M^{-1} cm^{-1}): 389 nm (ε = 72,600)** **MALDI-TOF-MS:** calcd for $C_{16}H_{12}N_4S_2$ 324.050; found, 324.955. $\mathbf{\Phi_{CHCl_3}}$ = 0.193

Section 2: Spectra Characterization

Section 2.1: NMR

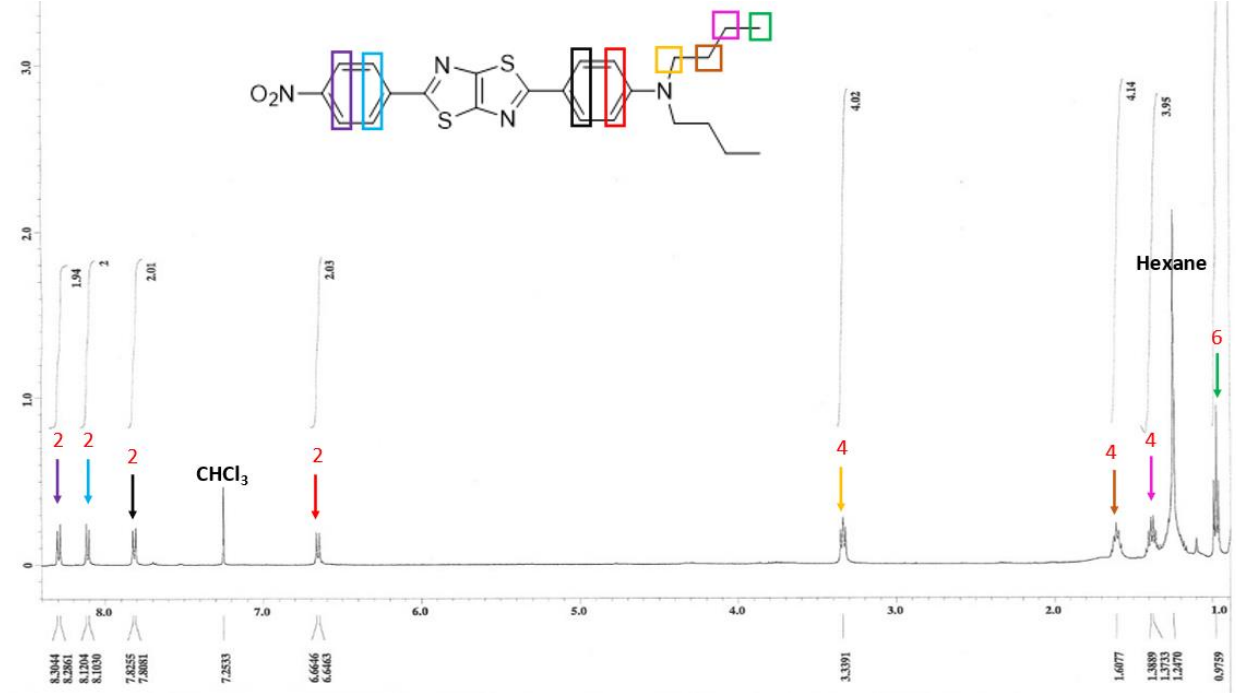

Figure S1: ^{1}H NMR of Bu_2N-TTz-NO_2.

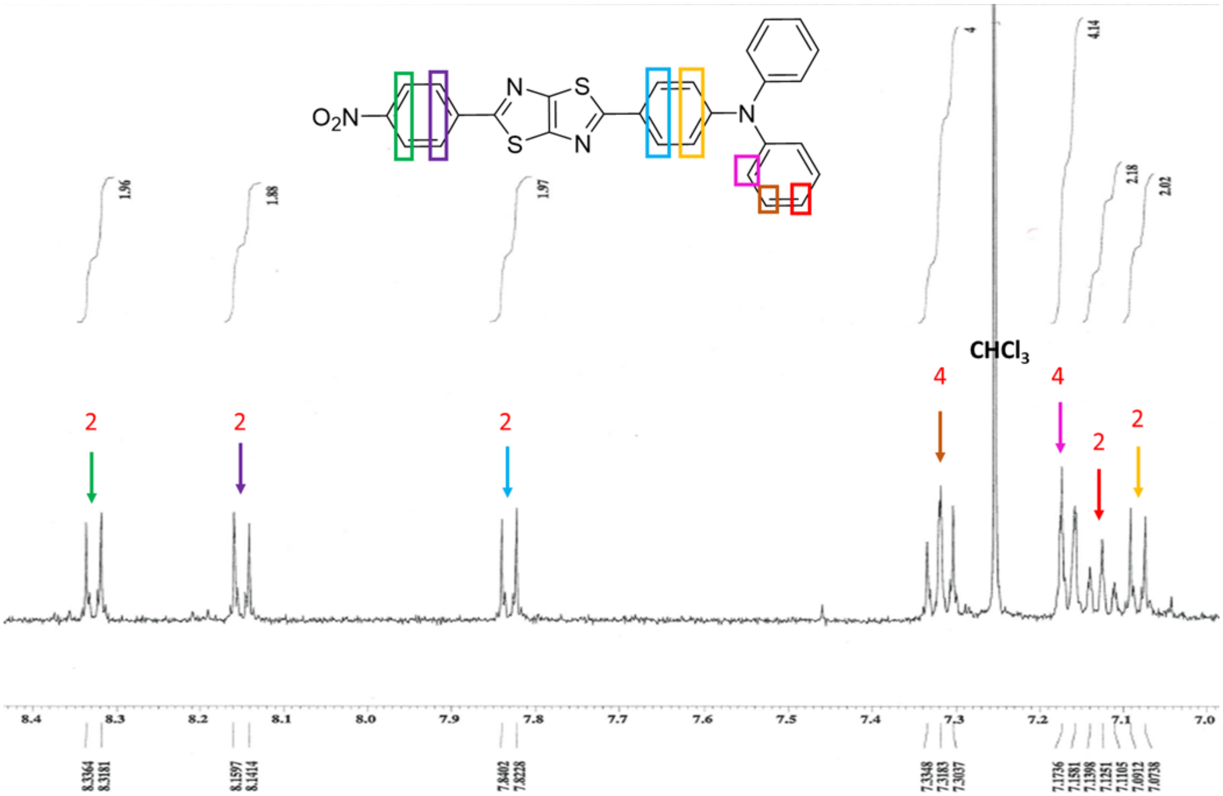

Figure S2: ^{1}H NMR of Ph_2N-TTz-NO_2.

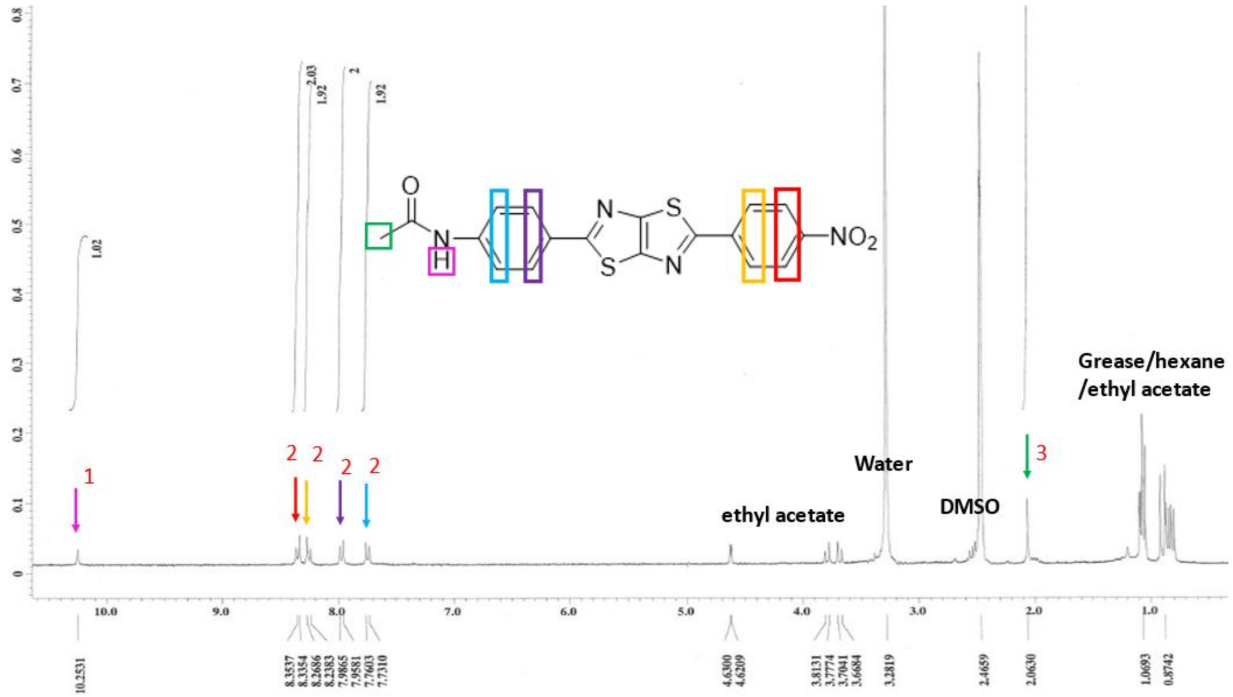

Figure S3: ^{1}H NMR of AcNH-TTz-NO_2.

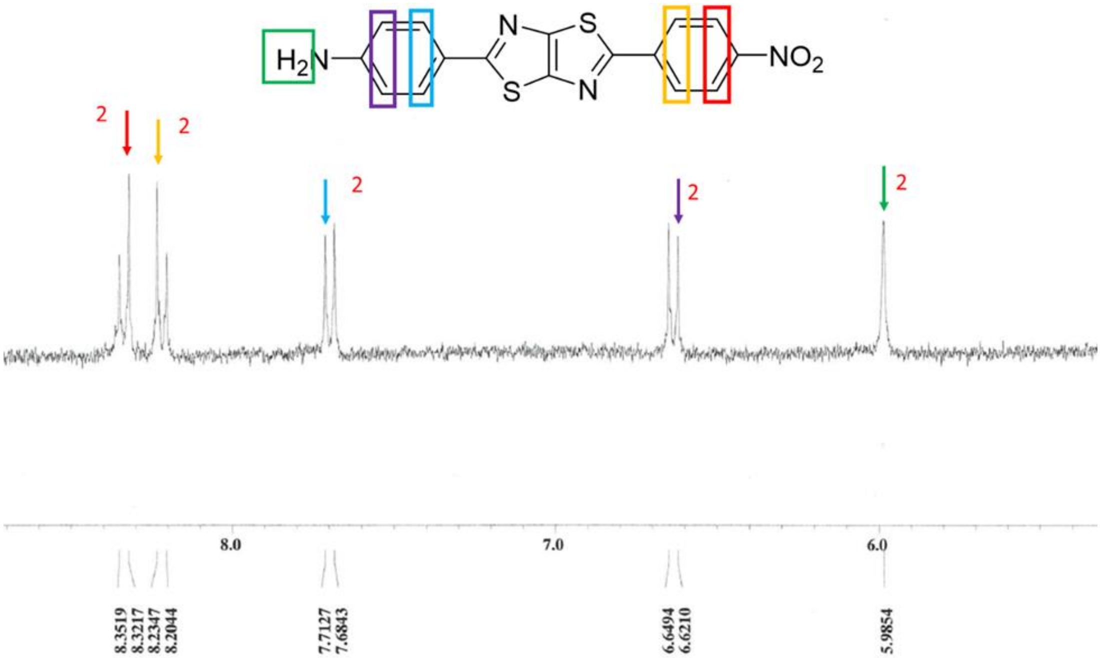

Figure S4: ^{1}H NMR of H_2N-TTz-NO_2.

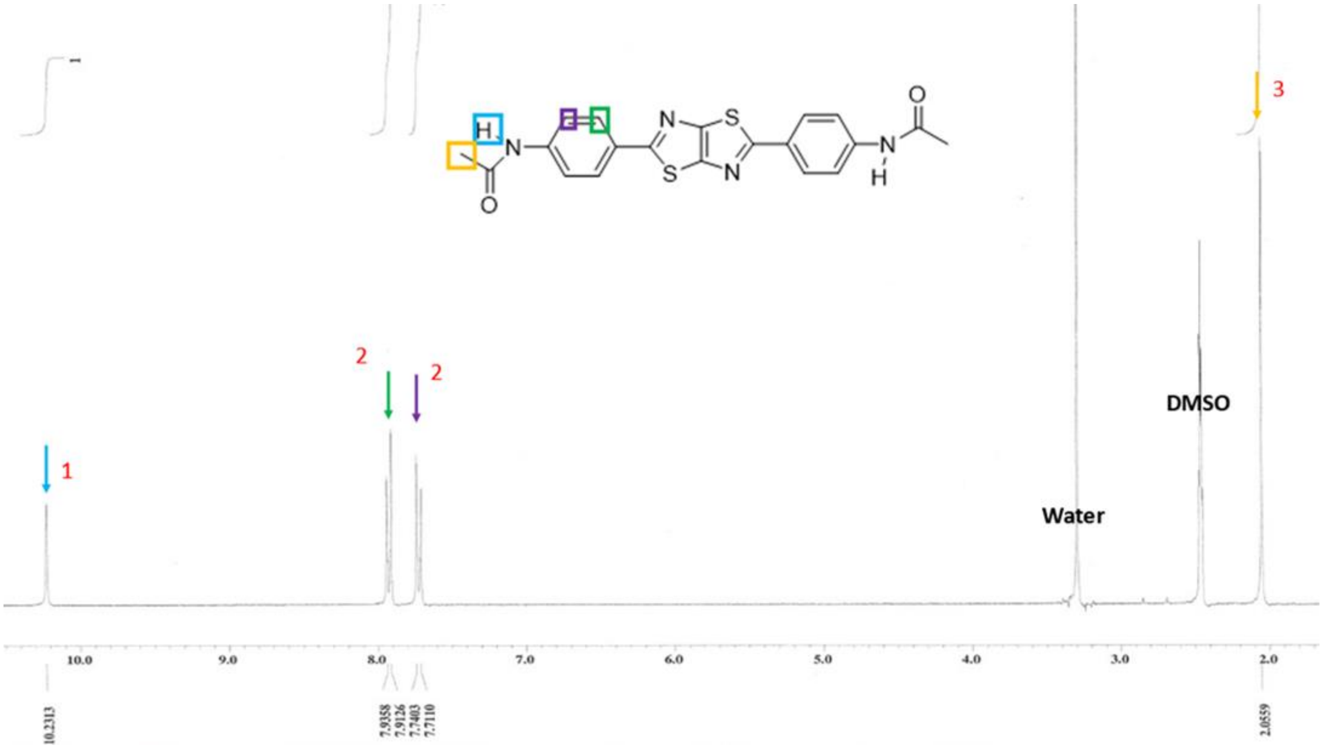

Figure S5: ^{1}H NMR of $(AcNH)_2$-TTz.

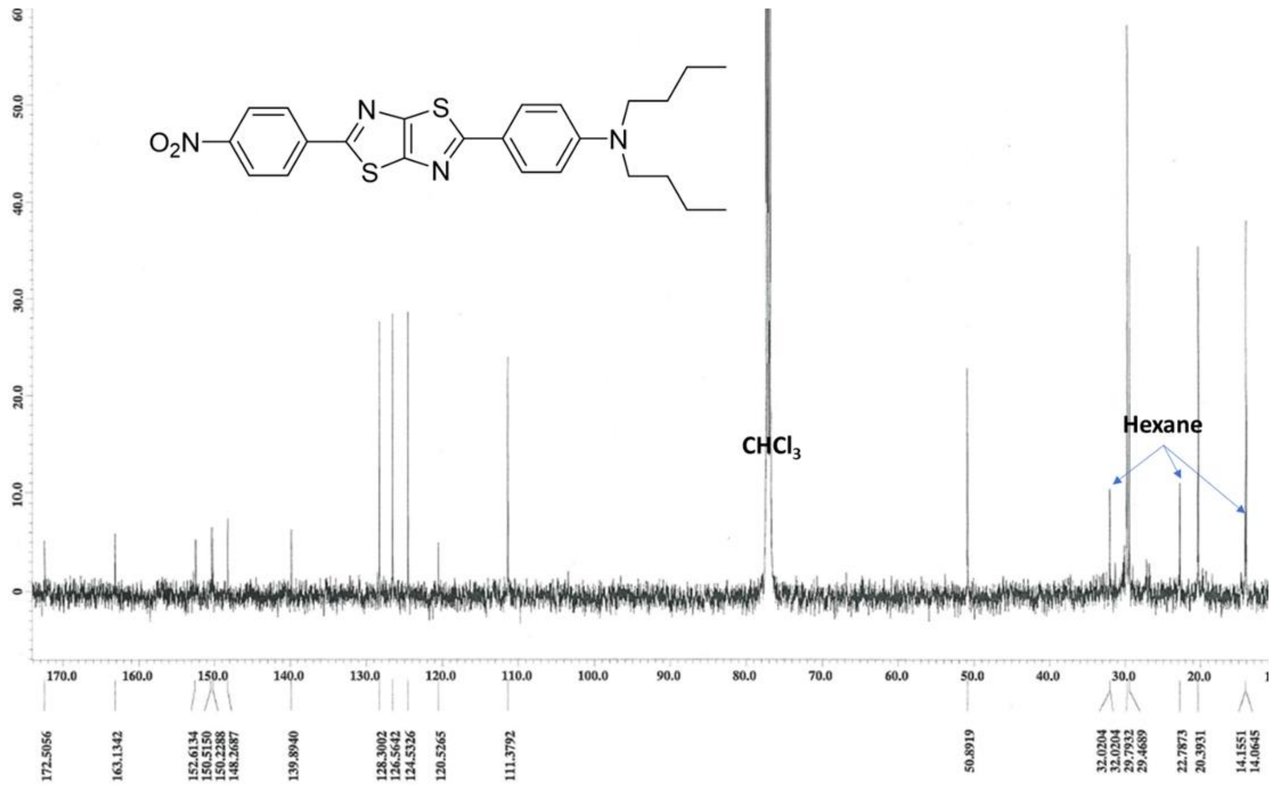

Figure S6: ^{13}C NMR of Bu_2N-TTz-NO_2

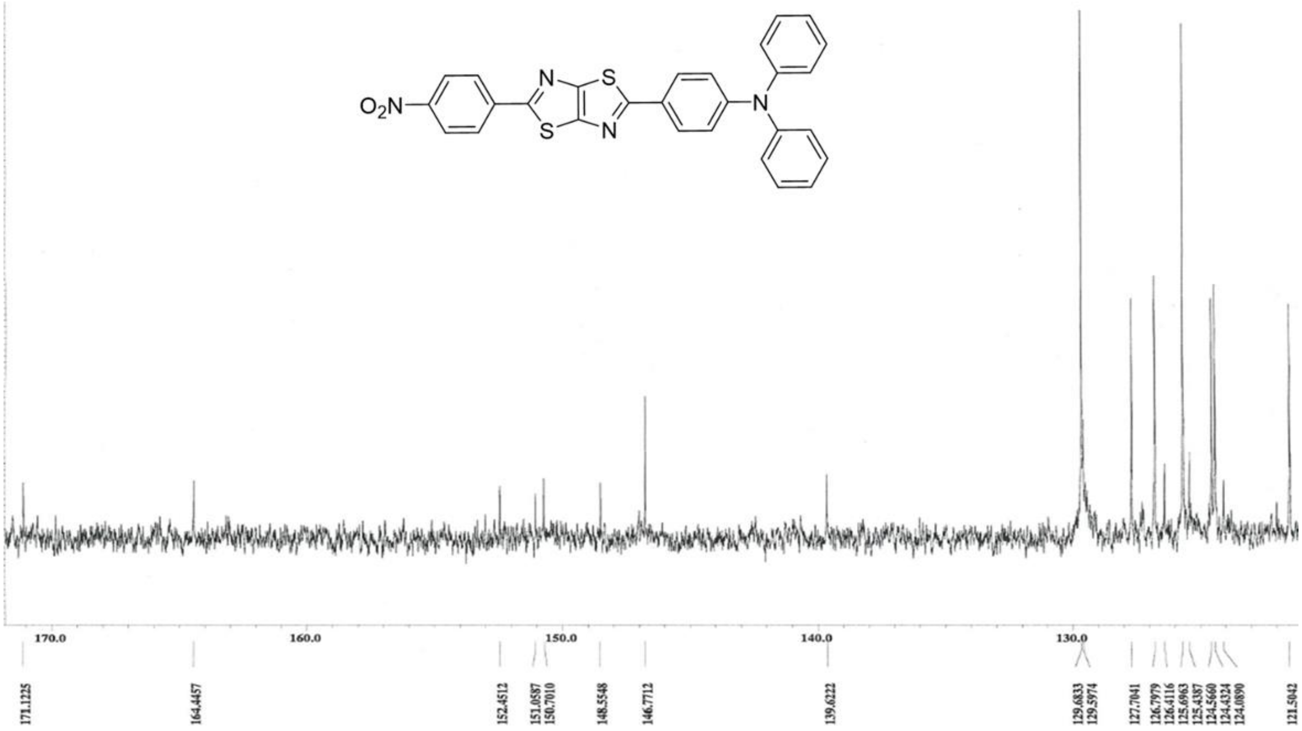

Figure S7: ^{13}C NMR of Ph_2N-TTz-NO_2.

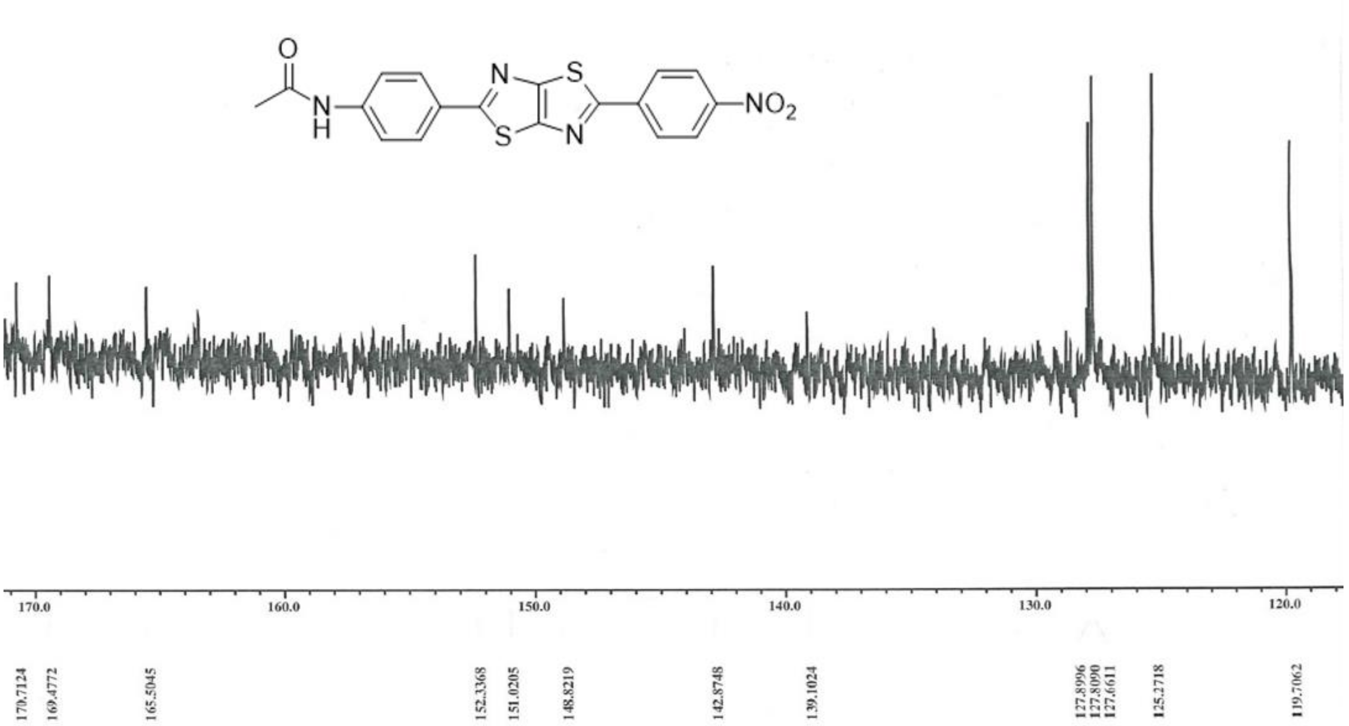

Figure S8: ^{13}C NMR of AcNH-TTz-NO_2. Peak at **24.7092** not shown for succinctness. Similar to $(AcNH)_2$TTz.

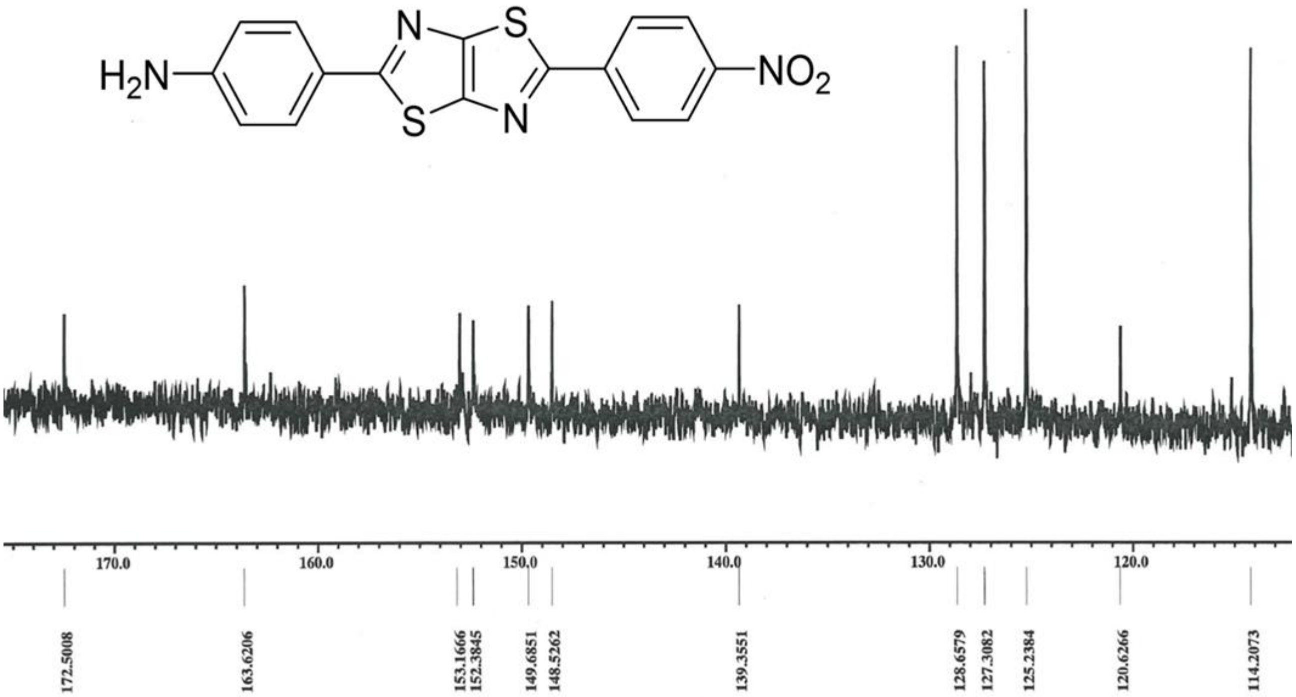

Figure S9: ^{13}C NMR of H_2N-TTz-NO_2.

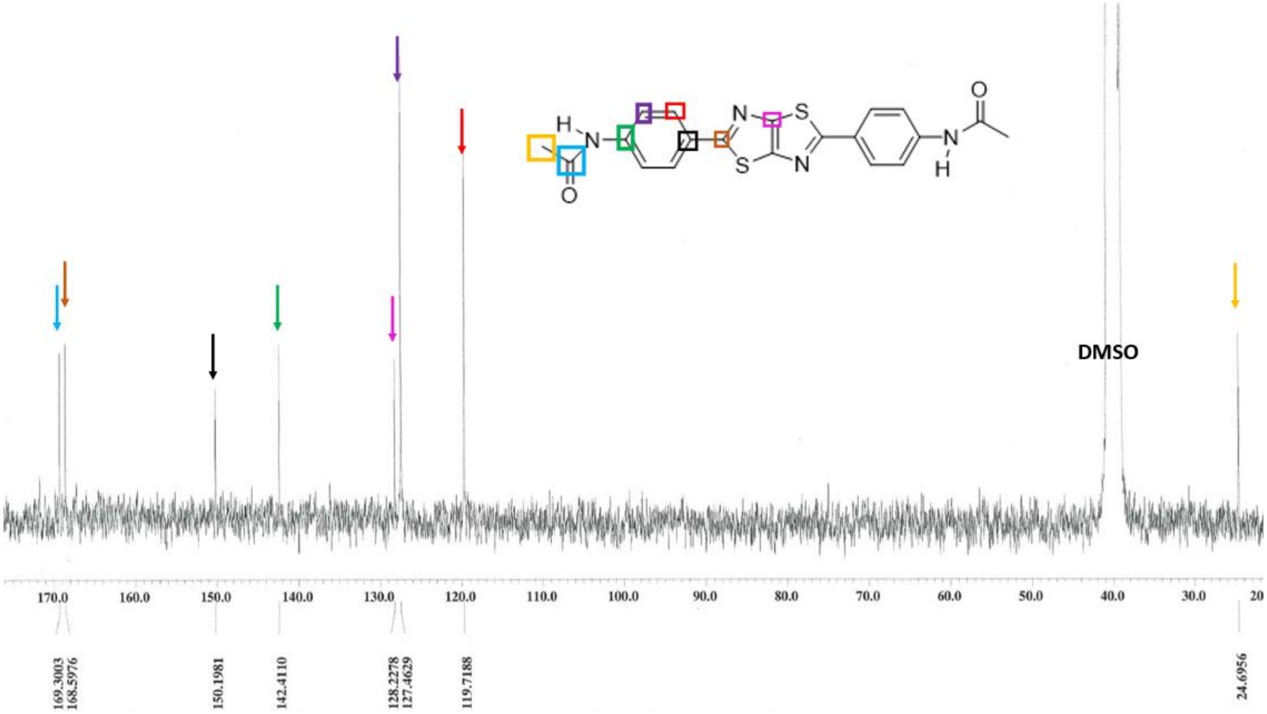

Figure S10: ^{13}C NMR of $(AcNH)_2$-TTz.

Section 2.2: MALDI-MS

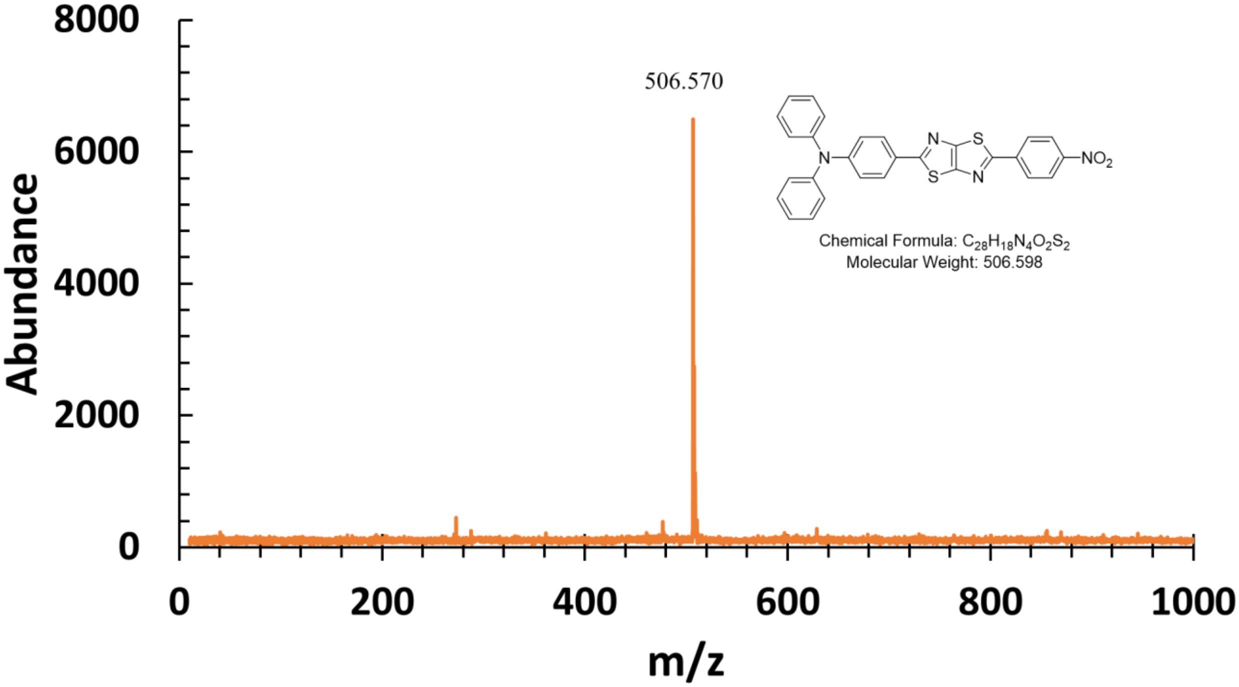

Figure S11: MALDI-MS of Ph_2N-TTz-NO_2.

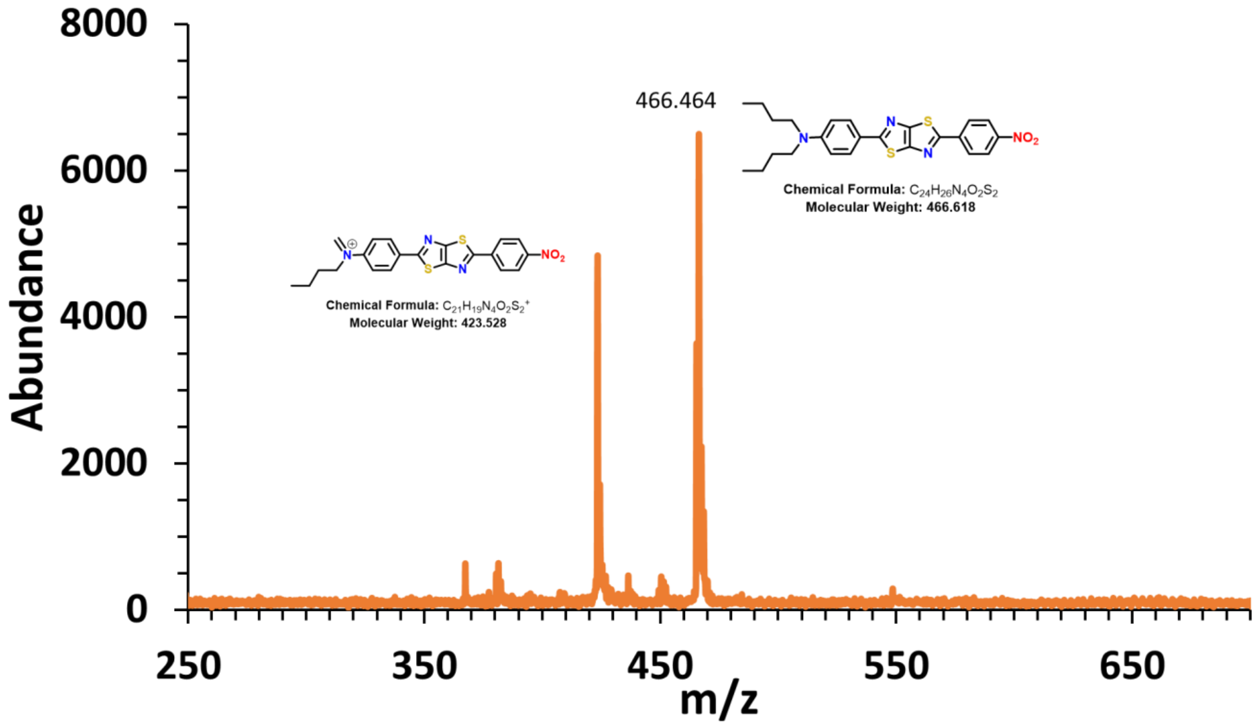

Figure S12: MALDI-MS of Bu_2N-TTz-NO_2.

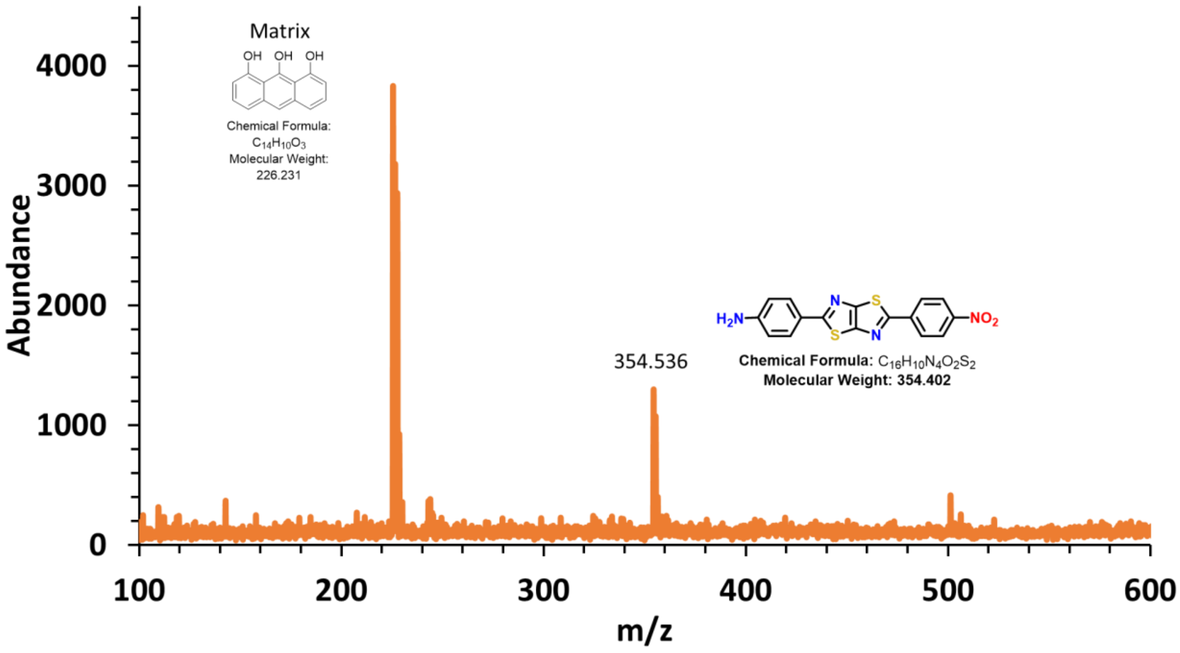

Figure S13: MALDI-MS of H_2N-TTz-NO_2.

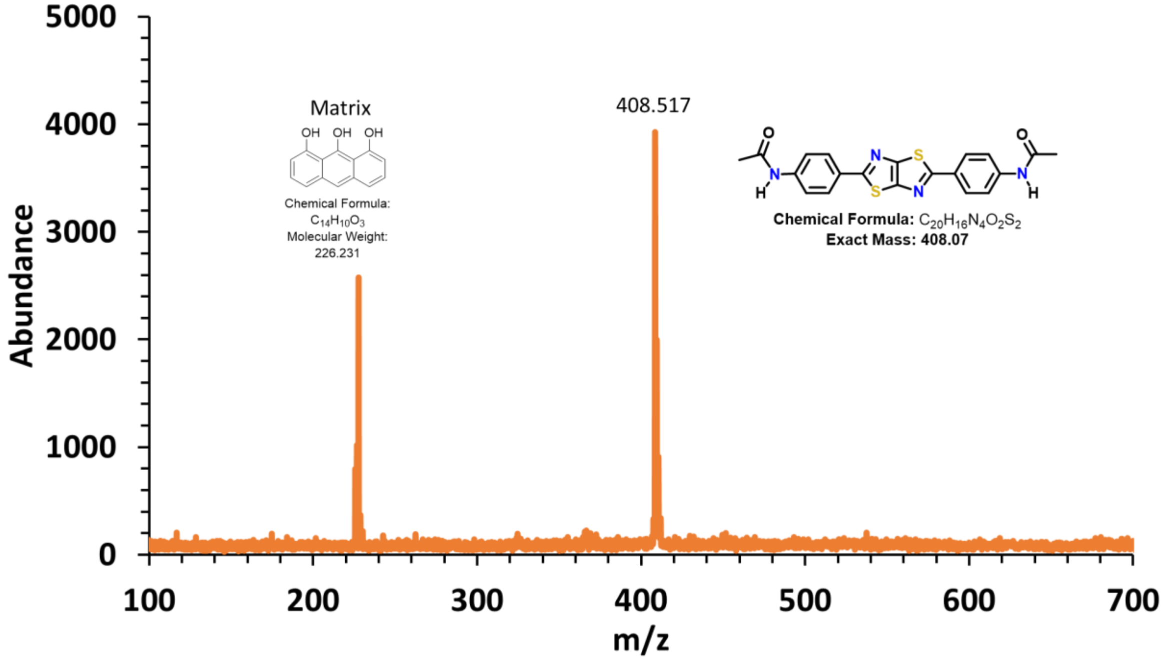

Figure S14: MALDI-MS of $(AcNH)_2$-TTz.

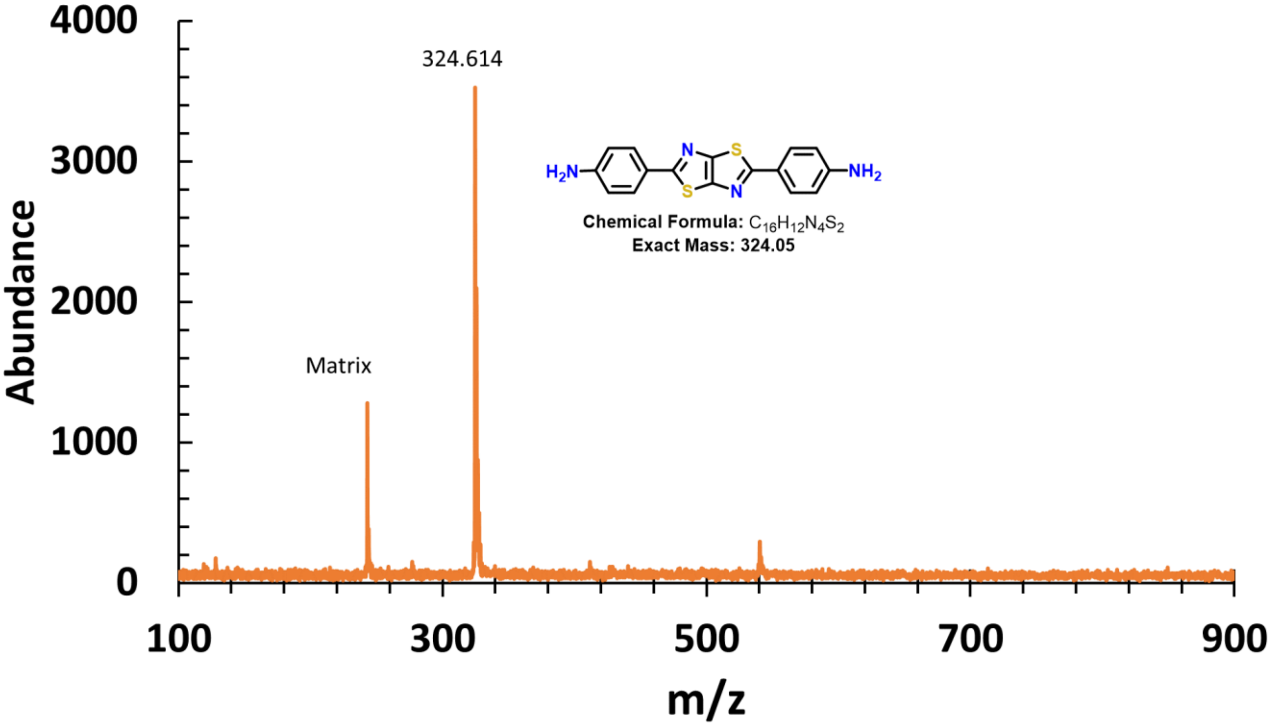

Figure S15: MALDI-MS of $(NH)_2$-TTz.

Section 2.3: Optical Properties of amino/nitrophenyl aTTz Dyes in Various Solvents

Table S1. Optical Properties of amino/nitrophenyl aTTz Dyes in Various Solvents

		Abs (nm)	Abs (eV)	Ext. coeff. $(M\cdot cm)^{-1}$	Em (nm)	Em (eV)	Stokes Shift (eV)	%QY	Fluorescence Lifetime (ns)	Radiative rate (sec^{-1})	Non-radiative rate (sec^{-1})
dye	solvent	λ_{max}	E_{ex}	ε	λ_{max}	E_{em}	SS	Φ_F	τ_F	k_r	k_{nr}
Bu_2N-TTz-NO_2	**Hex**	447	2.77	13000	499	2.48	0.29	53.5	2.18	2.45×10^8	2.13×10^8
	**Tol**	458	2.71	24300	561	2.21	0.50	42.2	2.74	1.55×10^8	2.10×10^8
	**Bz**	458	2.71	31800	563	2.20	0.51	39.5	2.62	1.51×10^8	2.31×10^8
	**Cl-Bz**	467	2.66	17700	614	2.02	0.64	0.8	1.41	5.62×10^6	7.04×10^8
	**THF**	456	2.72	22300	504	2.47	0.25	0.6	1.83	3.28×10^6	5.43×10^8
	**Bz-CN**	469	2.64	20220	518	2.39	0.25	0.2	1.22	1.64×10^6	8.18×10^8
	**$CHCl_3$**	462	2.68	19900	487	2.55	0.13	3.1	1.14	2.72×10^7	8.50×10^8
	**EtOH**	437	2.84	2200	488	2.54	0.30	6.4	1.34	4.78×10^7	6.99×10^8
Ph_2N-TTz-NO_2	**Hex**	438	2.83	11100	489	2.54	0.29	69.2	2.26	1.36×10^8	3.06×10^8
	**Tol**	444	2.79	46900	547	2.27	0.52	63.1	2.87	1.89×10^8	1.60×10^8
	**Bz**	447	2.77	19300	550	2.25	0.52	56.8	2.69	2.11×10^8	1.61×10^8
	**Cl-Bz**	453	2.74	45500	592	2.09	0.65	2.9	1.78	1.63×10^7	5.46×10^8
	**THF**	444	2.79	11700	497	2.49	0.30	0.6	1.69	3.55×10^6	5.88×10^8
	**Bz-CN**	447	2.77	58600	501	2.48	0.29	0.4	1.78	2.25×10^6	5.60×10^8
	**$CHCl_3$**	446	2.78	11400	487	2.55	0.23	5.0	1.37	3.65×10^7	6.93×10^8
	**EtOH**	436	2.84	3000	499	2.48	0.36	13.1	1.27	1.03×10^8	6.84×10^8
AcNH-TTz-NO_2	**Tol**	398	3.12	21000	490	2.53	0.59	9.3	1.48	6.38×10^7	6.13×10^8
	**Bz**	398	3.12	26000	493	2.52	0.60	10.9	1.05	1.04×10^8	8.49×10^8
	**Cl-Bz**	402	3.08	7000	526	2.36	0.72	22.1	2.32	9.53×10^7	3.36×10^8
	**THF**	397	3.12	33900	533	2.33	0.79	17.9	1.89	9.47×10^7	4.34×10^8
	**Bz-CN**	404	3.07	14300	461 (558)	2.70 (2.22)	0.37 (0.85)	4.9	1.27	3.86×10^7	7.49×10^8
	**$CHCl_3$**	400	3.10	44500	557	2.23	0.87	8.5	1.72	4.94×10^7	5.32×10^8
H_2N-TTz-NO_2	**Tol**	408	3.04	17000	544	2.28	0.76	1.8	2.51	7.17×10^6	1.63×10^8
	**Bz**	408	3.04	9000	544	2.28	0.76	2.7	2.15	1.26×10^7	4.53×10^8
	**Cl-Bz**	412	3.01	31000	507	2.45	0.56	2.1	1.66	1.29×10^7	5.90×10^8
	**THF**	407	3.05	14700	508	2.44	0.61	1.1	1.42	7.76×10^6	6.96×10^8
	**Bz-CN**	412	3.01	9900	509	2.44	0.66	2.2	1.79	1.23×10^7	5.43×10^8
	**$CHCl_3$**	409	3.03	12600	529	2.34	0.69	1.5	2.07	7.25×10^6	4.76×10^8

*Solvents were chosen to span a wide range of polarizability factors ($\Delta f, see\ Eq. 1$). For direct comparisons, similar solvents were used when possible; however, solubility differences between the compounds prohibited complete standardization in this regard. To compensate, alternative solvents were selected based on solubility and Δf proximity.
** Emissions in parentheses are not maximum emission wavelengths but still prominent ICT emission.

Section 3: Solvent and Temperature Sensitivity

Section 3.1: Absorbance and Emission

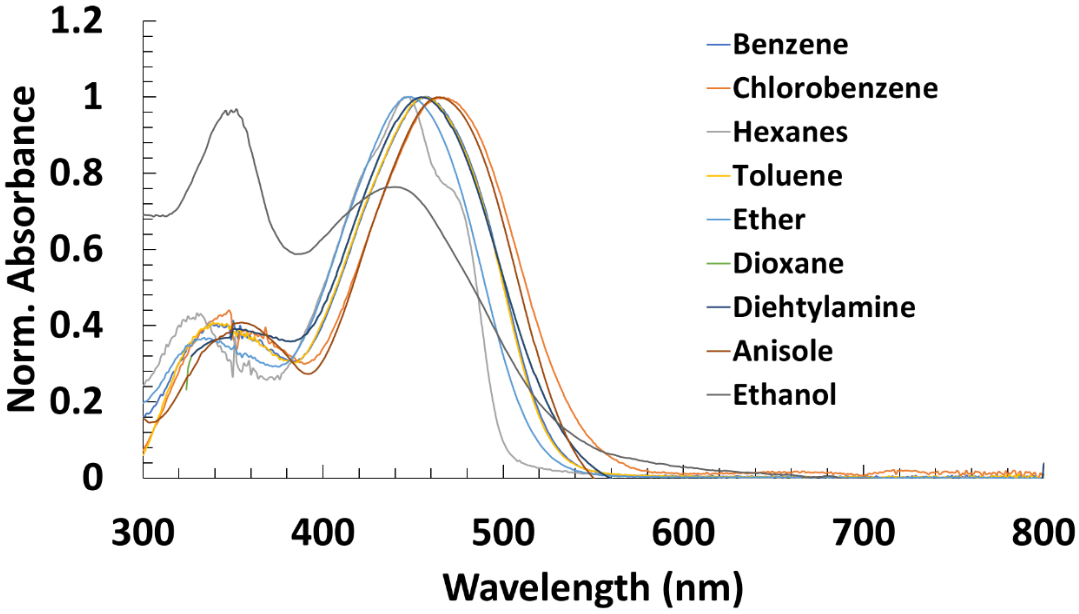

Figure S16: Normalized absorbance of Bu_2N-TTz-NO_2 in multiple solvents.

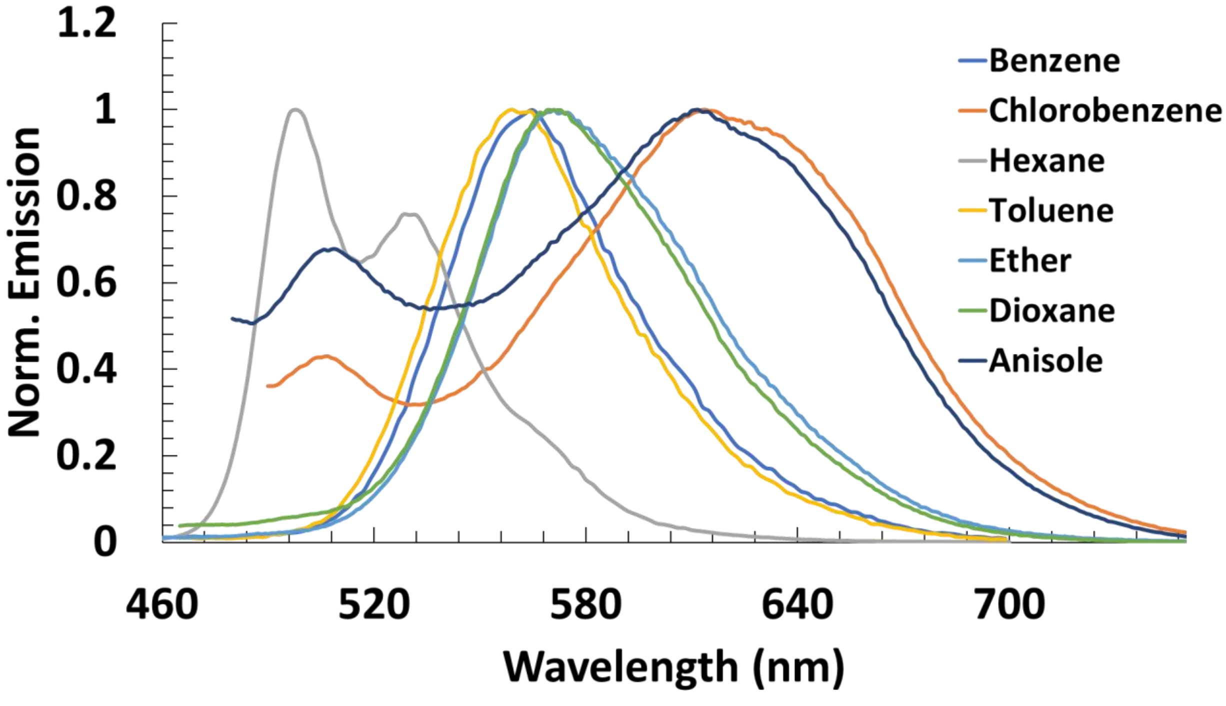

Figure S17: LWB normalized emission of Bu_2N-TTz-NO_2 multiple solvents.

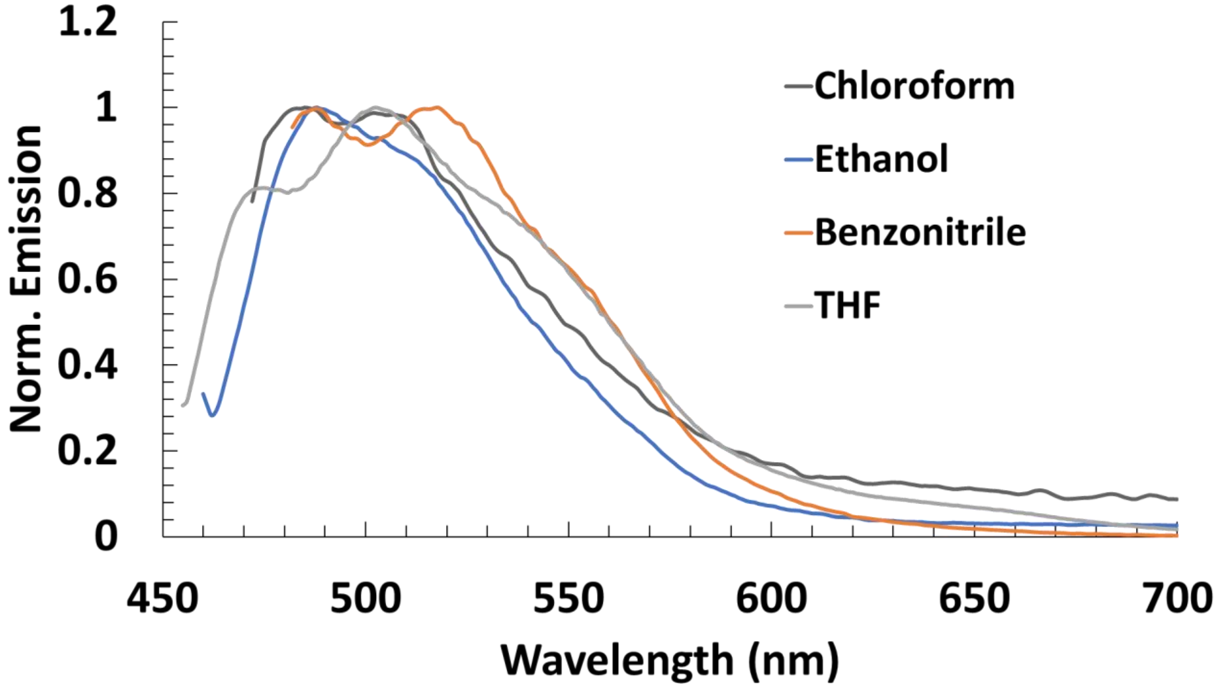

Figure S18: SWB normalized emission of Bu_2N-TTz-NO_2 multiple solvents.

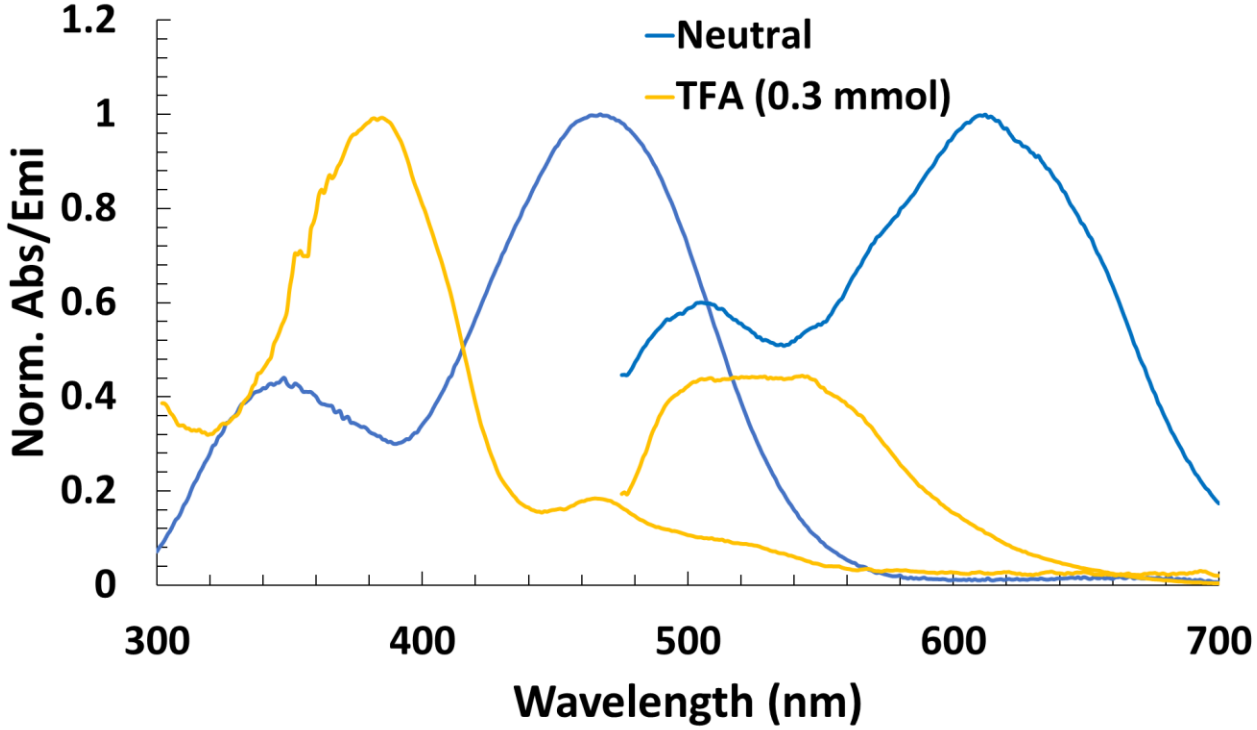

Figure S19: Absorbance and emission of Bu_2N-TTz-NO_2 in ClBz with excess and without TFA (neutral).

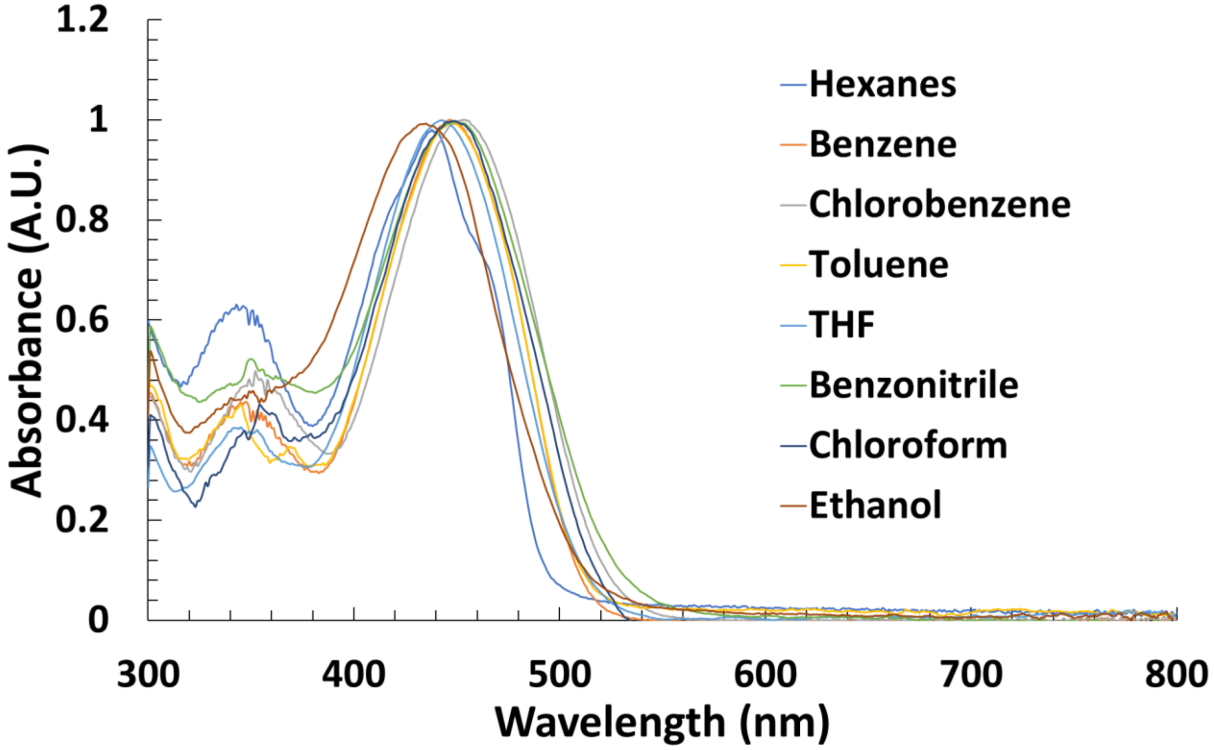

Figure S20: Normalized absorbance of Ph_2N-TTz-NO_2 in multiple solvents.

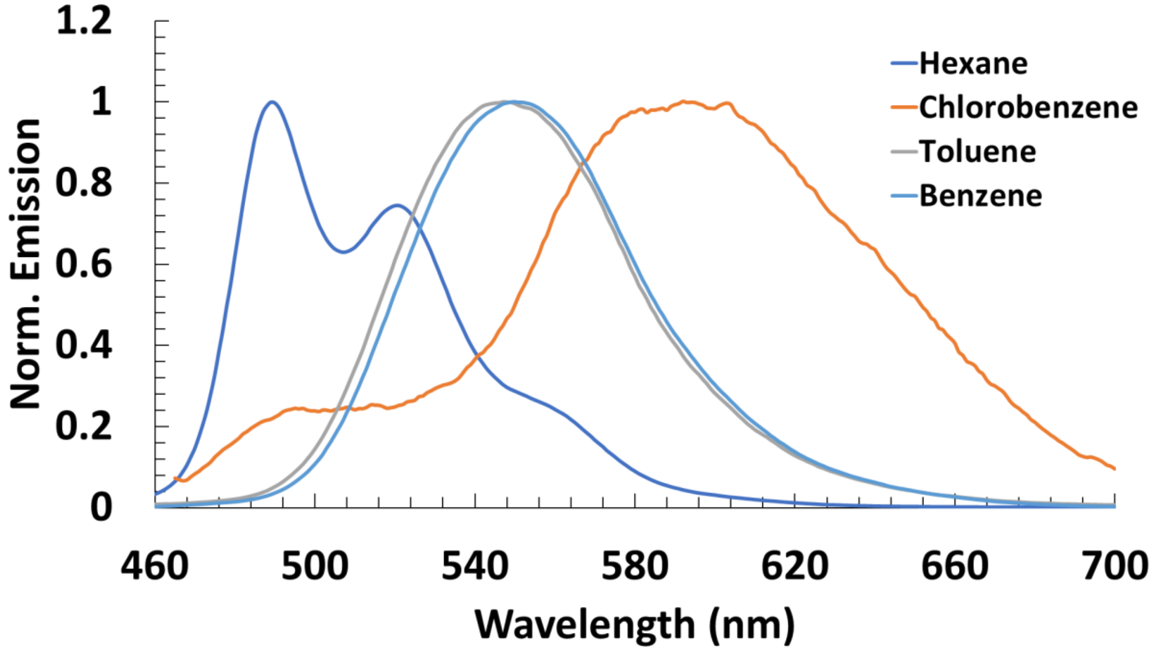

Figure S21: LWB normalized emission of Ph_2N-TTz-NO_2 in various solvents.

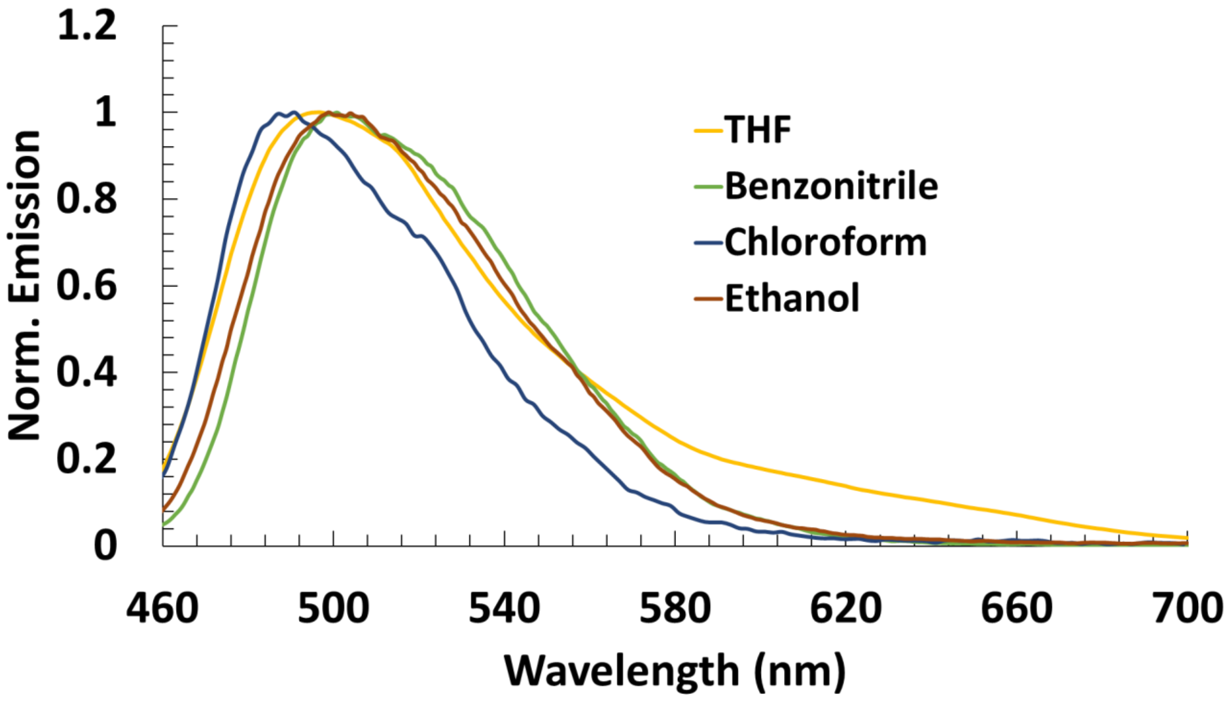

Figure S22: SWB normalized emission of Ph_2N-TTz-NO_2 in various solvents.

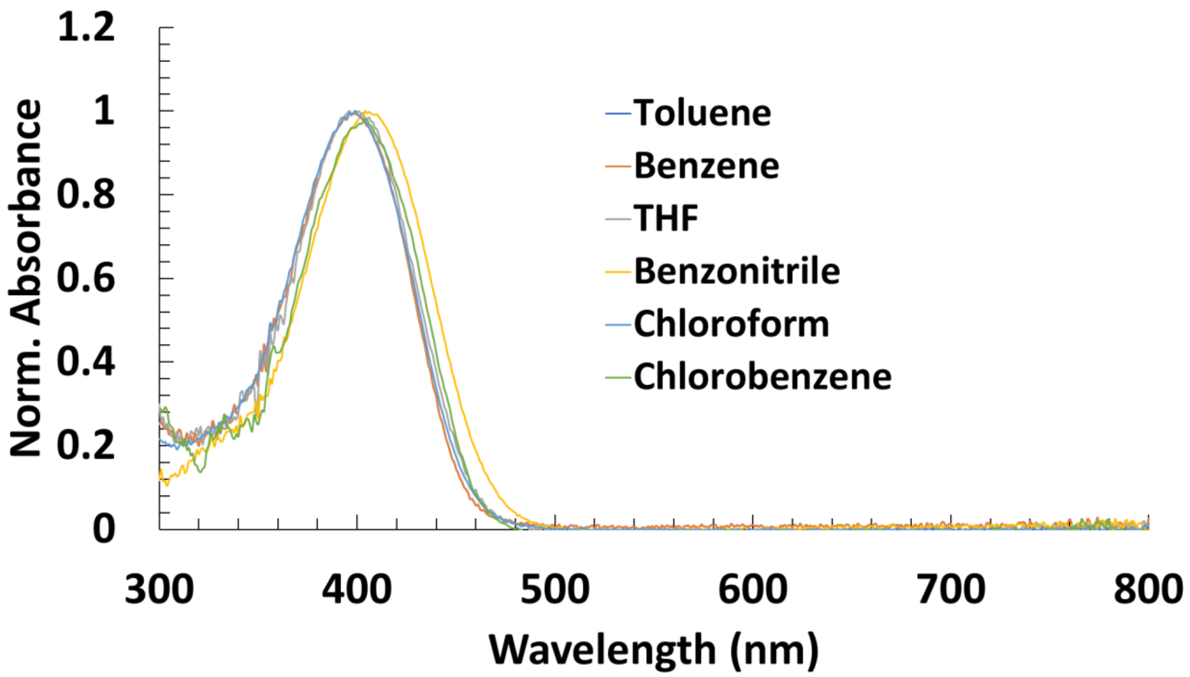

Figure S23: Normalized absorbance of AcNH-TTz-NO_2 in various solvents.

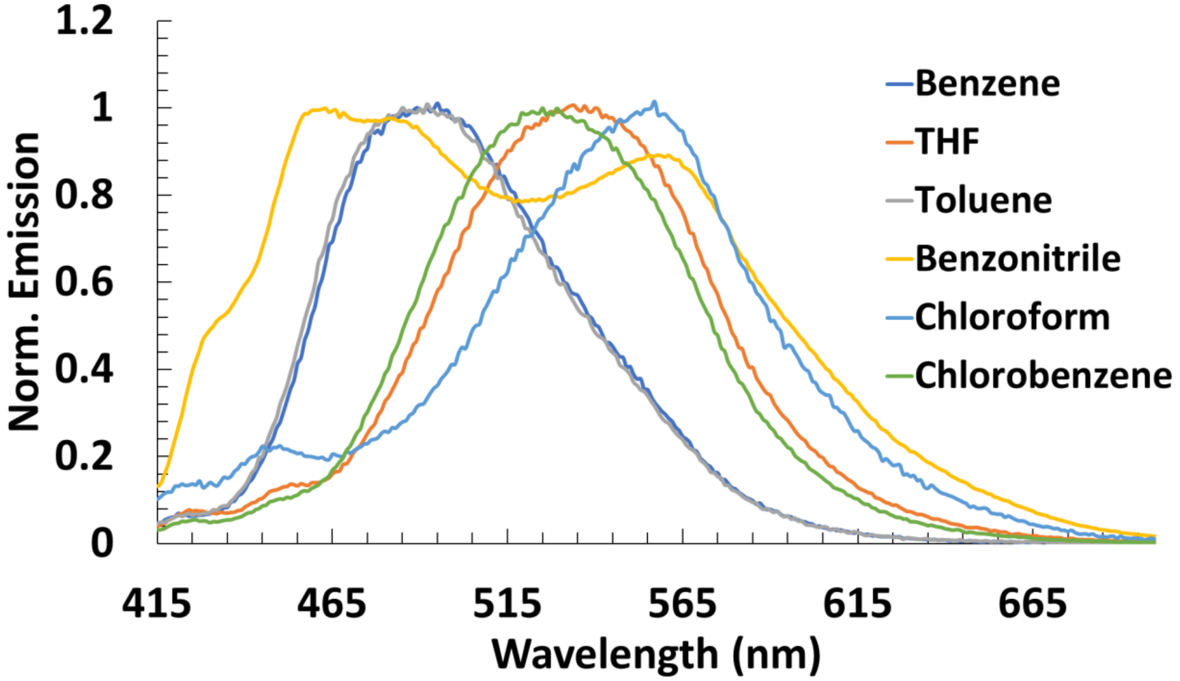

Figure S24: Normalized emission of AcNH-TTz-NO_2 in various solvents.

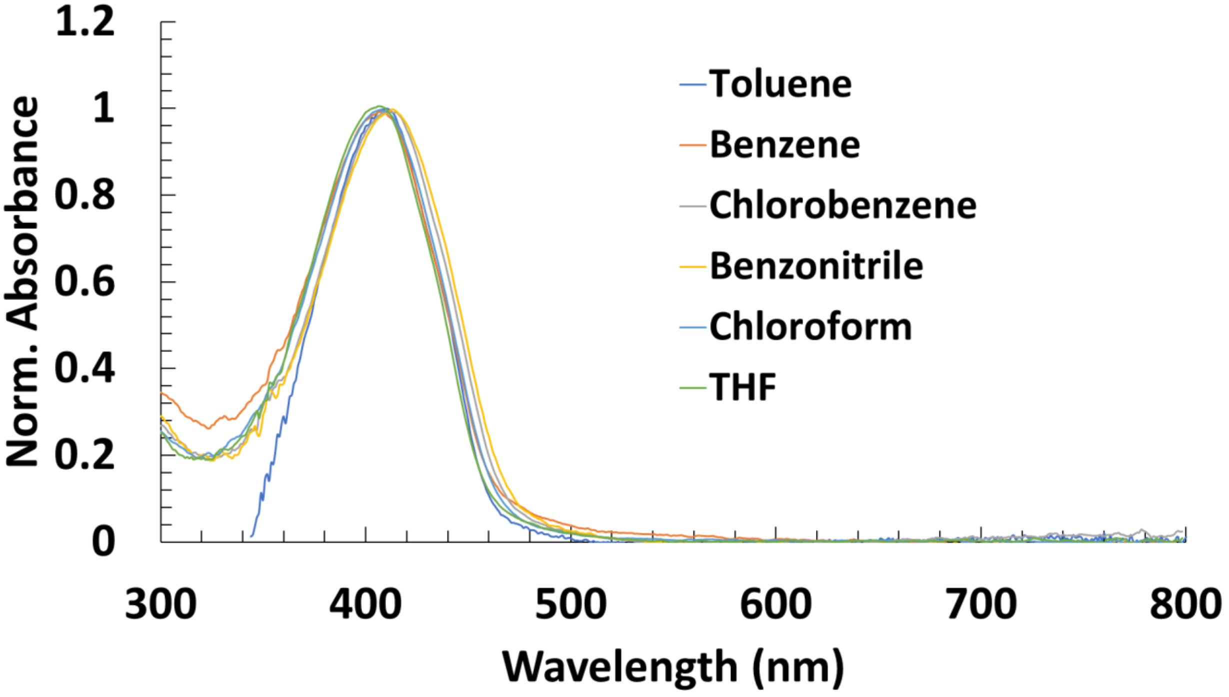

Figure S25: Absorbance of H_2N-TTz-NO_2 in various solvents.

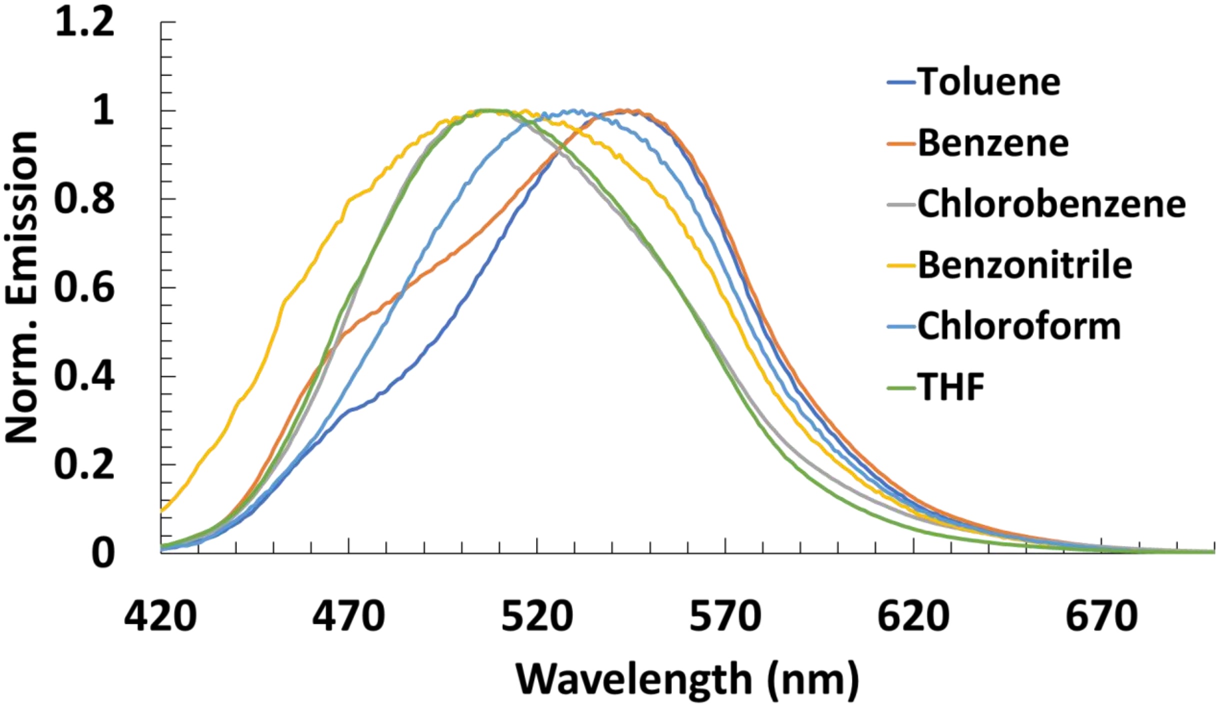

Figure S26: Emission of H_2N-TTz-NO_2 in various solvents.

Section 3.2: Fluorescence Lifetimes

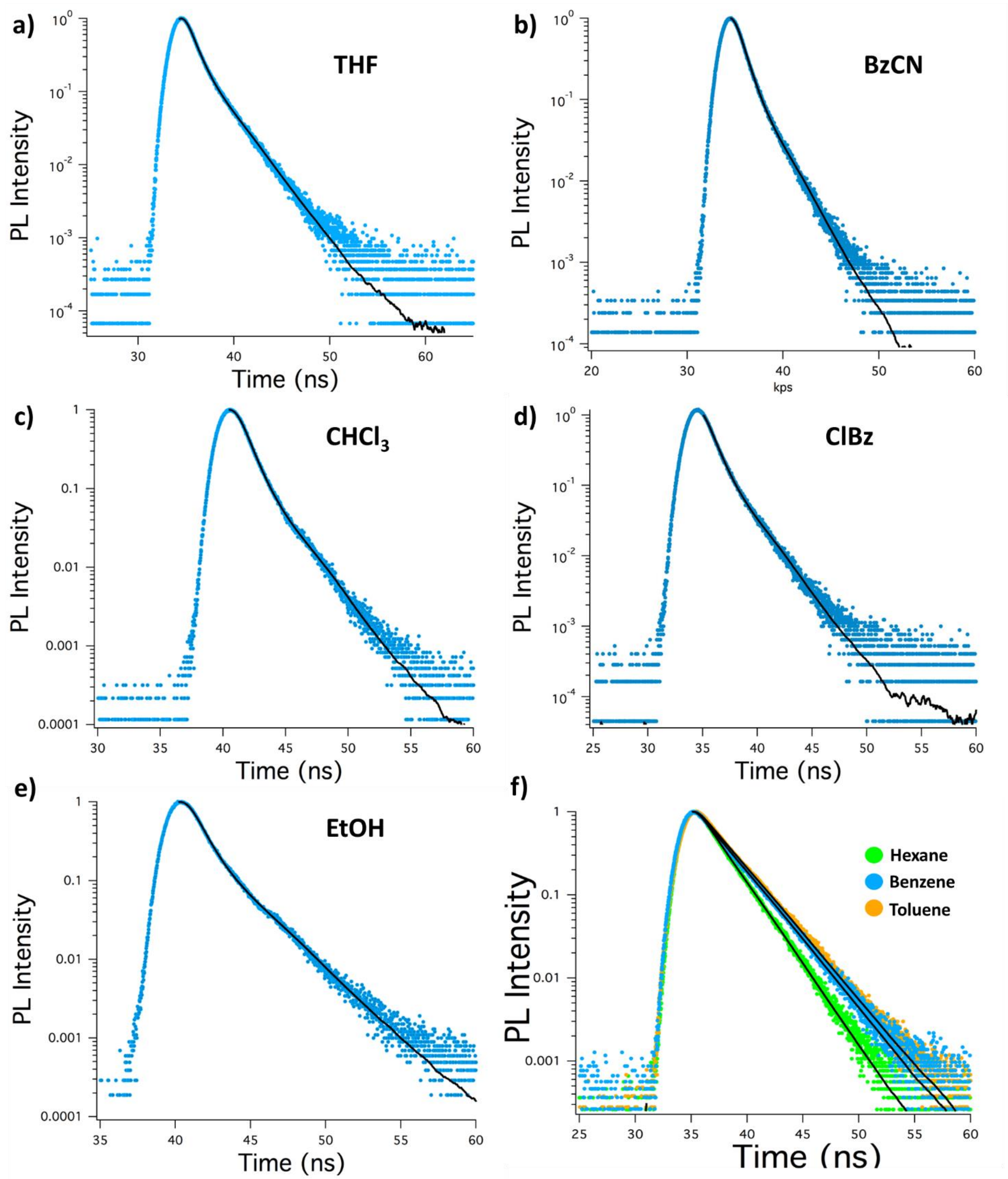

Figure S27: Fluorescence Lifetimes of Bu_2N-TTz-NO_2 in various solvents.

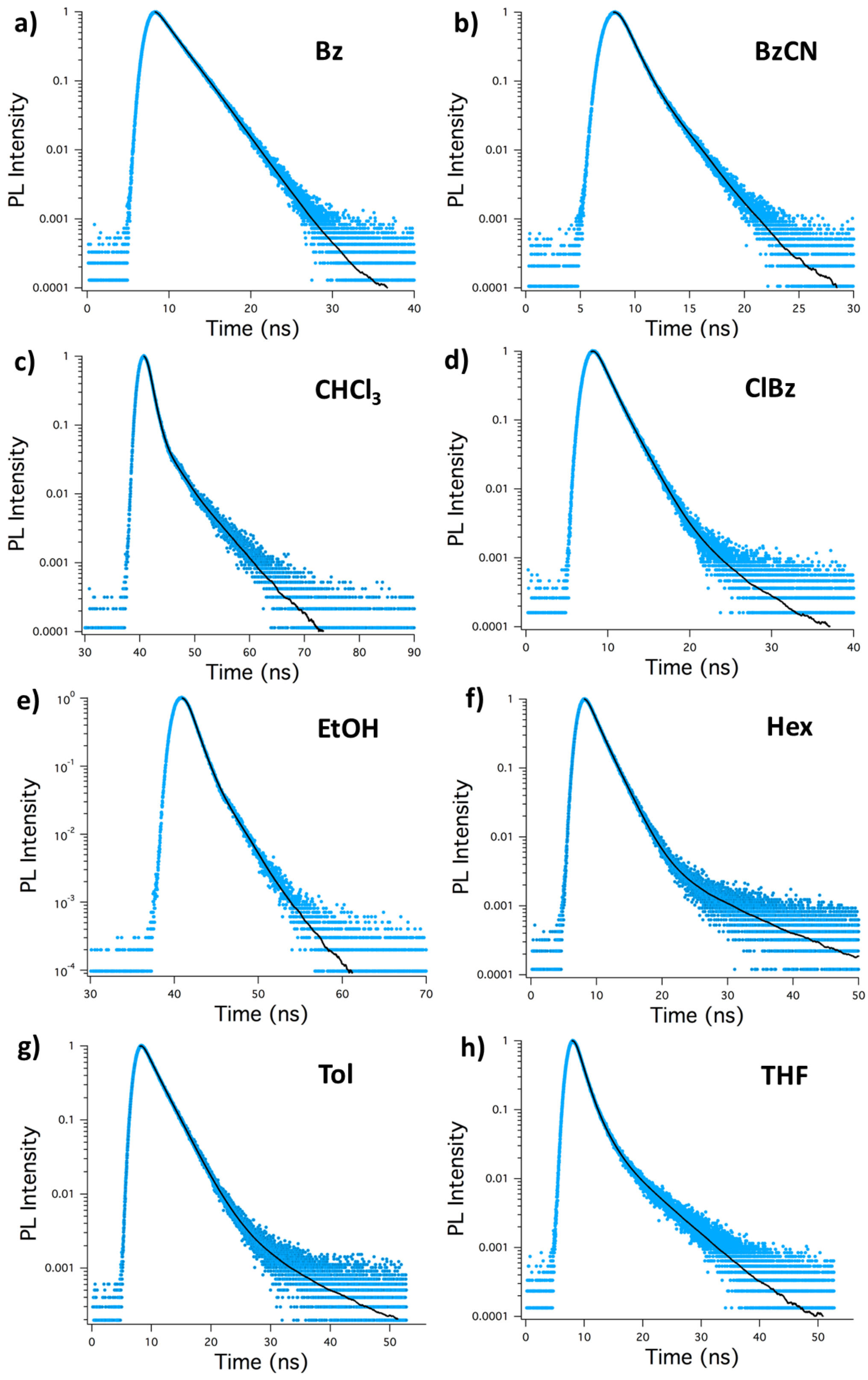

Figure S28: Fluorescence Lifetimes of Ph_2N-TTz-NO_2 in various solvents.

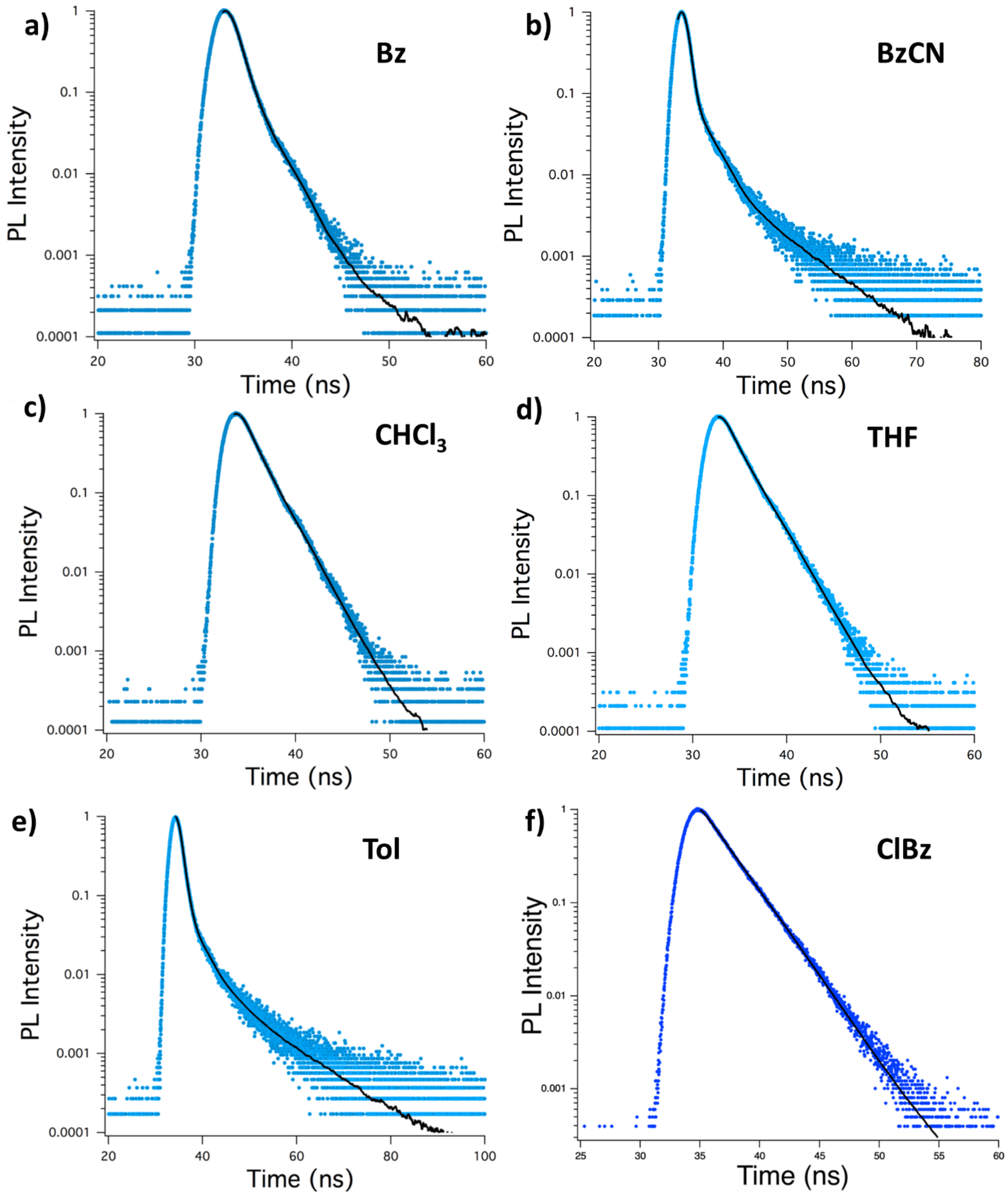

Figure S29: Fluorescence Lifetimes of AcNH-TTz-NO_2 in various solvents.

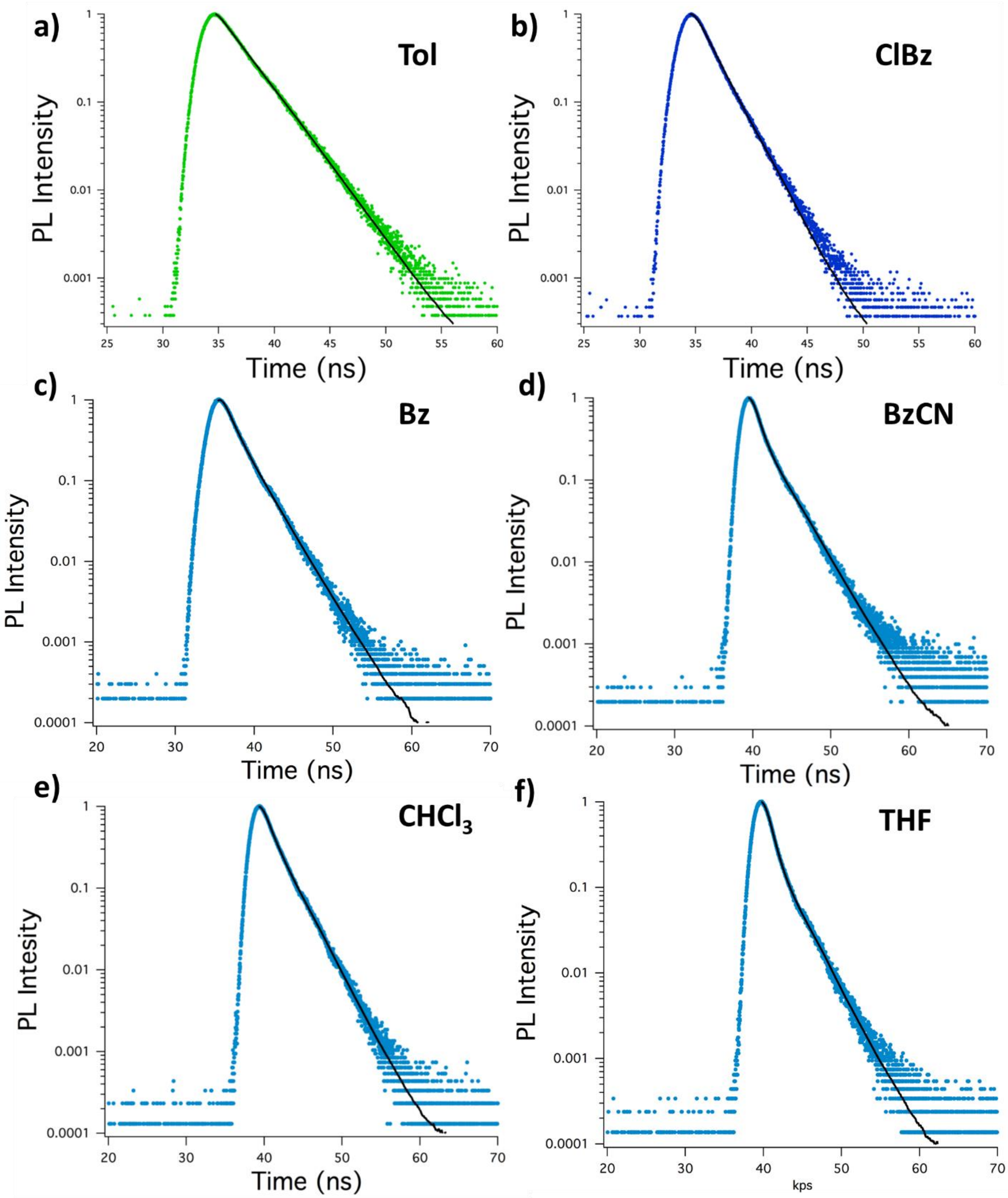

Figure S30: Fluorescence Lifetimes of H_2N-TTz-NO_2 in various solvents.

Section 3.3: Quantum Yield and Molar Absorptivity

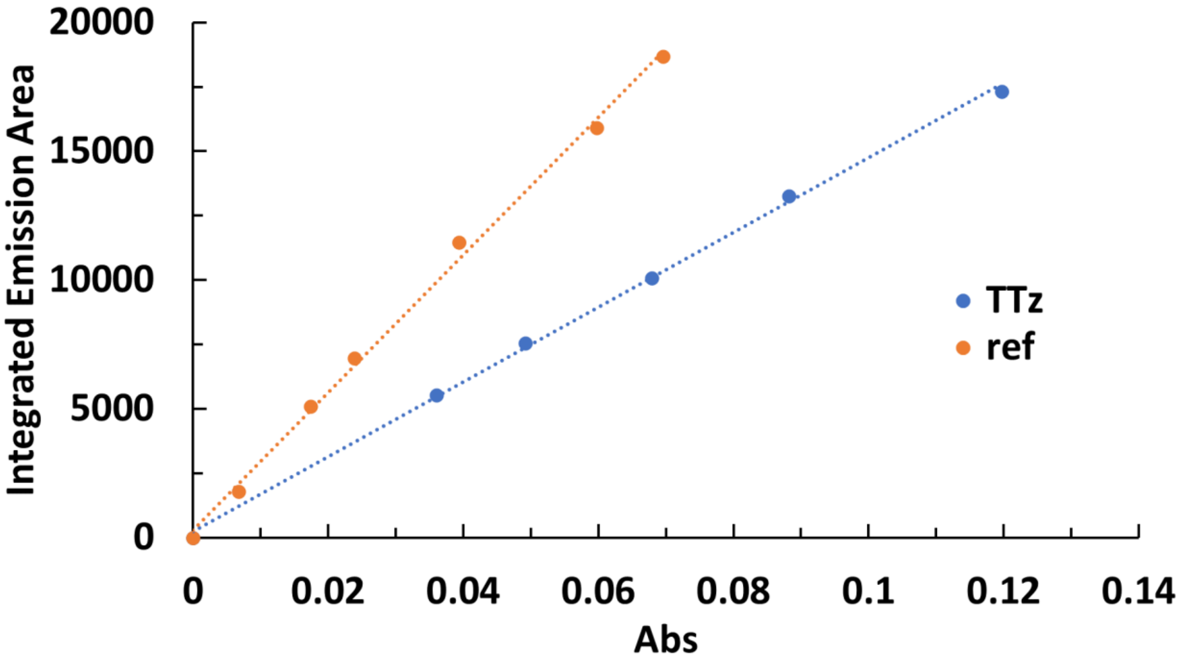

Figure S31: Example of relative fluorescence quantum yield with Ph_2N-TTz-NO_2 and ref perylene orange.

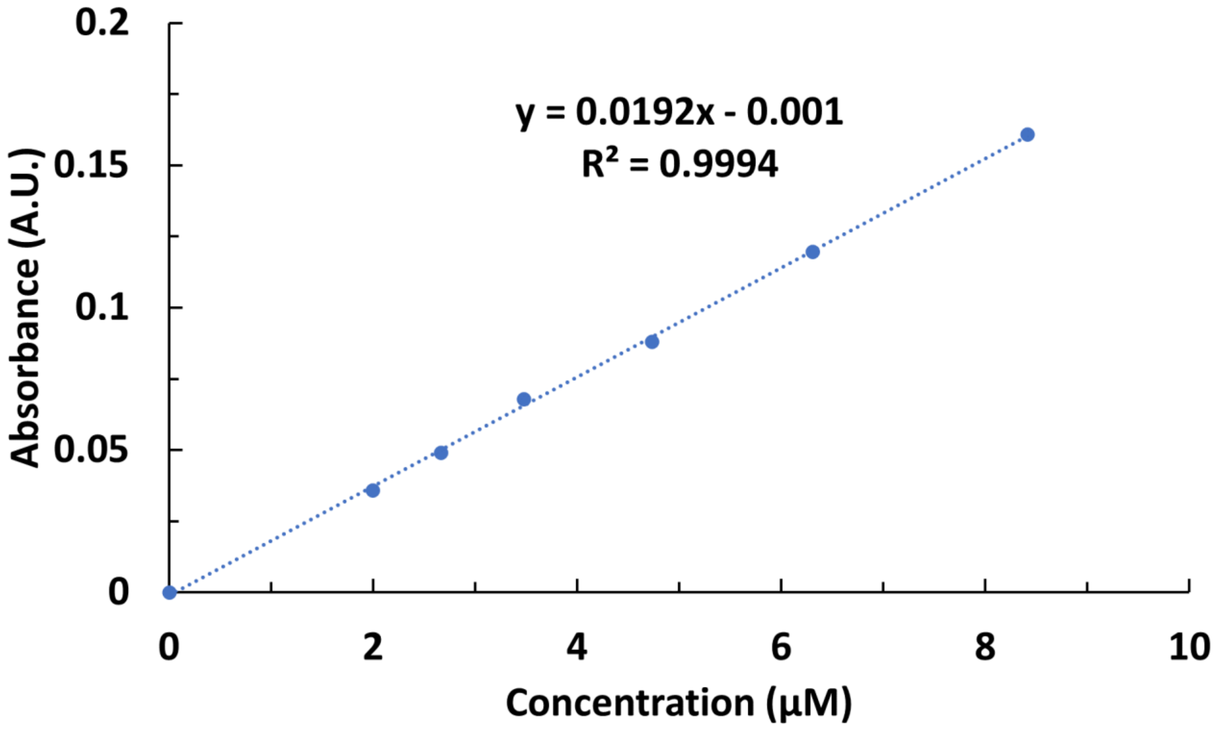

Figure S32: Example of molar absorptivity with Ph_2N-TTz-NO_2.

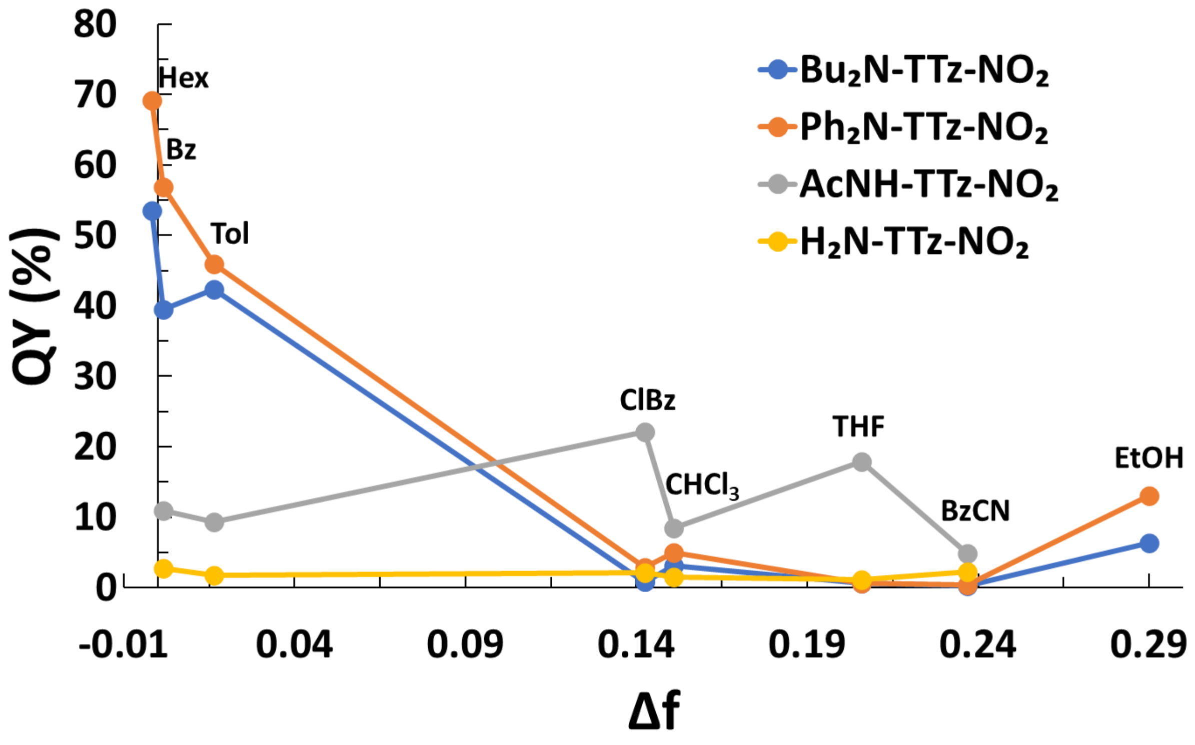

Figure S33: QY of asymmetric TTz's with increasing polarizability.

Section 3.4: Lippert-Mataga Plots

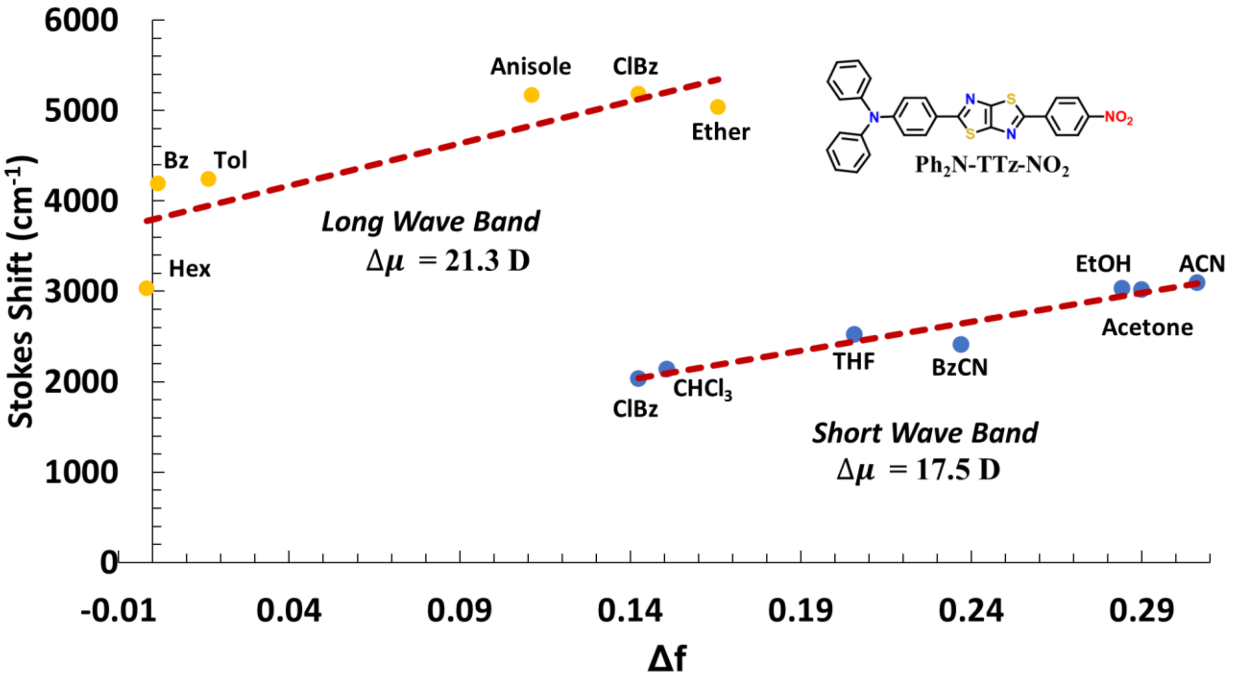

Figure S34: LM plot of Ph_2N-TTz-NO_2.

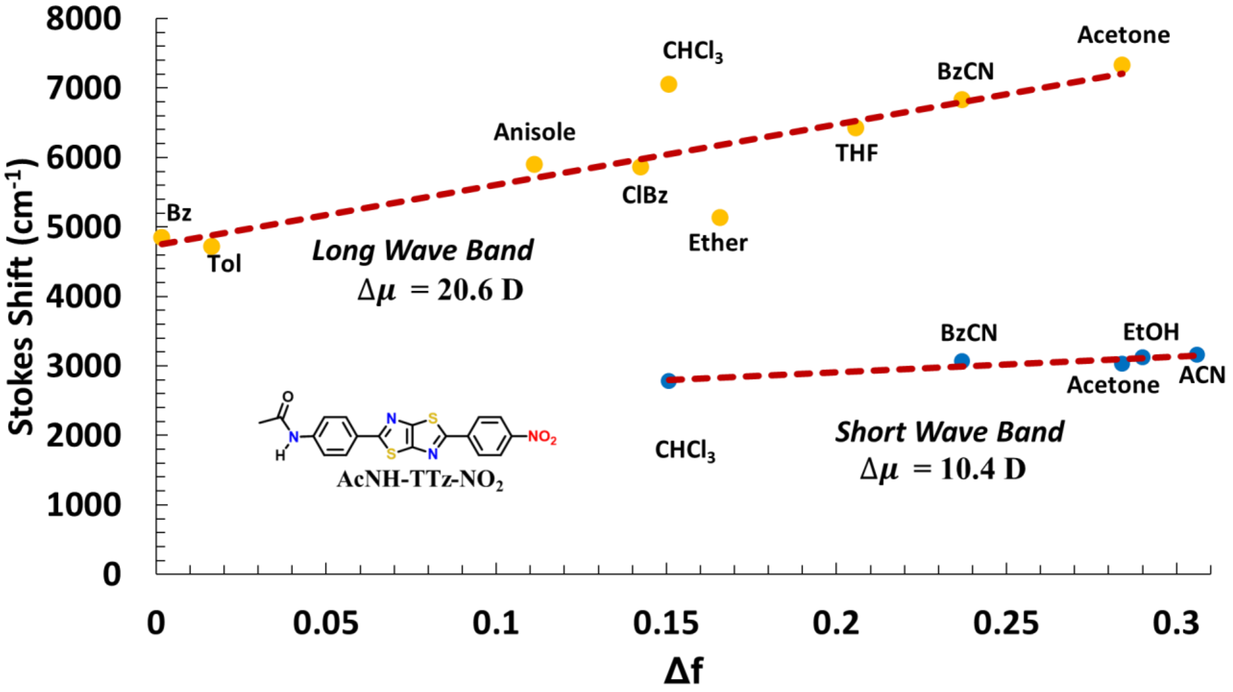

Figure S35: LM plot of AcNH-TTz-NO_2.

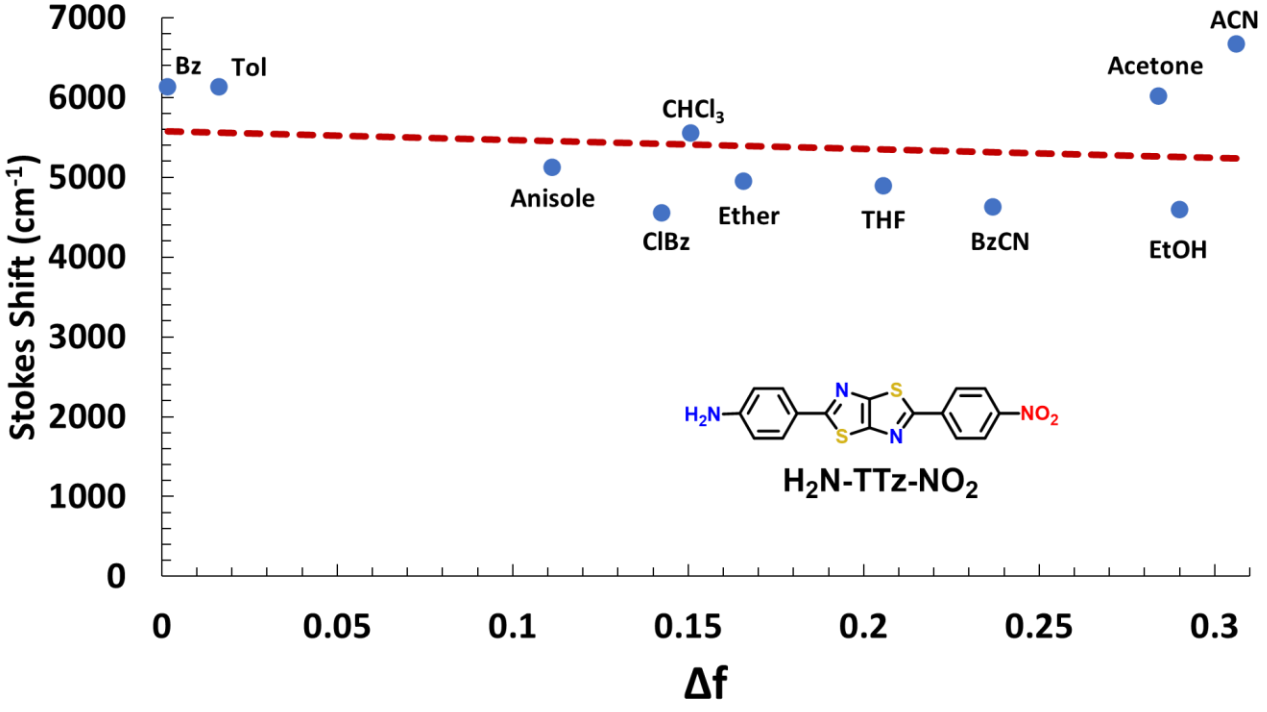

Figure S36: LM plot of H_2N-TTz-NO_2.

Section 3.5: Temperature Sensitivity

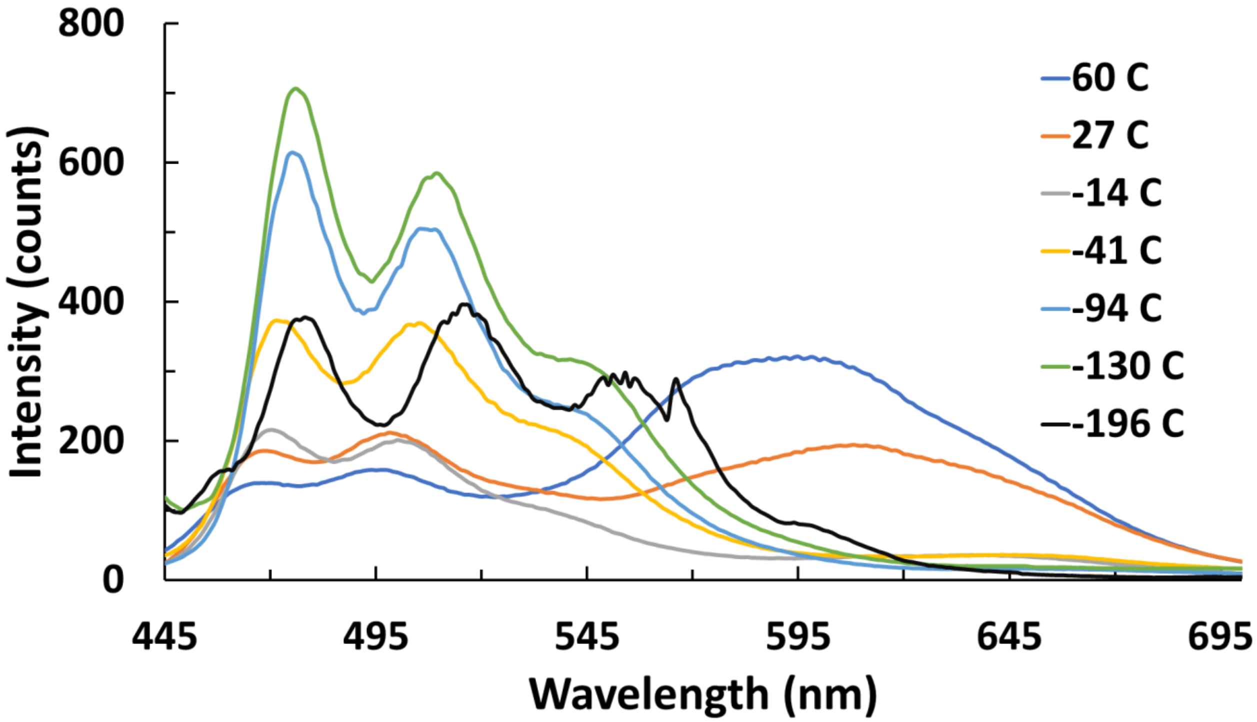

Figure S37: Emission wavelength of 80 µM Bu_2N-TTz-NO_2 in Me-THF at various temperatures.

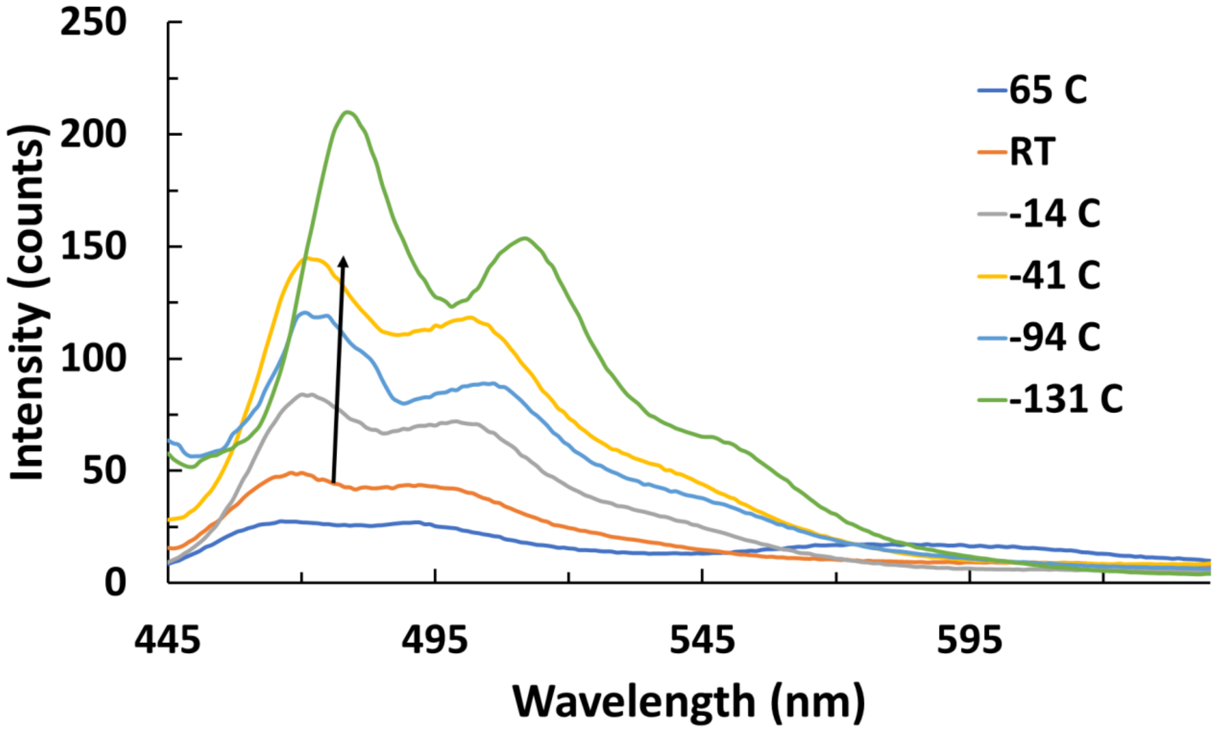

Figure S38: Emission wavelength of 10 µM Bu_2N-TTz-NO_2 in Me-THF at various temperatures.

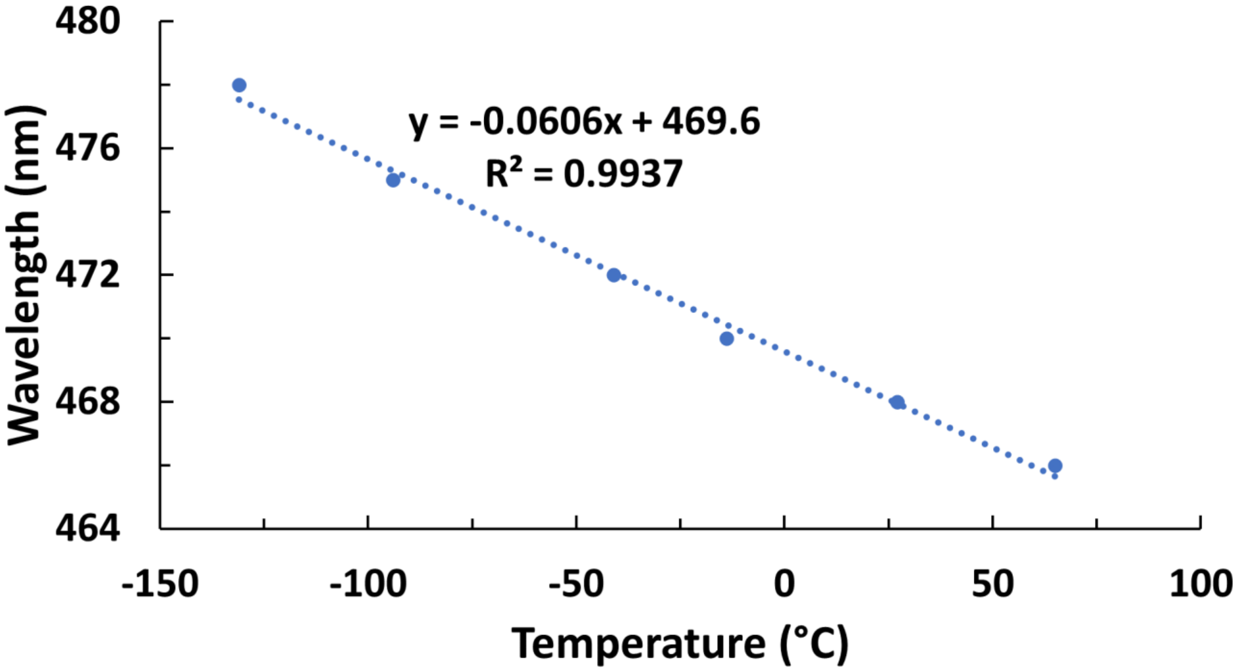

Figure S39: Emission wavelength of 10 µM Bu_2N-TTz-NO_2 in Me-THF.

Section 3.6: Polarizability

Table S2: Polarizability calculated from dielectric constant and refractive index of solvent. Constants obtained from sources at 20 or 25 °C.

Solvent	dielectric constant (ε)	index of refraction (η)	polarizability (Δf)
Hexane	1.88	1.3749	-0.0017
Benzene	2.27	1.5011	0.00164
Cl-Bz	5.6	1.5248	0.143
THF	7.26	1.4072	0.206
Bz-CN	26	1.5257	0.237
dioxane	2.2	1.416	0.0216
Ether	4.3	1.3524	0.166
Anisole	4.3	1.51791	0.111
EtOH	24.3	1.3571	0.290
Toluene	2.38	1.4864	0.0164
$CHCl_3$	4.81	1.4382	0.151
Acetone	20.7	1.3592	0.284
Acetonitrile	37.5	1.3421	0.306

Section 4: Computational Studies

For general vacuum optimizations, keywords: **# opt PBE1PBE/6-311+g(d,p) geom=save geom=connectivity** was used. Density functional theory (DFT) calculations were performed with Gaussian computational software using and PBE1PBE[4] density functionals and 6-311+G(d,p)[5] basis set.

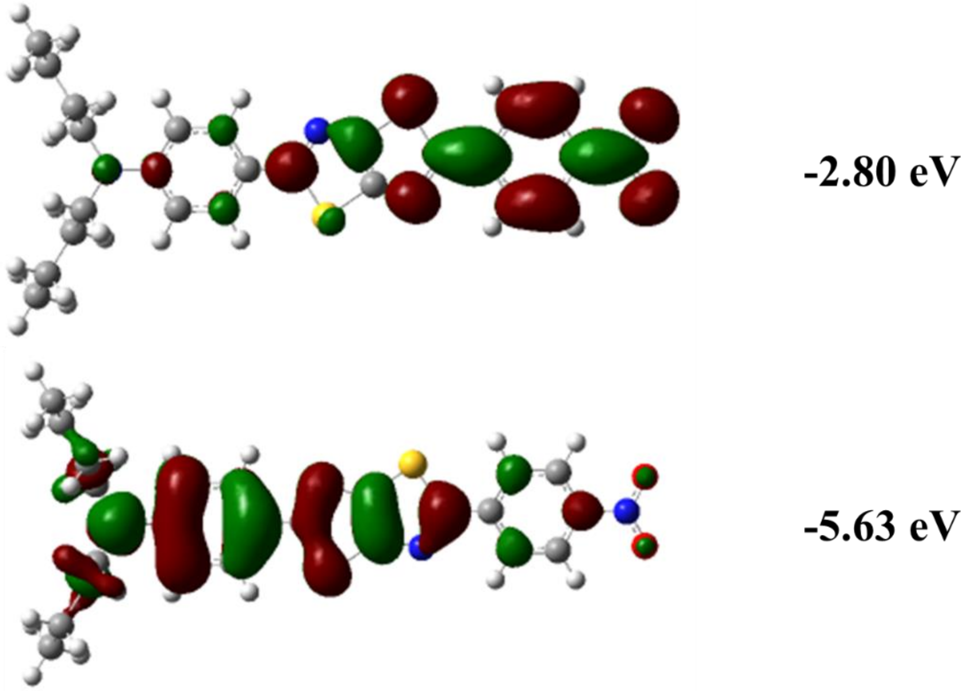

Figure S40: Ground state optimization with HOMO and LUMO of Bu_2N-TTz-NO_2 in vacuum.

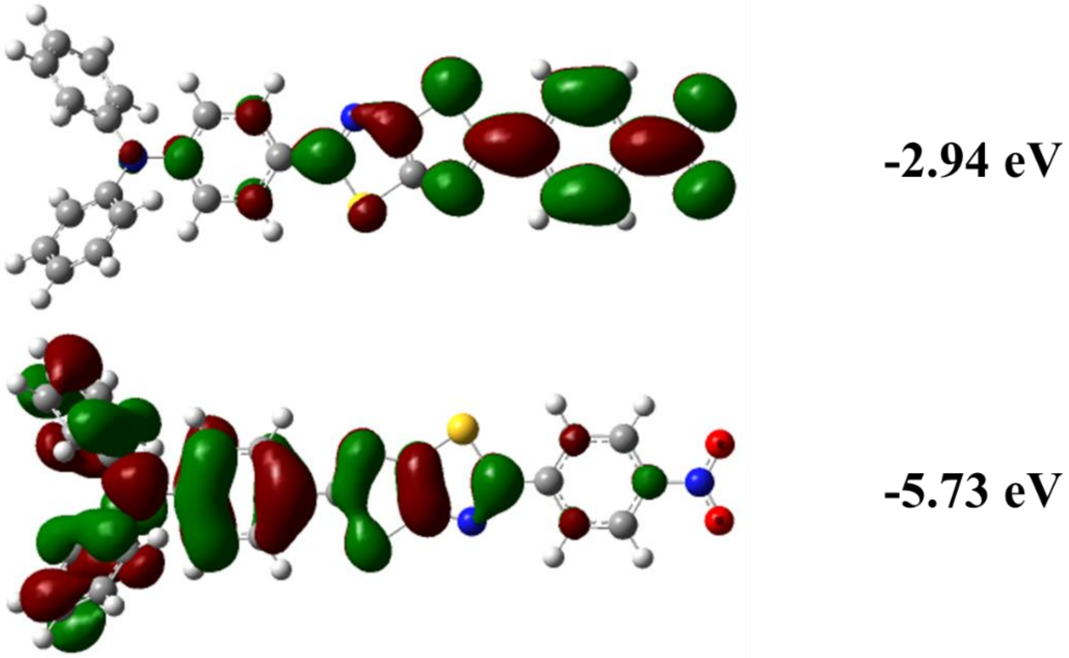

Figure S41: Ground state optimization with HOMO and LUMO of Ph_2N-TTz-NO_2 in vacuum.

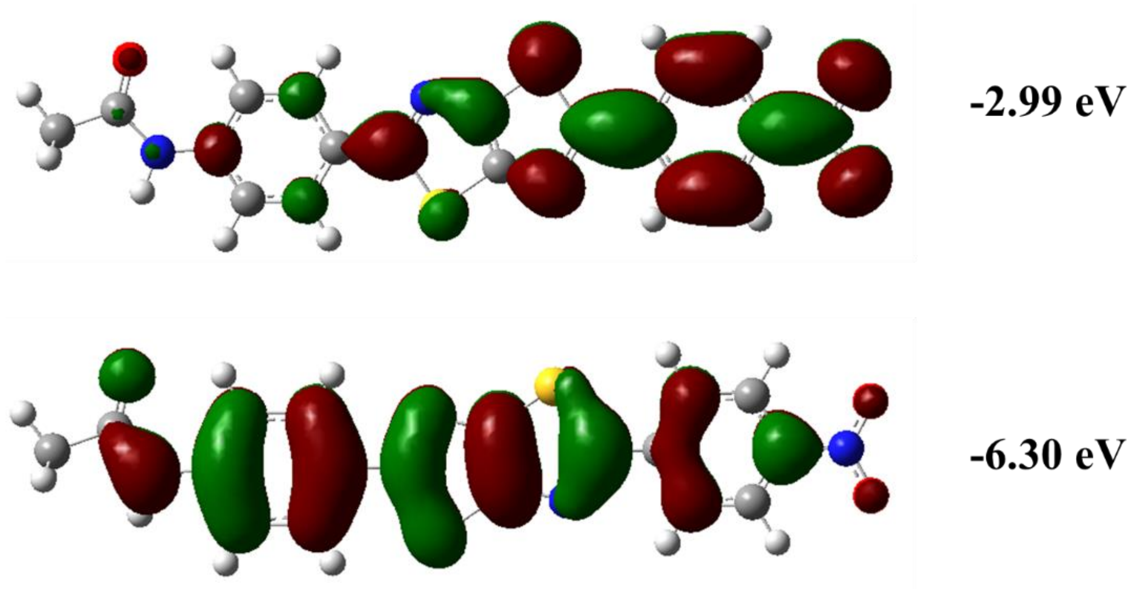

Figure S42: Ground state optimization with HOMO and LUMO of AcNH-TTz-NO_2 in vacuum.

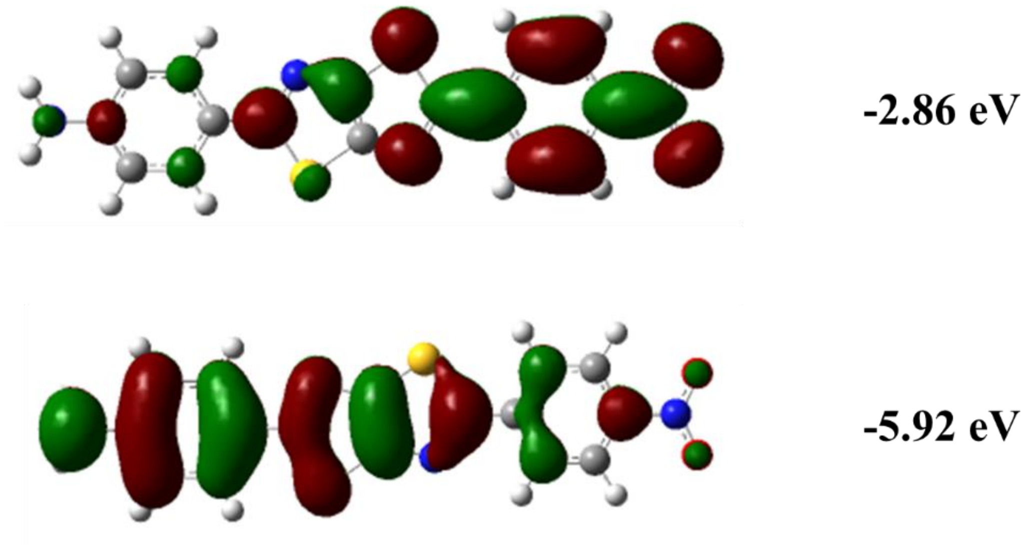

Figure S43: Ground state optimization with HOMO and LUMO of H_2N-TTz-NO_2 in vacuum.

To model the exited states, time-dependent calculations were also obtained. To model the SWB and LWB, Bu_2N-TTz-NO_2 was calculated in chlorobenzene due to observing emission from both excited states (**Figure 2**). Toluene was calculated to demonstrate exclusively LWB character and THF for SWB character. Geometry optimization would be computed with the IEFPCM solvation model and TDDFT computed [**# td=(nstates=10,root=1) pbe1pbe/6-311+g(d,p) scrf=(iefpcm,solvent=chlorobenzene) geom=connectivity**] to obtain a UV-Vis spectra and Frank-Condon excited state energies. The geometry optimized ground state TDDFT did not give an accurate model of the UV-Vis but it was found that twisting the amino-phenyl bond and optimizing while the amino-phenyl angle was frozen at 90° (modredundant), gave a more accurate absorbance spectra. This excited state would then be geometrically optimized to find the twisted excited state minima and the global excited state minima. Both were then computed again by TDDFT to determine the emission spectra.

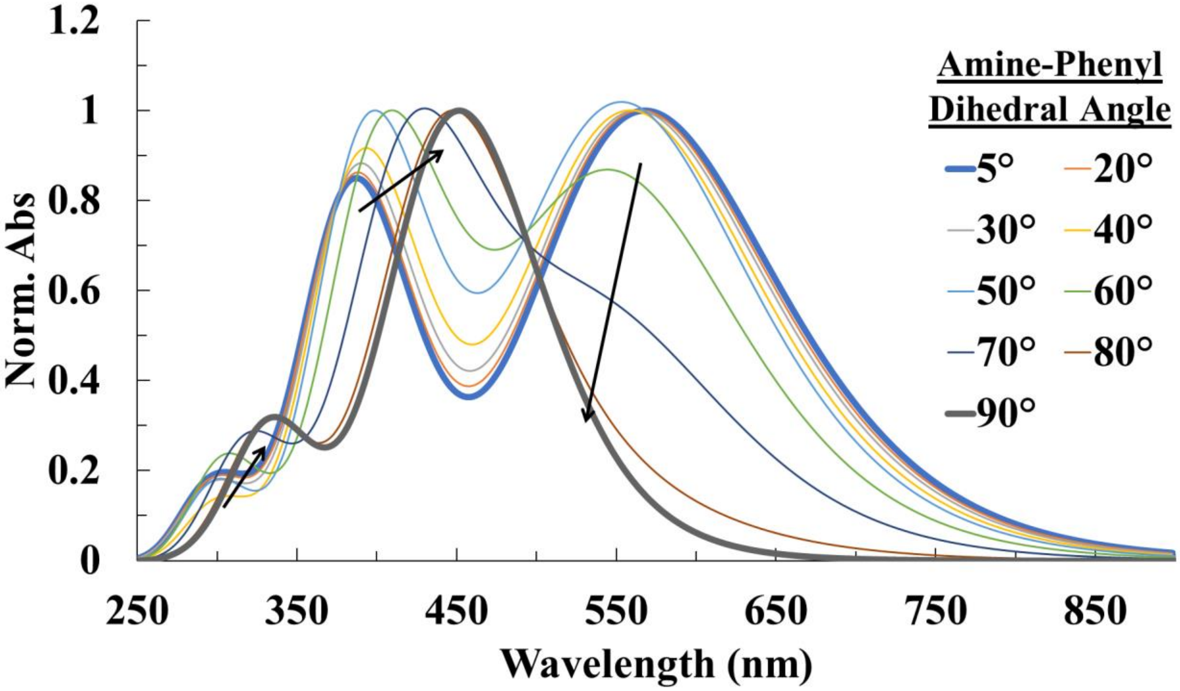

Figure S44: Absorbance spectra of Bu_2N-TTz-NO_2 in chlorobenzene while rotating the dihedral angle of the amino-phenyl bond.

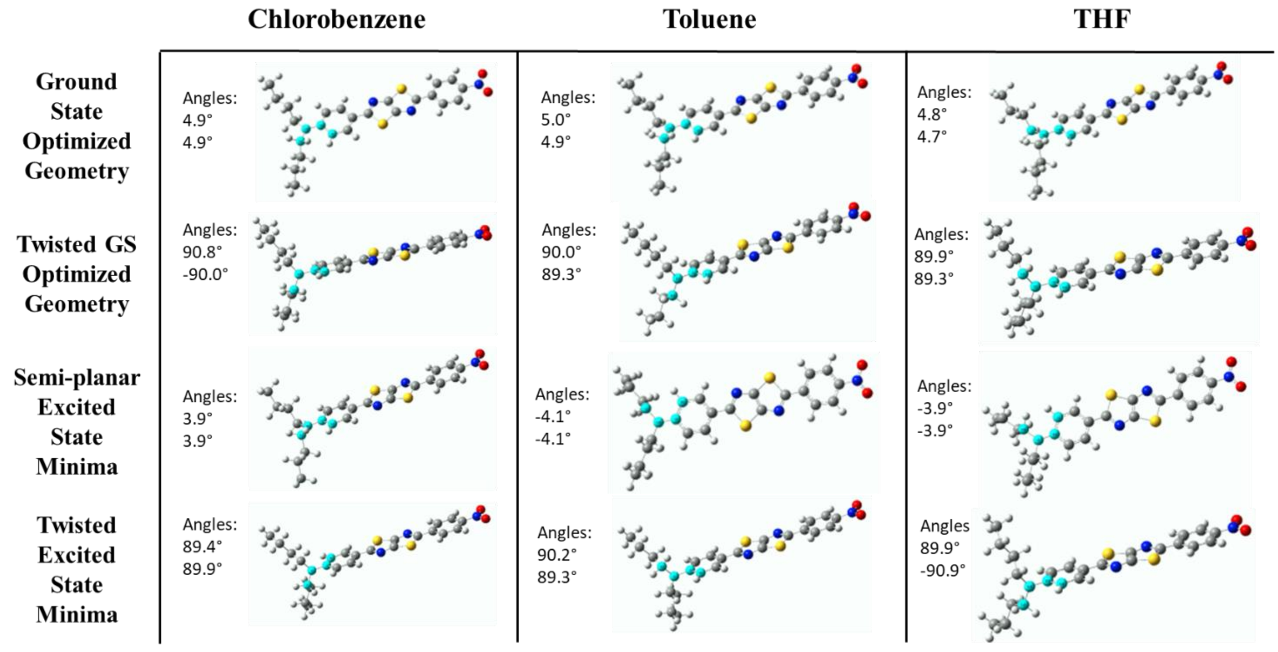

Figure S45: Computed angles for different states in various solvents. Twisted (orthogonal) angles were frozen.

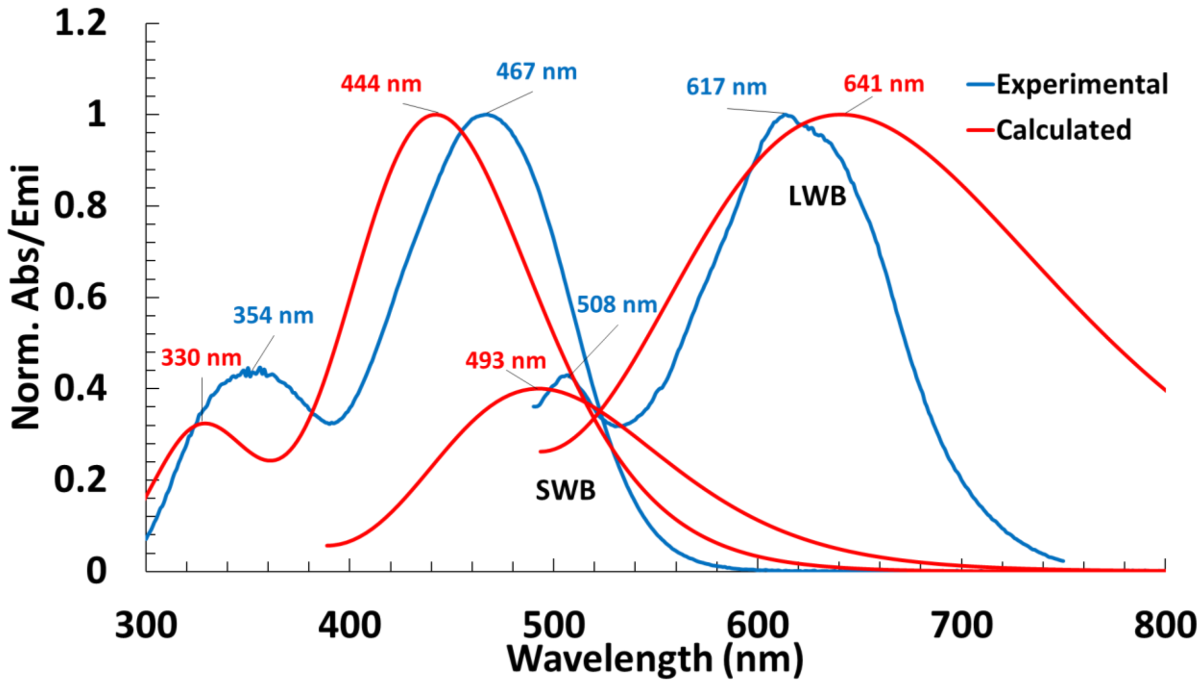

Figure S46: Experimental and calculated spectra of Bu_2N-TTz-NO_2 in chlorobenzene. The calculated band by the SWB is emission from the excited state minima of the twisted state and the calculated band by the LWB is the emission from the excited state minima of the semi-planar state.

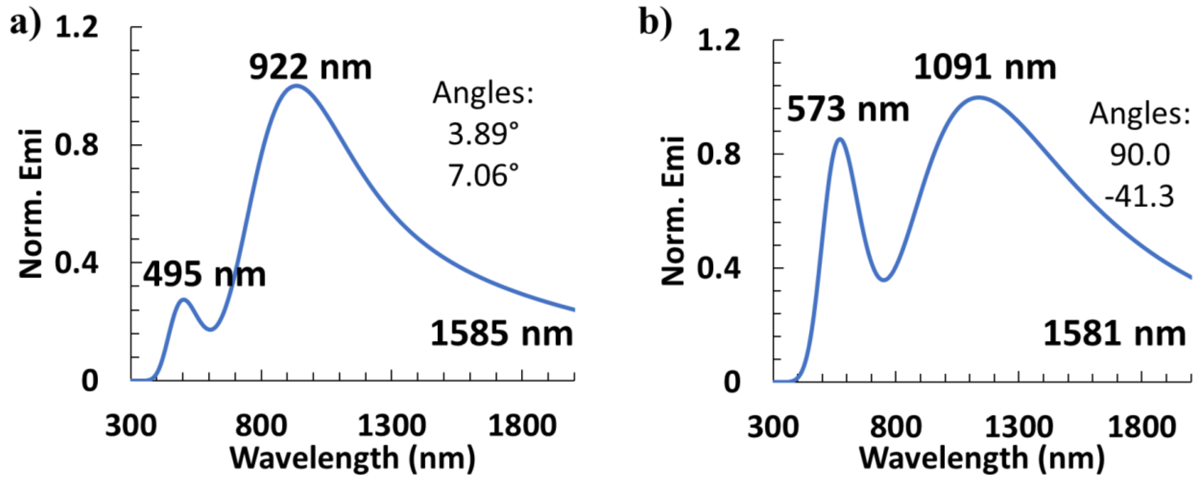

Figure S47: Triplet emission of the **a)** semi-planar and **b)** twisted excited states of Bu_2N-TTz-NO_2 in chlorobenzene (only one amine-phenyl bond was frozen).

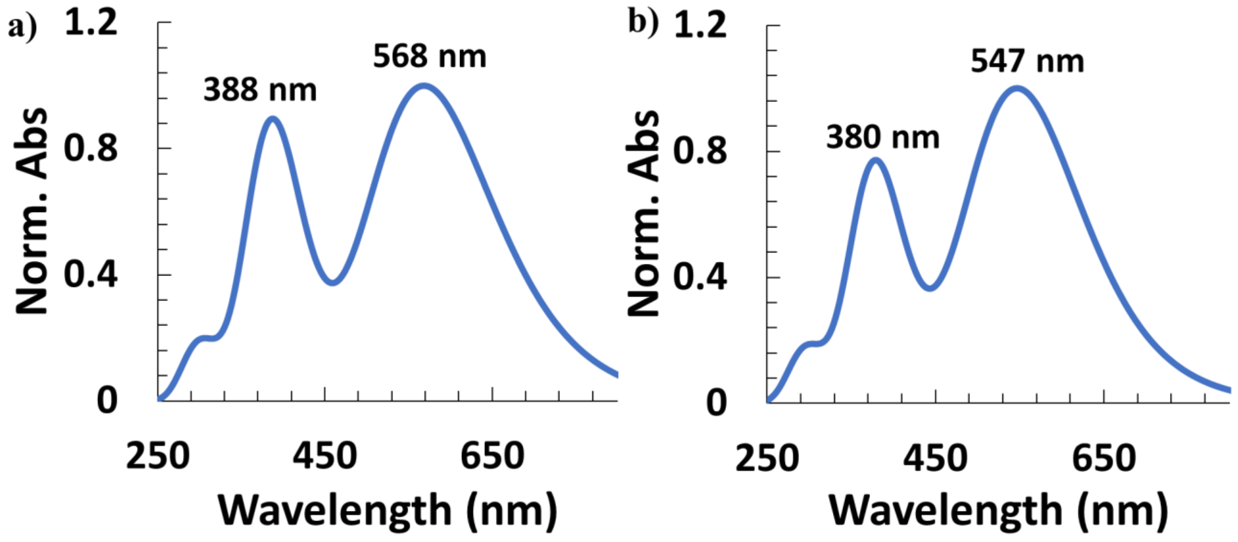

Figure S48: Absorbance of semi-planar geometry optimized Bu_2N-TTz-NO_2 in **a)** THF and **b)** toluene. These spectra are significantly different from the experimental results. The twisted ground state was explored due to these results.

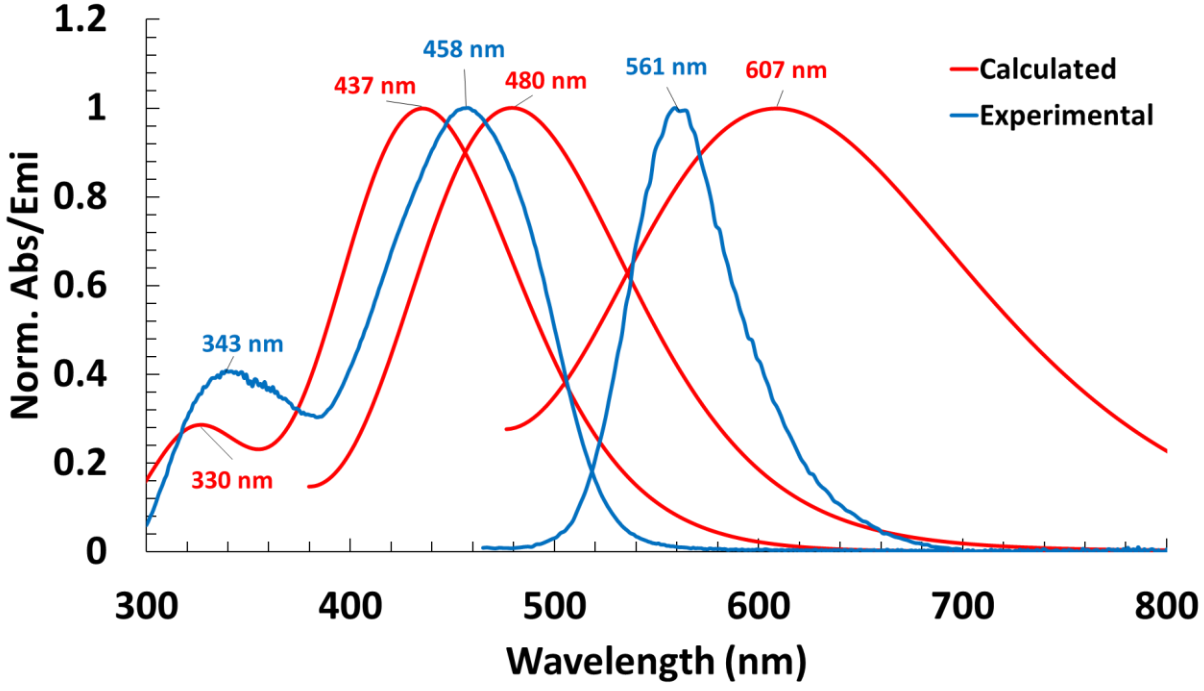

Figure S49: Experimental and calculated spectra of Bu_2N-TTz-NO_2 in toluene. The calculated band by the SWB is emission from the excited state minima of the twisted state and the calculated band by the LWB is the emission from the excited state minima of the semi-planar state.

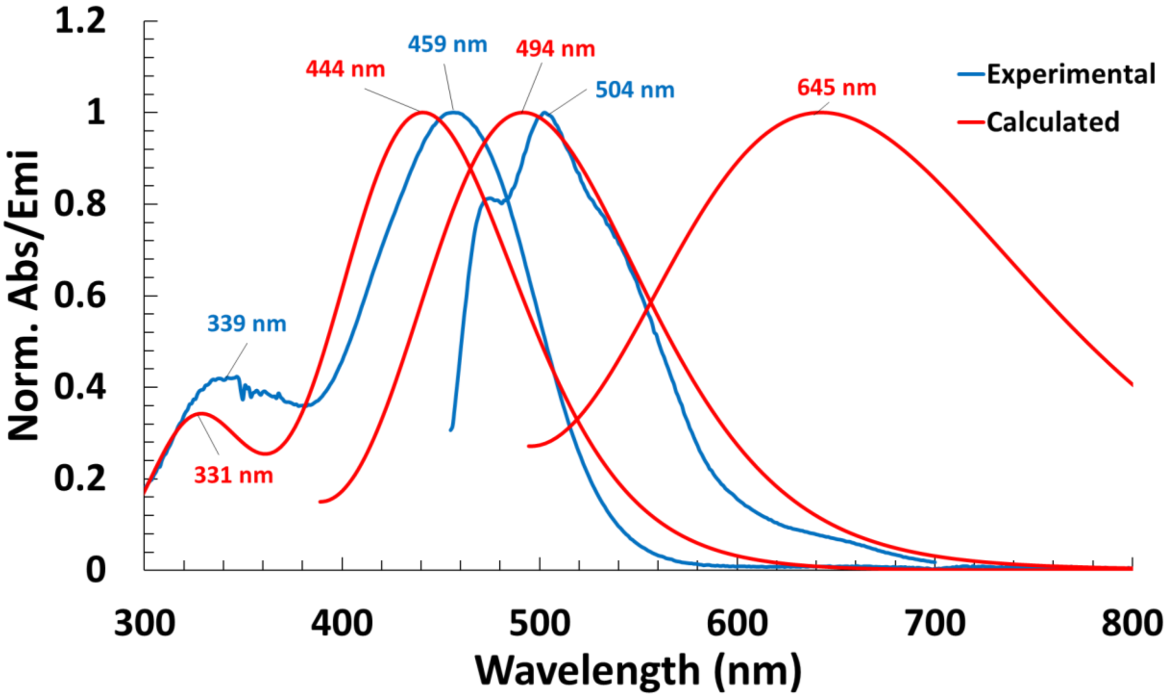

Figure S50: Experimental and calculated spectra of Bu_2N-TTz-NO_2 in THF. The calculated band by the SWB is emission from the excited state minima of the twisted state and the calculated band by the LWB is the emission from the excited state minima of the semi-planar state.

Section 5: Cyclic Voltammetry Figures and Tables

The Ph_2N-TTz-NO_2 shows a single electron reversible oxidation (E_{ox}) of the amino ($\Delta E = 74.8$ mV). This is common as the radical is stabilized by the phenyl groups and TTz core.[6] All other aTTzs in this work show poor reversible oxidation. Ph_2N-TTz-NO_2 and Bu_2N-TTz-NO_2 have similar E_{ox}, 0.50 and 0.45 V, respectively, while AcNH-TTz-NO_2 and H_2N-TTz-NO_2 had similar yet larger E_{ox}, 0.92 and 0.96 V, respectively. The larger E_{ox} of the acetamide is due to the stronger delocalization of the lone pair. There are additional peaks that appear as oxidation occurs with a larger shift in E_{ox} for H_2N-TTz-NO_2. This leads to the possibility of forming dimers or polymerization creating new substrates in solution. The E_{red} of the nitro group on all the a-TTzs are lower than other electronic bridges (thiophene, pyrrole, and furan).[7]

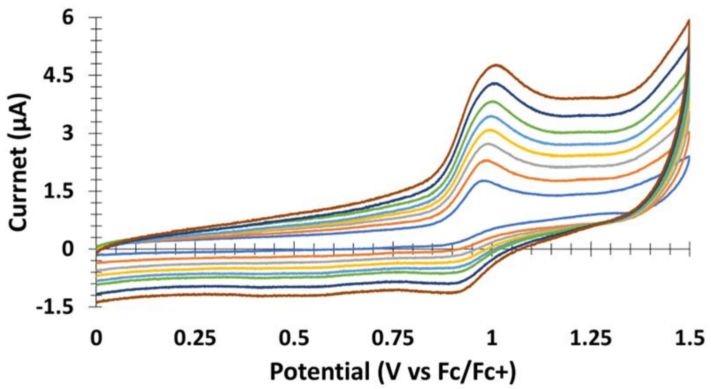

Figure S51: Oxidation sweep of Bu_2N-TTz-NO_2, 0 – 1.5 V (E_{ox} vs Fc/Fc^+). 50, 100, 150, 200, 250, 300, 400, 500 mV/s

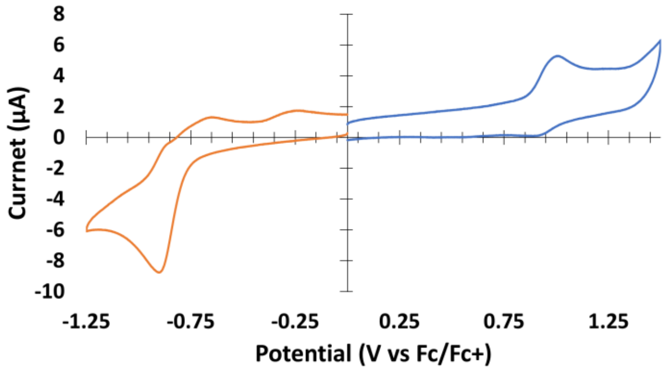

Figure S52: Oxidation and reduction sweep of Bu_2N-TTz-NO_2, 1.5 V – -1.25 V. 400 mV/s

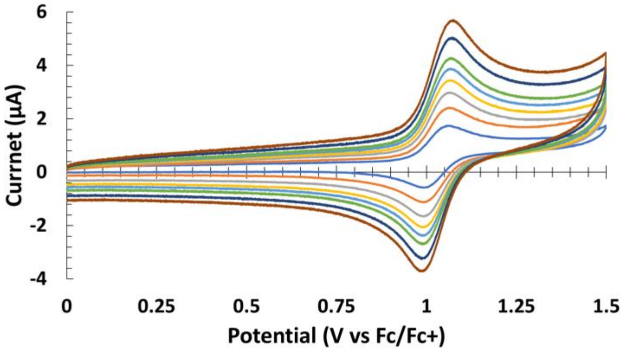

Figure S53: Oxidation sweep of Ph_2N-TTz-NO_2, 0 – 1.5 V (E_{ox} vs Fc). 50, 100, 150, 200, 250, 300, 400, 500 mV/s

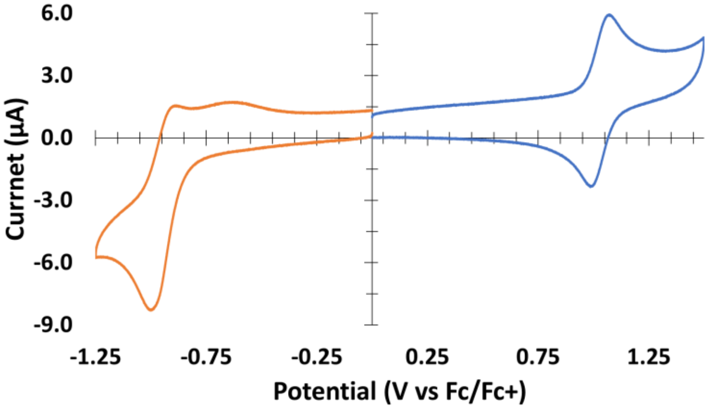

Figure S54: Oxidation and reduction sweep of Ph_2N-TTz-NO_2, 1.5 V to -1.25 V. 400 mV/s

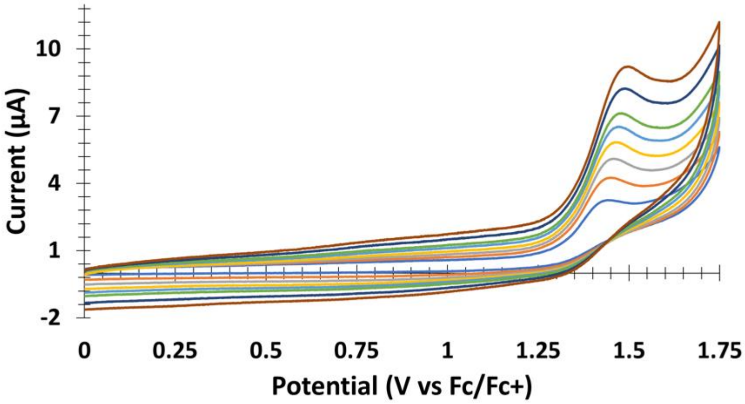

Figure S55: Oxidation sweep of AcNH-TTz-NO_2, 0 – 1.75 V (E_{ox} vs Fc). 50, 100, 150, 200, 250, 300, 400, 500 mV/s

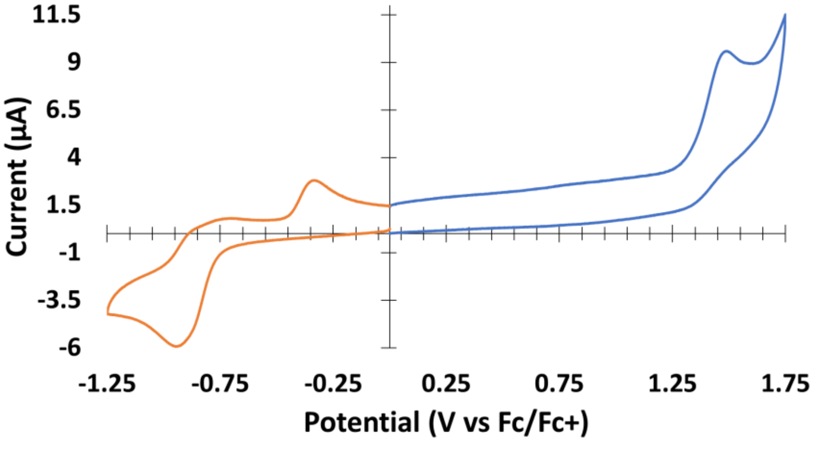

Figure S56: Oxidation and reduction sweep of AcNH-TTz-NO_2, 1.75 V – -1.25 V. 400 mV/s

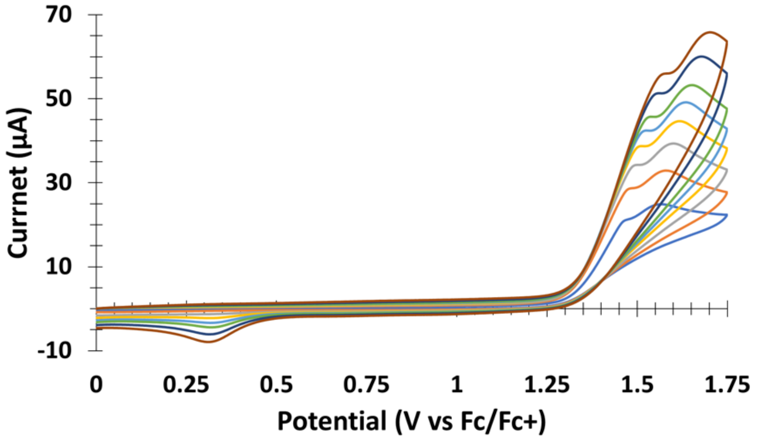

Figure S57: Oxidation sweep of H_2N-TTz-NO_2, 0 – 1.75 V (E_{ox} vs Fc). 50, 100, 150, 200, 250, 300, 400, 500 mV/s

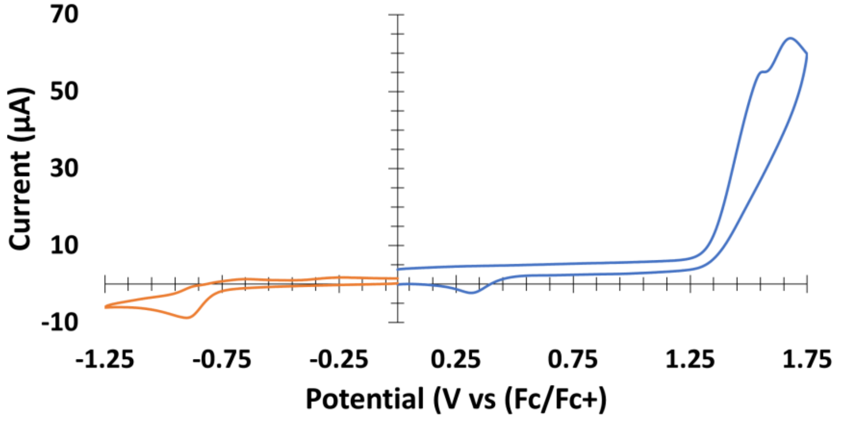

Figure S58: Oxidation and reduction sweep of H_2N-TTz-NO_2, 1.75 V – -1.25 V. 400 mV/s

Section 5.2: Diffusion Calculations

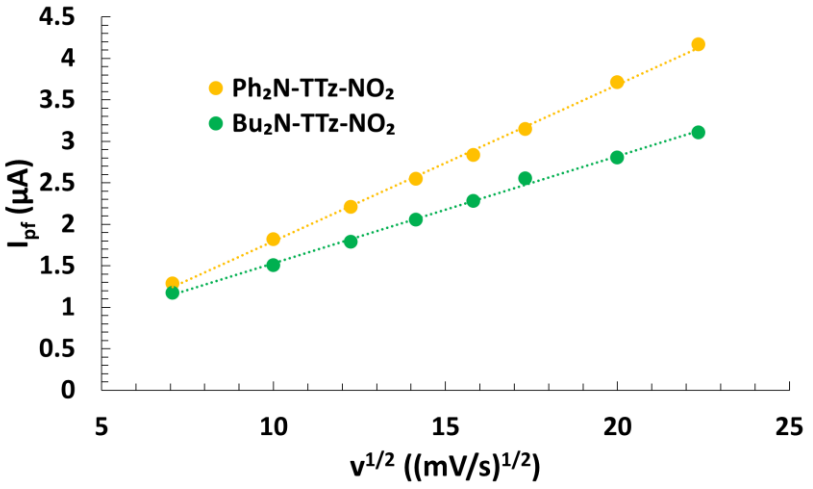

Figure S59: Randles-Sevcik of Bu_2N-TTz-NO_2 and Ph_2N-TTz-NO_2 for the oxidation step.

Table S3: Current of forward and reverse peak where applicable.

	Ph_2N-TTz-NO_2		*Bu_2N-TTz-NO_2*	*AcNH-TTz-NO_2*	*H_2N-TTz-NO_2*
**v** (mV/s)	$\mathbf{I_{pf}}$ (μA)	$\mathbf{I_{pr}}$ (μA)	$\mathbf{I_{pf}}$ (μA)	$\mathbf{I_{pf}}$ (μA)	$\mathbf{I_{pf}}$ (μA)
50	1.28	-1.12	1.17	2.48	24.15
100	1.82	-1.48	1.51	3.29	31.34
150	2.21	-1.93	1.79	3.93	37.86
200	2.55	-2.21	2.06	4.45	42.72
250	2.83	-2.50	2.28	4.96	46.82
300	3.15	-2.77	2.55	5.41	50.91
400	3.71	-3.20	2.81	6.06	57.22
500	4.17	-3.59	3.11	6.71	62.56

Table S4: Reversibility of Ph_2N-TTz-NO_2 from the ratio of I_{pf}/I_{pr}.

**v**	$\mathbf{I_{pf}/I_{pr}}$
50	1.14
100	1.23
150	1.14
200	1.15
250	1.14
300	1.14
400	1.16
500	1.16

Table S5. Redox potentials of a-TTz derivatives.

**a-TTz**	**D** ($cm^2\ s^{-1}$) x10^{-5}	$\mathbf{E_{ox}}$ vs Fc (V)	$\mathbf{E_{red}}$ vs Fc (V)	HOMO (eV)	LUMO (eV)
Ph_2N-TTz-NO_2	1.84	0.50	-1.40	-5.19	-3.29
Bu_2N-TTz-NO_2	0.24	0.45	-1.41	-5.14	-3.28
AcNH-TTz-NO_2	-	0.92	-1.47	-5.61	-3.22
H_2N-TTz-NO_2	-	0.96	-1.45	-5.65	-3.24

Section 6: Organic Vapor Polymer Sensor

Section 6.1: Solvent Vapor Sensing with Ph_2N-TTz-NO_2/SIS

Table S6: Max emission wavelength and intensity before, during, and after exposure to organic solvent vapors and the percent of initial fluorescence for samples exposed to saturated solvent vapors.

	λ_{emi} (nm)			Intensity (counts)			Percent of Initial Fluorescence	
**Solvent**	**Before**	**During**	**After**	**Before**	**During**	**After**	**During (%)**	**After (%)**
DCM	520	576	535	286.1	3.27	261.9	1.1	91.5
$CHCl_3$	520	---	520	499.9	0	470.7	0	94.2
Hex	520	500	498	434.7	118.1	74.4	27.2	17.1
THF	520	570	520	506.4	32.4	495.1	6.4	97.8
Ether	520	552	512	408.8	179.6	133.2	43.9	32.6
MeOH	520	538	524	400.3	255.2	361.9	63.8	90.4
EtOH	520	542	522	515.4	354.1	481.1	68.7	93.3
Tol	520	540	526	496.3	437.1	503.3	88.1	101.4
TEA	520	---	523	321.3	32.6	188.2	10.0	58.6
DEA	520	546	521	507.6	108.9	422.7	21.4	83.3

The ppm was derived from the vapor pressure of the solvent calculated from *The Yaws Handbook of Vapor Pressure* and the ppm and pressure of the solvent below the saturated vapor pressure was calculated from the ideal gas law.

$$PV = nRT \qquad \text{Eq. 1}$$

Where the volume (V) was measured using water displacement 23.3 mL, the gas constant (R) 0.821 L atm / K mol, the temperature (T) 293.15 K, and the mols, n, from assuming all the liquid enters the vapor phase.

Table S7: Calculated pressure values for saturated and % saturated vapor samples (using 1 µL of solvent) and lower limit* using 4L flask with 5 min solvent exposure.

**Solvent**	**Std. Vapor Pressure (atm)**	**Saturated Solvent (µmol)**	**Saturated (ppm) (µmol/mol)**	**Solvent with 1 µL (µmol)**	**1 µL (ppm) (µmol/mol)**	**Pressure (atm)**	**% of Sat. Vapor Pressure**
DCM	0.465	449	465000	15.7	16200	0.016	3.48
$CHCl_3$	0.201	195	201000	12.5	12900	0.013	6.41
Hex	0.156	150	156000	7.60	7860	0.008	5.05
THF	0.167	161	167000	12.3	12700	0.013	7.63
THF*	0.167	161	167000	12.3	66	0.00007	0.04
Ether	0.576	557	576000	9.62	9950	0.010	1.73
MeOH	0.117	113	117000	24.7	25600	0.026	21.8
EtOH	0.059	57	59000	17.1	17700	0.018	30.2
Tol	0.0294	28	29000	9.41	9740	0.010	33.1
TEA	0.044	43	44000	7.70	7970	0.008	18.1
DEA	0.206	199	206000	9.67	10000	0.010	4.86

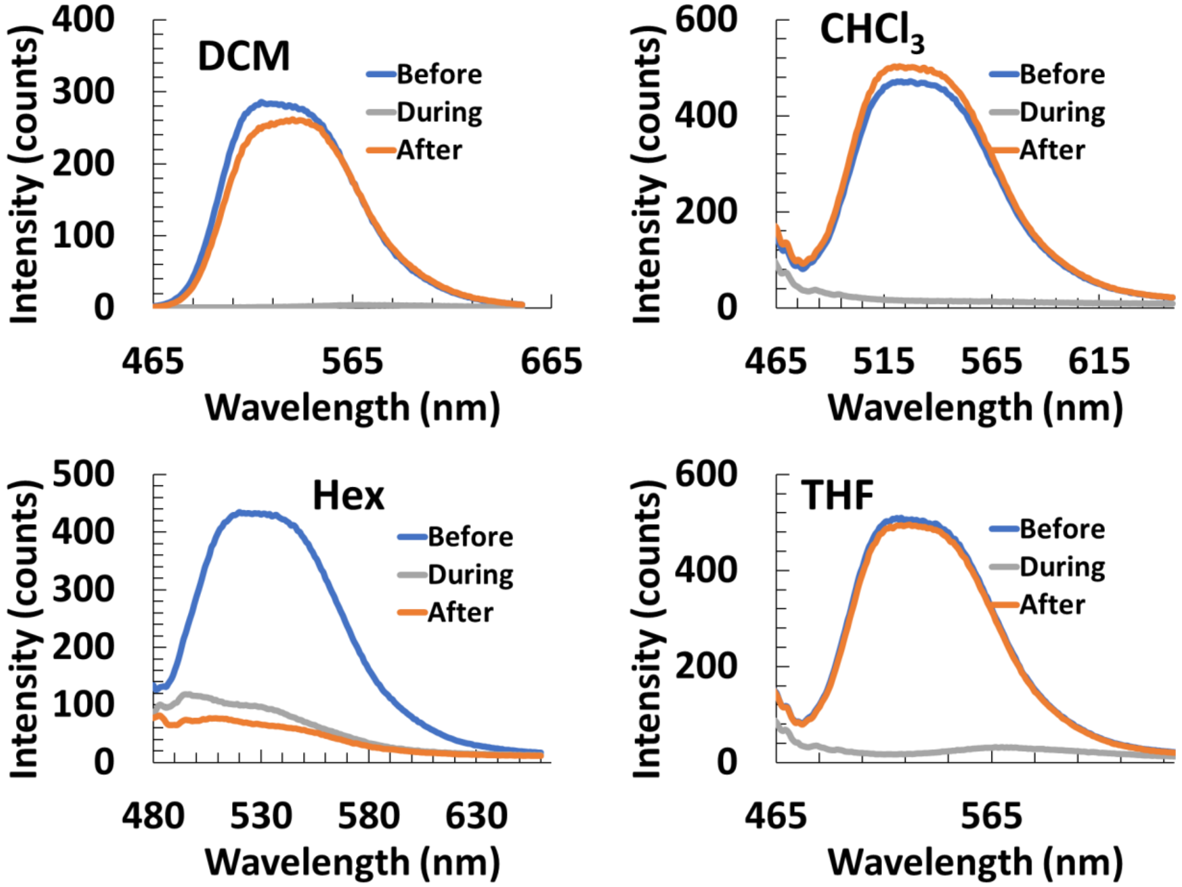
DCM
Before
During
After
Intensity (counts)
Wavelength (nm)
CHCl3
Hex
THF

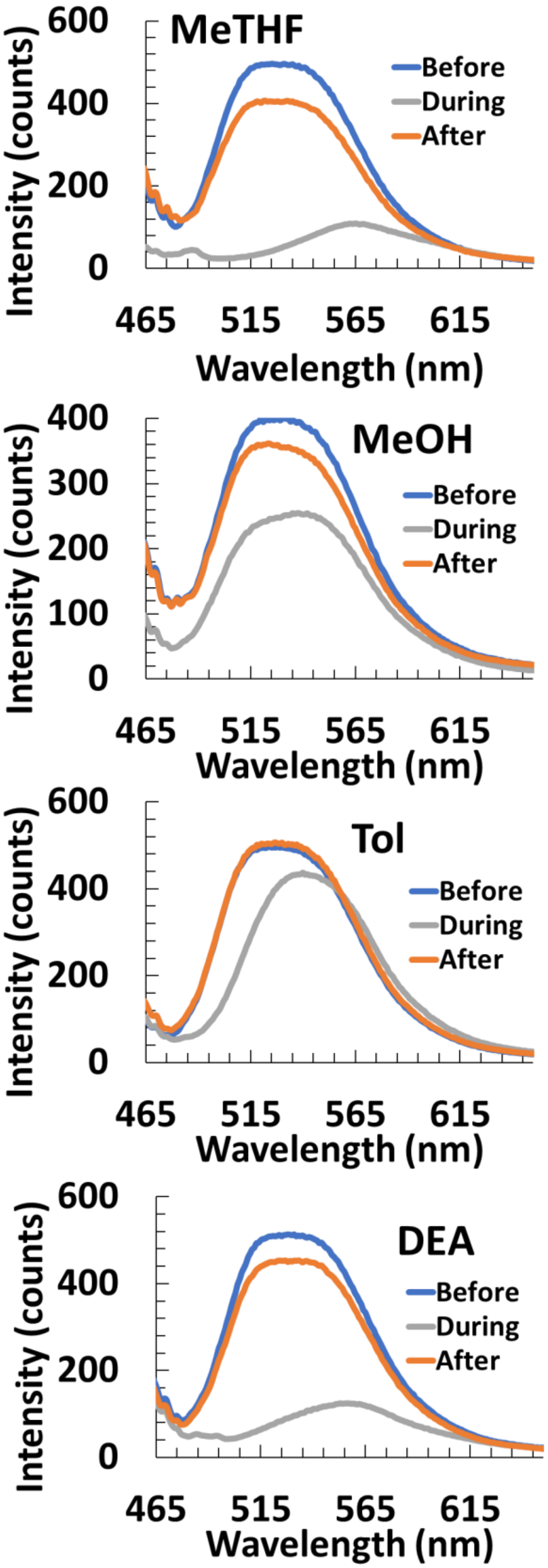

Figure S60: Vapor sensing with several solvents (saturated vapor pressure) using Ph_2N-TTz-NO_2 in SIS.

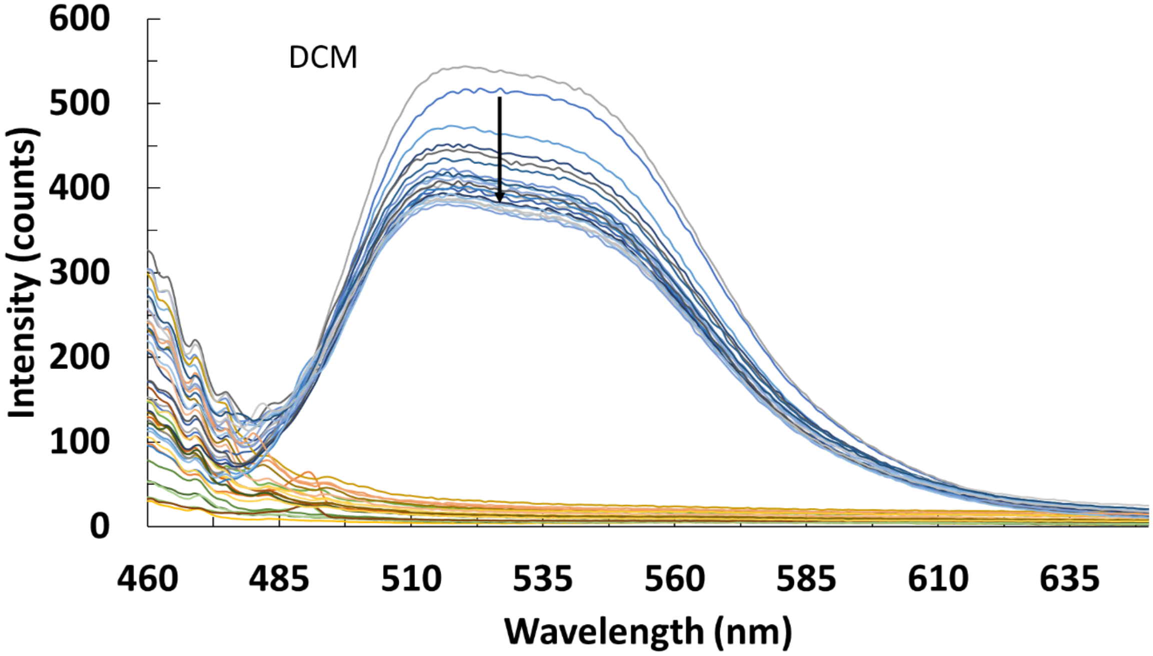

Figure S70: Emission spectra of spin coated Ph_2N-TTz-NO_2 in SIS polymer sequentially exposed to saturated DCM solvent vapors 20 times. (λ_{exi} = 445 nm)

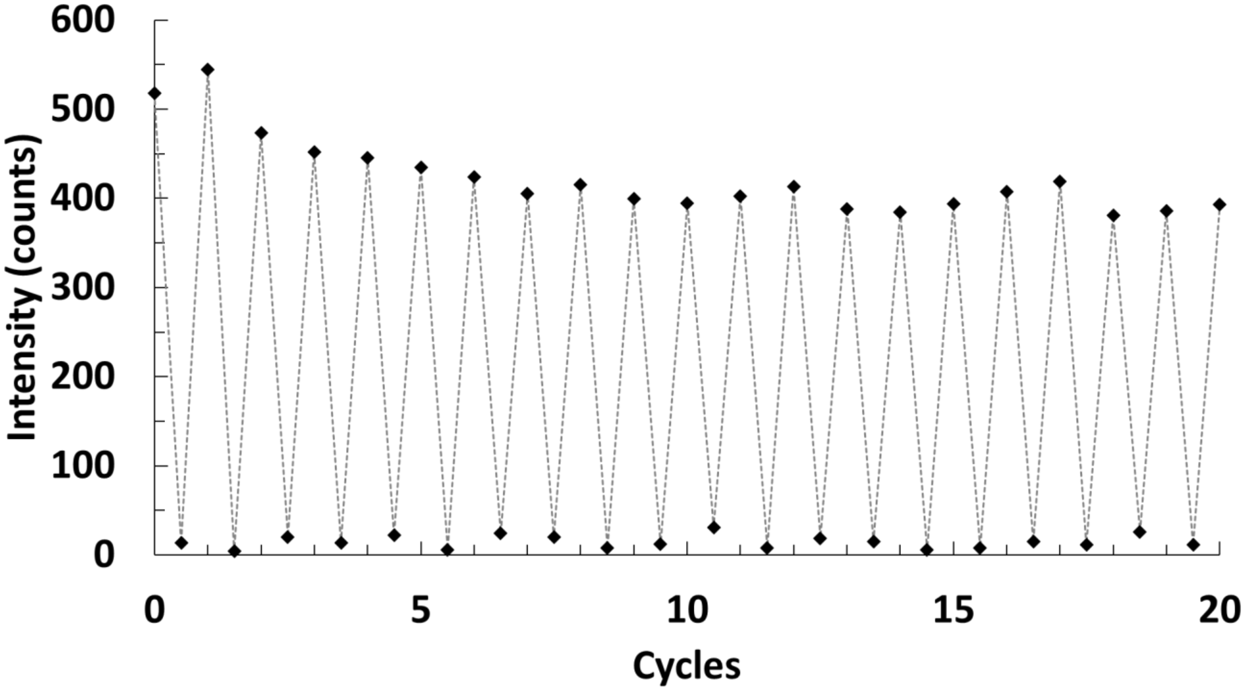

Figure S71: Cycling plot of the max emission (λ_{emi} = 520 nm) during each exposure of DCM and after.

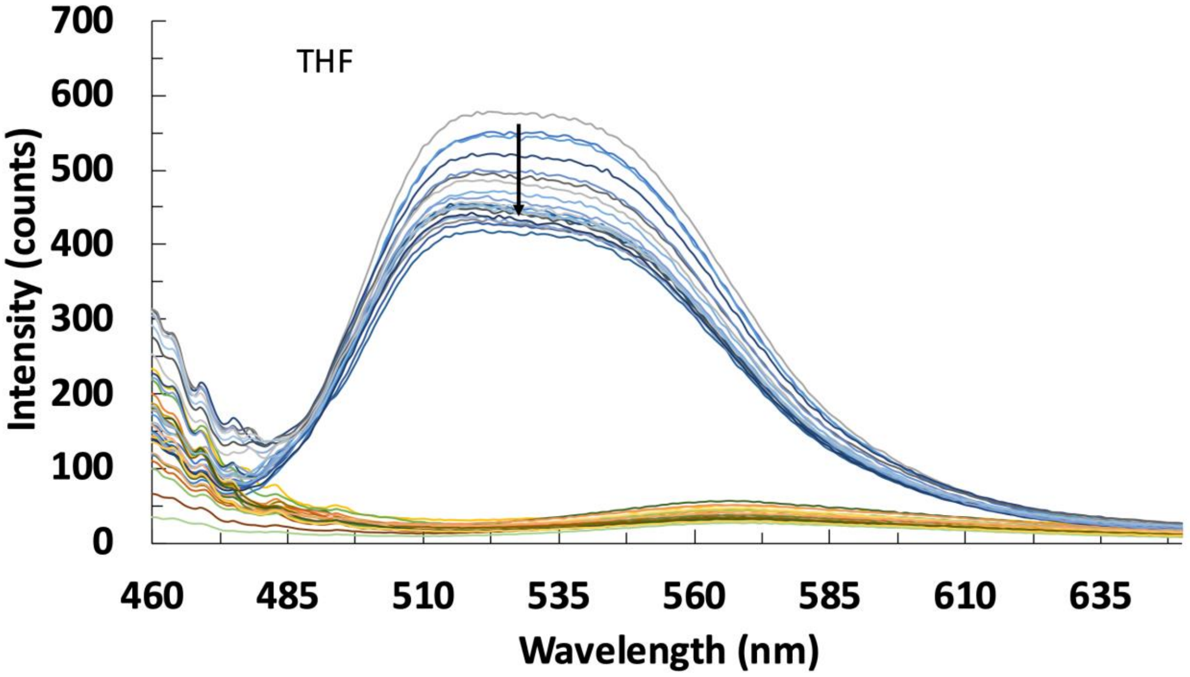

Figure S72: Emission spectra of spin coated Ph_2N-TTz-NO_2 in SIS polymer subsequently exposed to saturated THF vapors 20 times. (λ_{exi} = 445 nm)

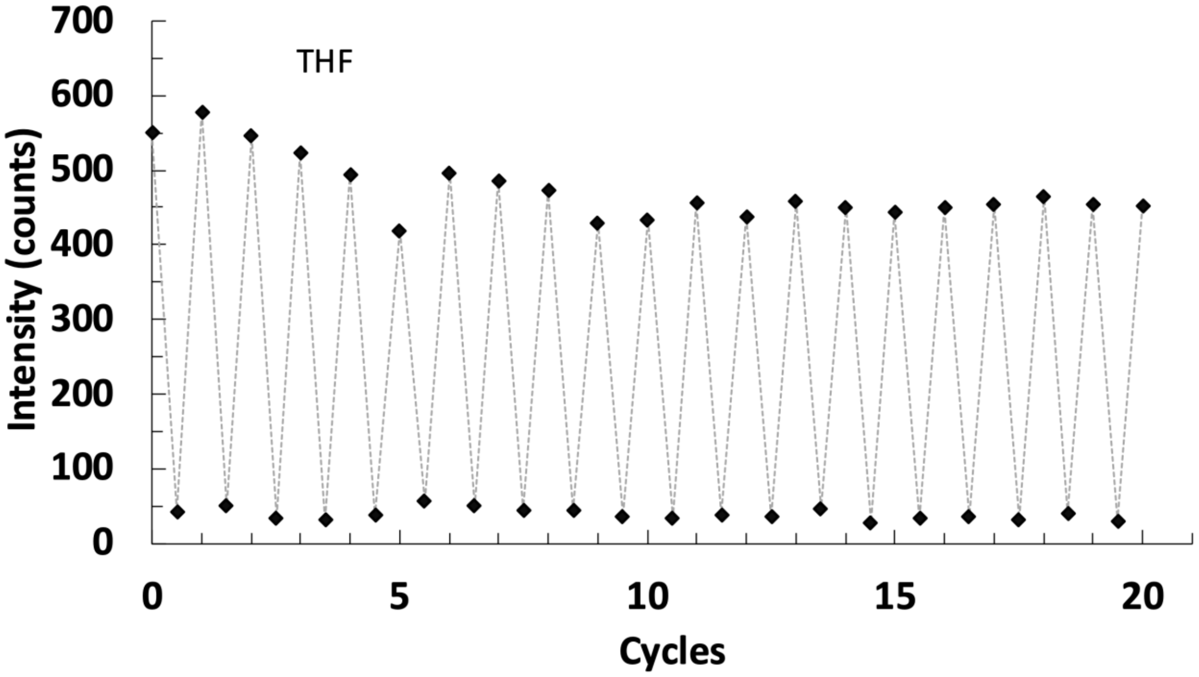

Figure S73: Cycling plot of the max emission (λ_{emi} = 520 nm) during each exposure of THF and after.

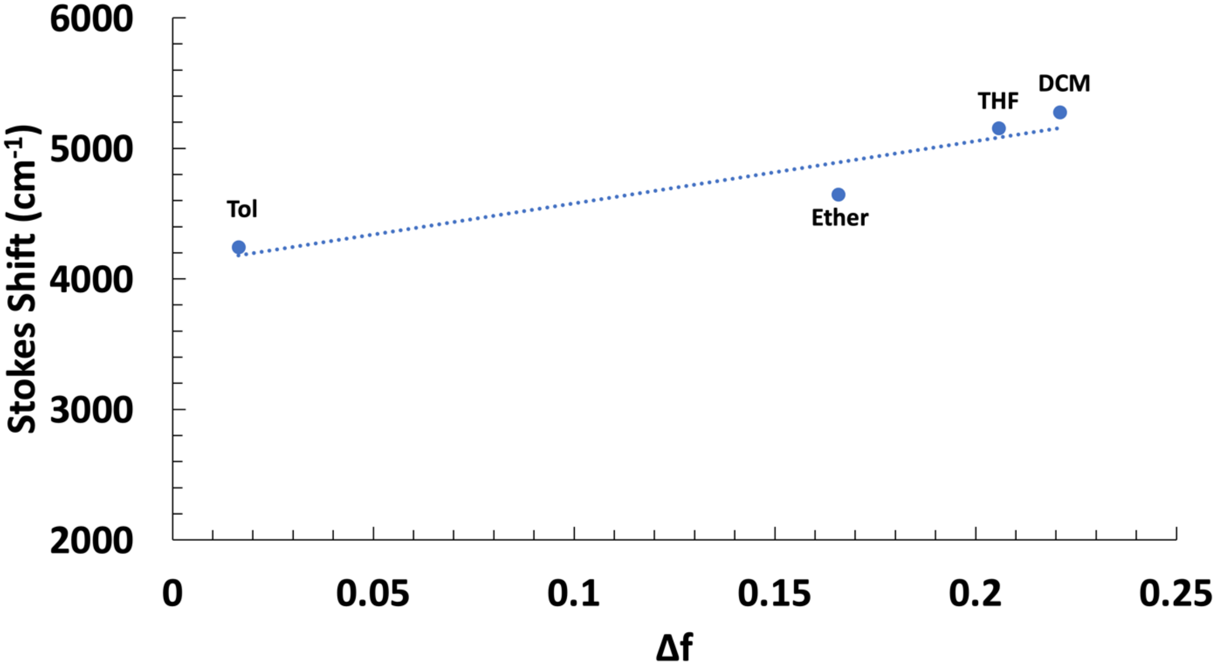

Figure S74: Lippert-Mataga plot of the Ph_2N-TTz-NO_2 doped SIS polymer when exposed to saturated organic solvents where a shifted peak was observed.

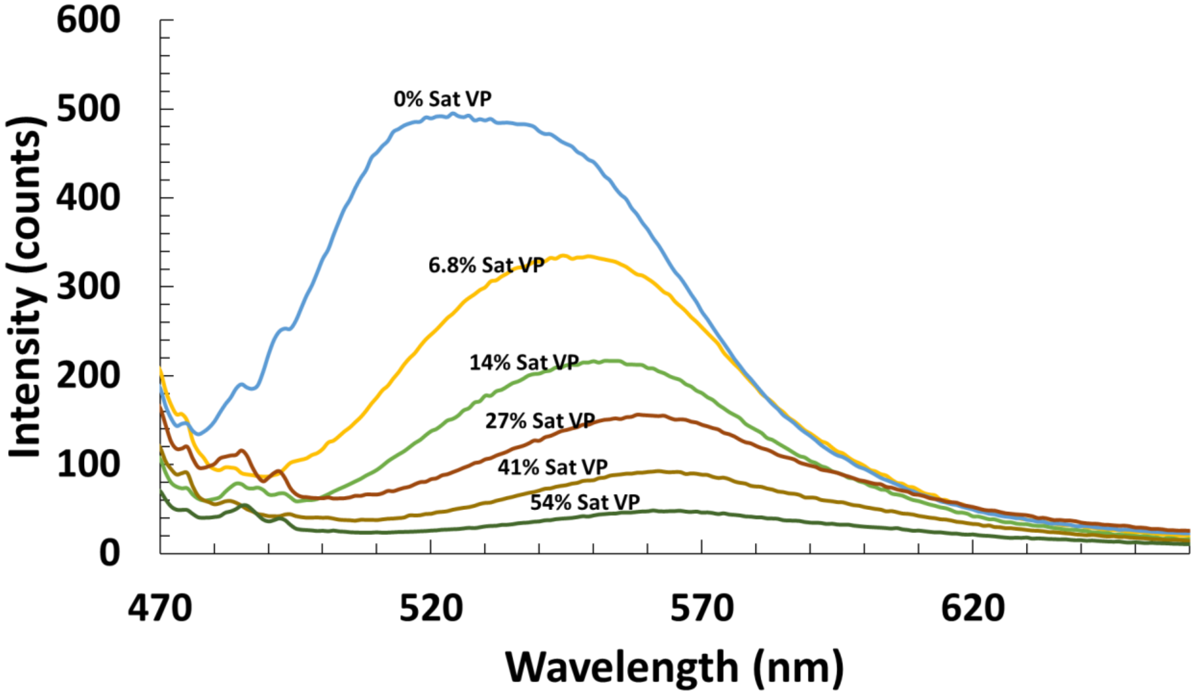

Figure S75: Fluorescence emission variations of Ph_2N-TTz-NO_2 / SIS thin films when exposed to various % saturated vapor pressure of THF.

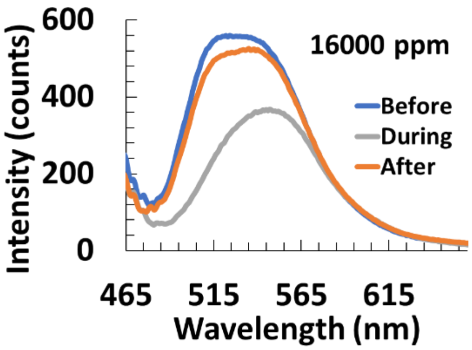

Figure S76: Organic solvent vapor sensing of DCM with Ph_2N-TTz-NO_2 in SIS.

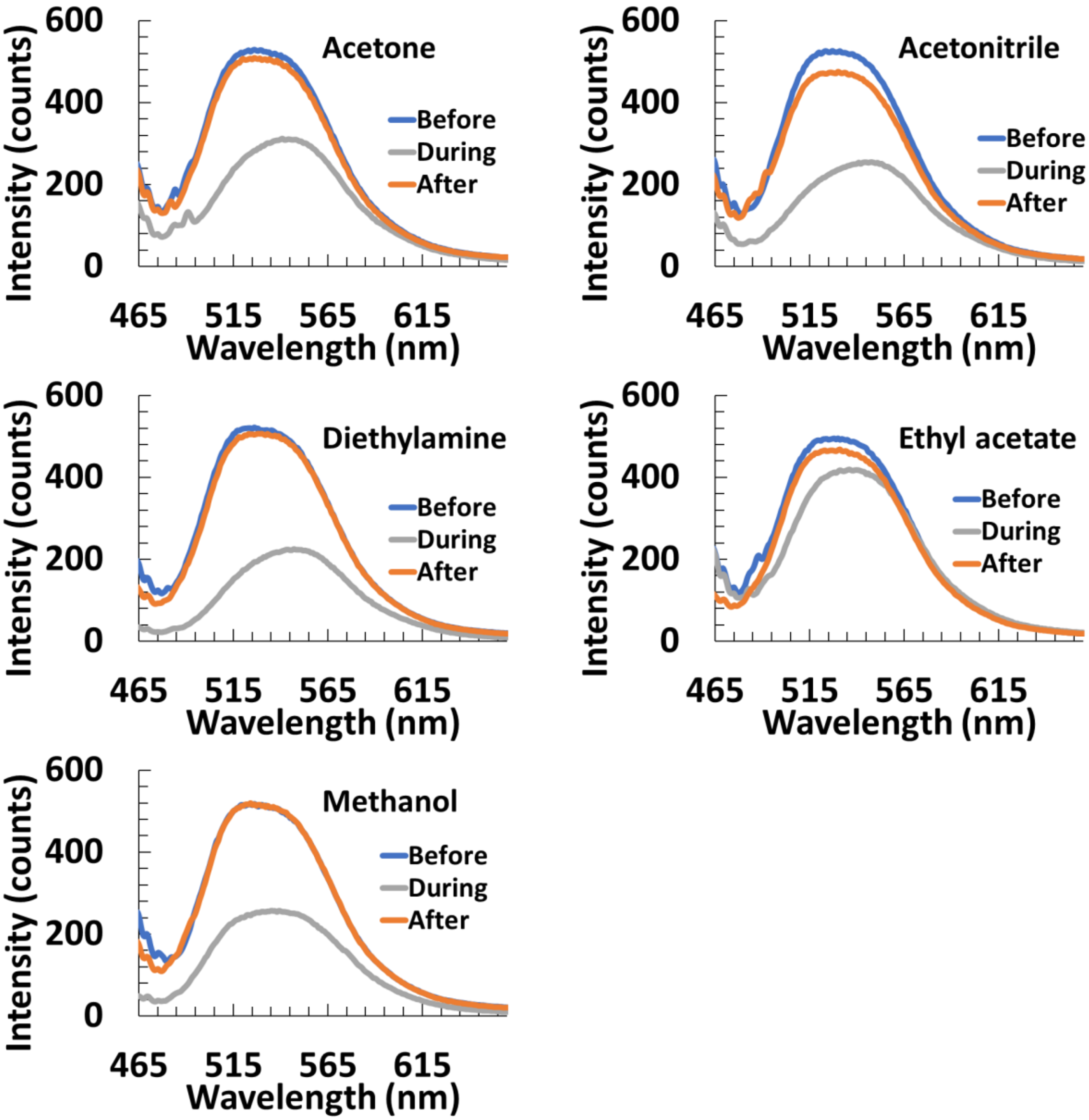

Figure S77: 1 μL vapor sensing of several solvents with Ph_2N-TTz-NO_2 in SIS.

Section 6.2: Solvent Vapor Sensing with Ph_2N-TTz-Py/SIS

Table S8: Max emission wavelength and intensity before, during, and after exposure to organic solvent vapors and the percent of initial fluorescence. The ppm was given derived from the known vapor pressure and volume of solvent added (100 µL).

Solvent	λ_{emi} (nm)			Intensity (counts)			Percent of Initial Fluorescence	
	**Before**	**During**	**After**	**Before**	**During**	**After**	**During (%)**	**After (%)**
DCM	491	505	487	876.6	533.5	620.8	60.9	70.8
$CHCl_3$	497	516	497	847.5	718.3	705.4	84.8	83.2
Hex	494	477	460	326.9	164.2	139.8	50.2	42.8
THF	496	506	495	804.3	698.2	825.3	86.8	102.6
MeTHF	497	511	496	788.5	789.4	670.6	100.1	85.0
Ether	494	497	478	1001	323.5	204.6	32.3	20.4
MeOH	494	515	494	980.0	590.1	816.5	60.2	83.3
EtOH	494	501	493	758.0	432.3	477.8	57.0	63.0
Tol	494	481	494	943.7	831.8	502.6	88.1	53.3
TEA	494	460	491	864.3	760.2	621.7	88.0	71.9
DEA	493	510	494	792.3	605.4	724.5	76.4	91.4

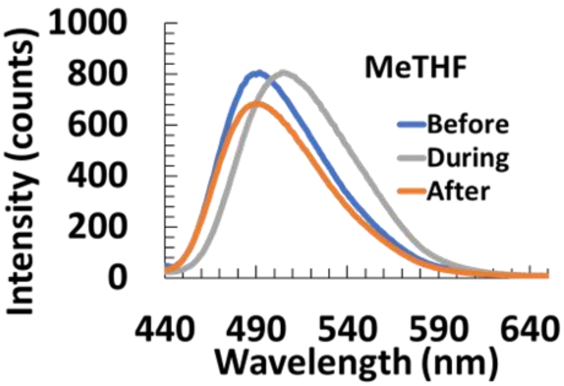

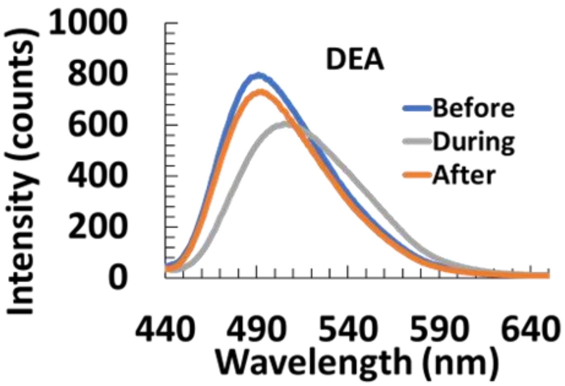

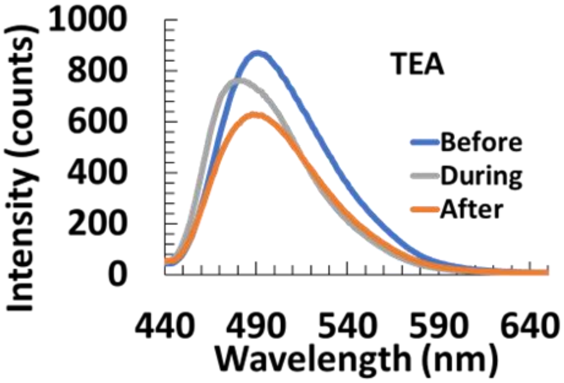

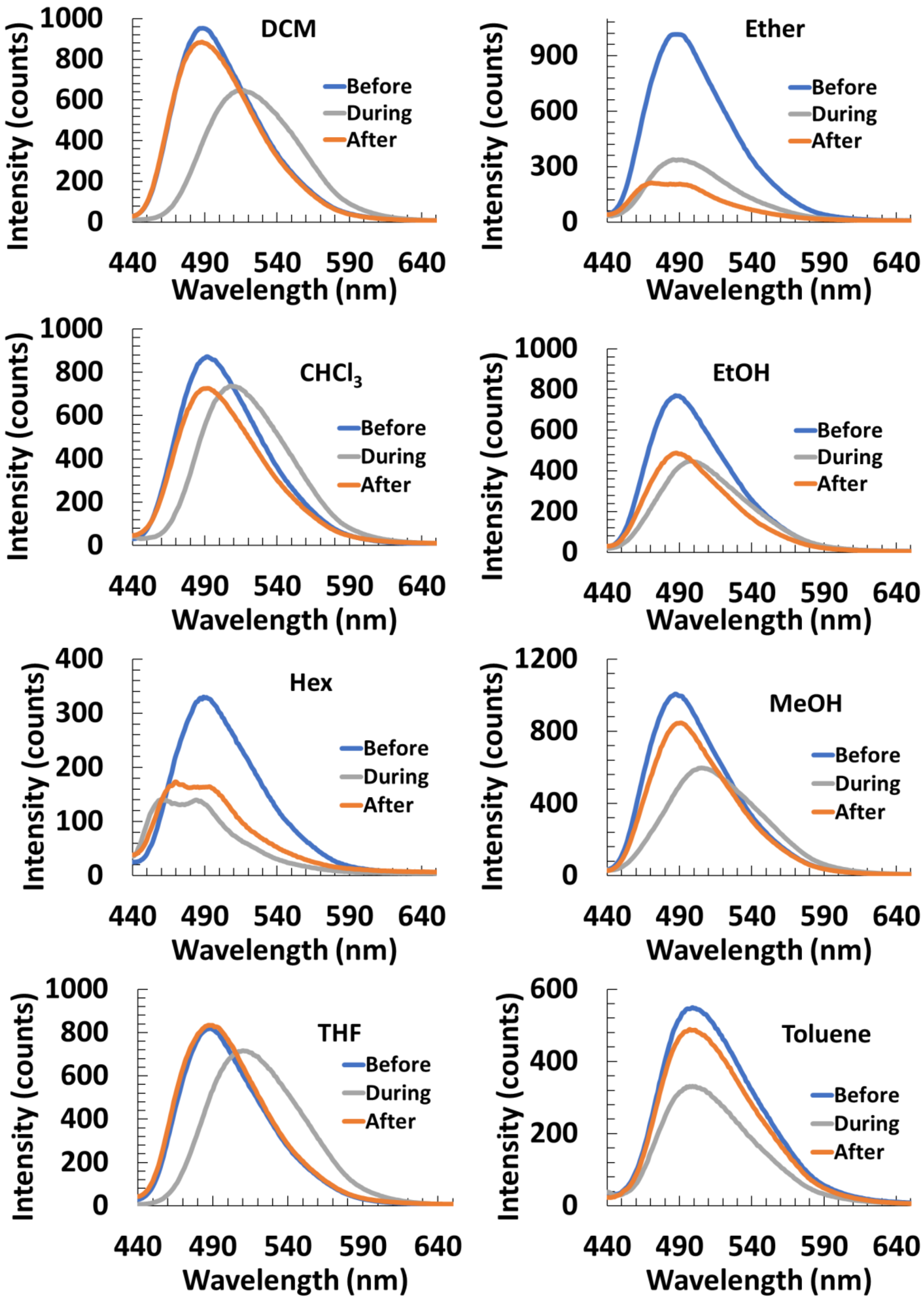

Figure S78: Organic saturated solvent vapor sensing with several solvents using Ph_2N-TTz-Py in SIS.

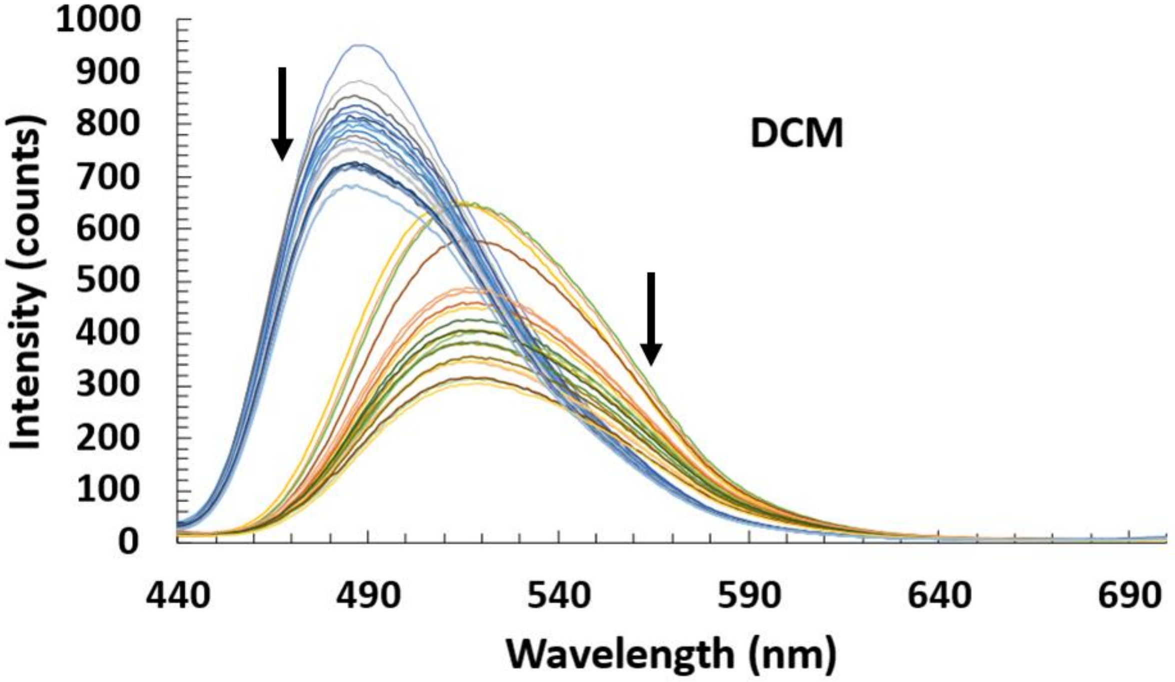

Figure S79: Emission spectra of spin coated Ph_2N-TTz-Py in SIS polymer subsequently exposed to saturated DCM vapors 20 times. (λ_{exi} = 400 nm)

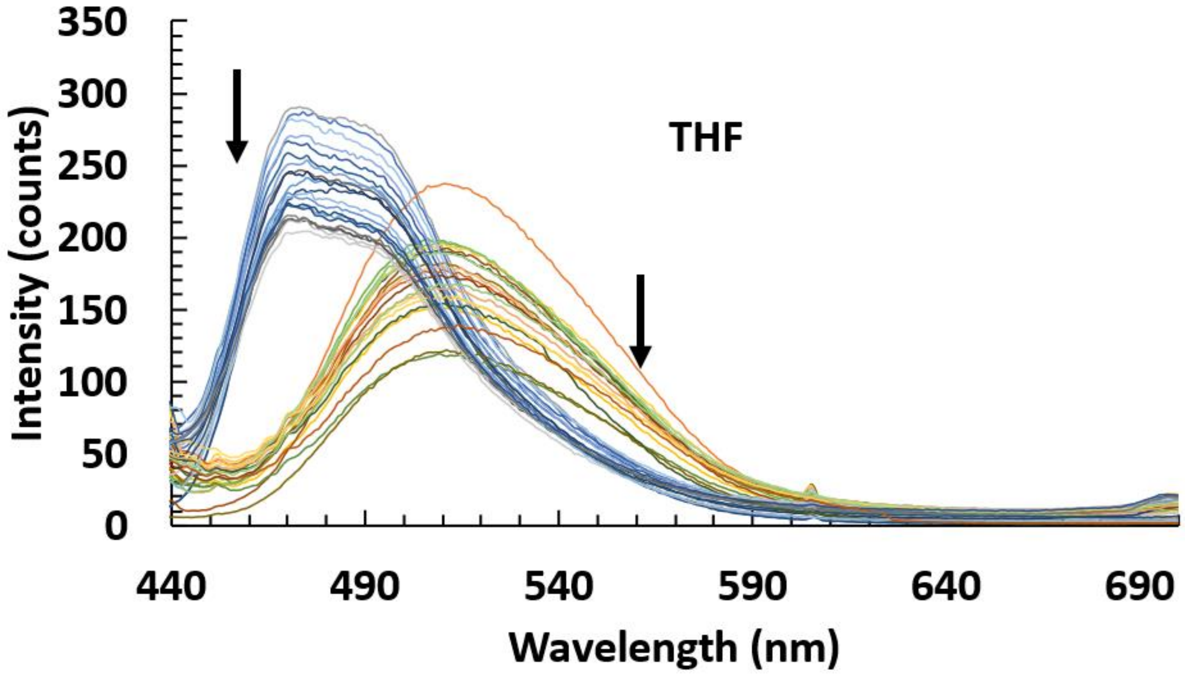

Figure S80: Emission spectra of spin coated Ph_2N-TTz-Py in SIS polymer subsequently exposed to saturated THF vapors 20 times. (λ_{exi} = 400 nm)

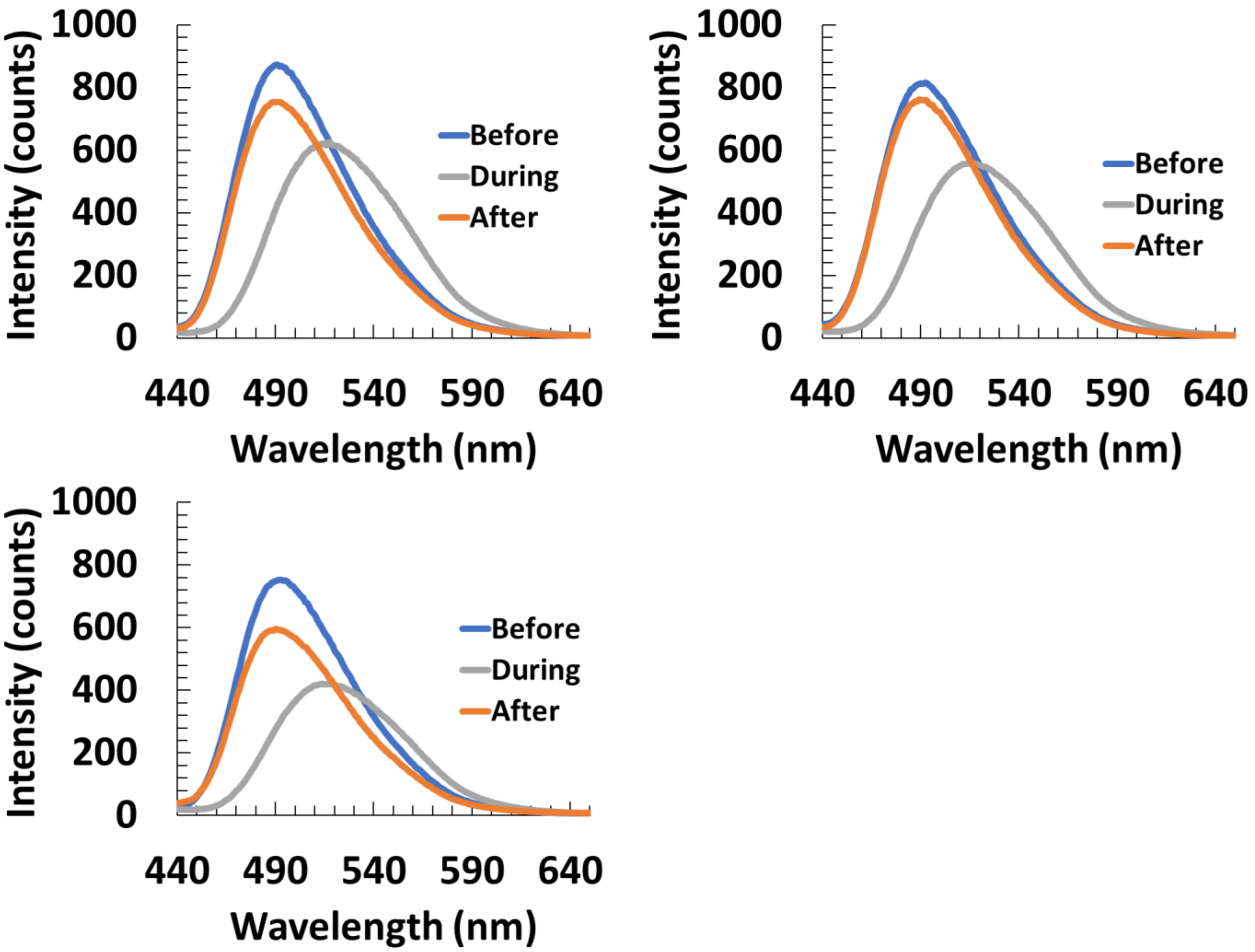

Figure S81: Three trials of 100% sat. vapor sensing of DCM with Ph_2N-TTz-Py in SIS.

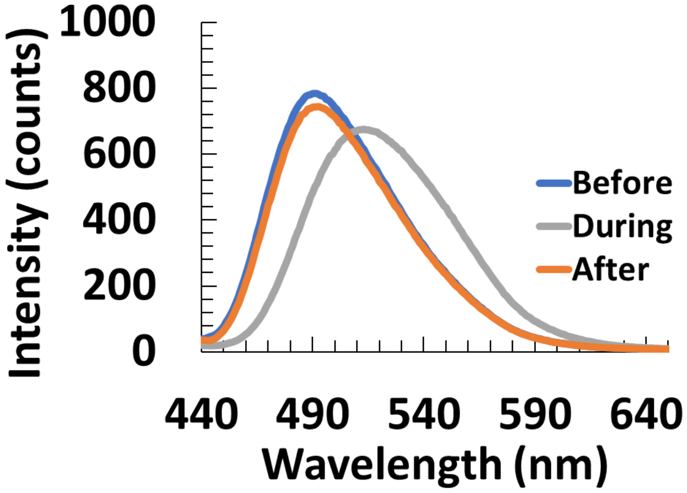

Figure S83: 100% sat. vapor sensing of THF with Ph_2N-TTz-Py in SIS.

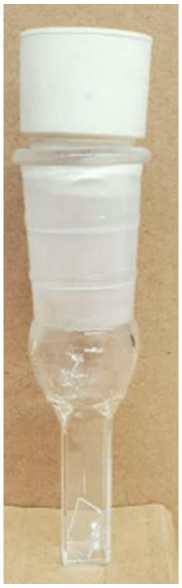

Figure S84: Glass cuvette used to obtain spectra of polymer thin cast films. This cuvette was placed in a 4 L Erlenmeyer and sealed when doing low limit detection

Section 7: Solid-State Characterization

Section 7.1: Lifetimes

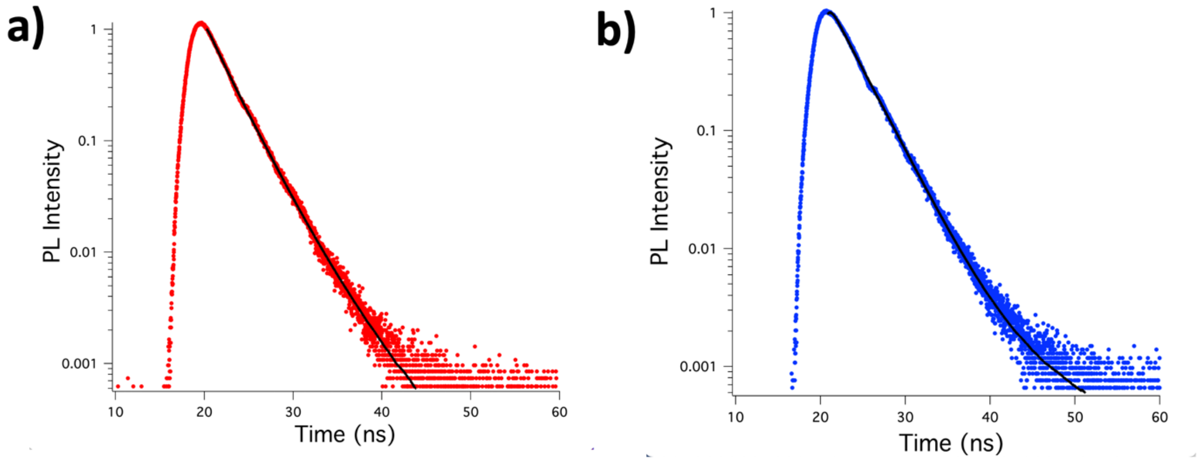

Figure S85: Fluorescence lifetime of a) Ph_2N-TTz-NO_2 (2.64 ns) and b) Ph_2N-TTz-Py (3.18 ns) embedded in SIS polymer.

Section 7.2: Absolute Solid State Fluorescence Quantum Yield

Samples were drop cast on precleaned silicon substrates and illuminated in an integrating sphere using a 400 nm, 10 mW laser to collect fluorescence quantum yield in the solid state. Light intensities scattered within the sphere were measured using Ocean optics spectrometer (QE65000). Fluorescence quantum yields were calculated using the equation:

$$\Phi PL = \frac{\alpha \int \frac{\lambda}{hc} [I' em(\lambda) - Iem\ (\lambda)] d\lambda}{\alpha \int \frac{\lambda}{hc} [Iex(\lambda) - I' ex\ (\lambda)] d\lambda}$$

where α is the calibration factor for the instrument, λ is the wavelength, h is Plank's constant and c is the speed of light. Laser excitation intensity by blank silicon substrate is denoted by *Iex(λ)* and excitation intensity due to drop cast organic samples is denoted by *I'ex(λ)*. The difference in excitation intensity represents the photons absorbed by the organic samples. Light scattering by blank silicon substrate in wavelength range 425 nm to 790 nm is denoted by *Iem(λ)* while *I'em(λ)* represents emission of organic samples. Tris(8-quinolinolato)aluminum(III) complex (Alq_3) was used as a reference to test the accuracy of the method. Fluorescence quantum yield was measured to be 19$\pm$0.2 % which is in strong agreement with previously reported values.[8, 9]

Section 7.3: Compact aTTz Film Solvent Vaper Sensing

Solid-state compact film fluorescence and solvent vapor sensing were conducted by drop casting a saturated solution of Ph_2N-TTz-Py or Ph_2N-TTz-NO_2 on a precleaned microscope slide to form a crystalline film. Slides were exposed to DCM solvent vapors (7 ppm) for 2 min before acquiring fluorescence spectra. Slides were dried on a hot plate (70 °C, 2 min) while solvent chamber was dried using a stream of nitrogen to recover the pre-solvent vapor exposure emission of the films.

Section 8: Crystal Structure Data and Refinement

Table S9. Crystal data and structure refinement for Ph_2N-TTz-Py and Ph_2N-TTz-NO_2 compounds

Sample	**Ph_2N-TTz-PY**	**Ph_2N-TTz-NO_2**
CDCC	2164084	2164083
Empirical formula	$C_{27}H_{18}N_4S_2$	$C_{28}H_{18}N_4O_2S_2$
Formula weight	462.57	506.58
Temperature/K	102(2)	100.4(6)
Crystal system	triclinic	triclinic
Space group	P-1	P-1
a/Å	5.9295(3)	5.5978(3)
b/Å	7.7087(3)	7.9938(3)
c/Å	23.9454(10)	25.8404(10)
α/°	82.790(3)	85.633(3)
β/°	87.429(3)	86.121(4)
γ/°	89.968(3)	89.938(4)
Volume/Å^3	1084.75(8)	1150.29(8)
Z	2	2
ρ_{calc}g/cm^3	1.416	1.463
μ/mm^{-1}	2.410	2.394
F(000)	480.0	524.0
Crystal size/mm^3	0.122 × 0.116 × 0.039	0.475 × 0.306 × 0.053
Radiation	Cu Kα (λ = 1.54184)	Cu Kα (λ = 1.54184)
2Θ range for data collection/°	7.46 to 133.58	10.32 to 133.52
Index ranges	$-7 \leq h \leq 7$<br>$-8 \leq k \leq 9$<br>$-28 \leq l \leq 28$	$-6 \leq h \leq 6$<br>$-9 \leq k \leq 9$<br>$-30 \leq l \leq 30$
Reflections collected	15476	19480
Independent reflections	3814<br>[R_{int} = 0.0381, R_{sigma} = 0.0301]	4064<br>[R_{int} = 0.0575, R_{sigma} = 0.0341]
Data/restraints/parameters	3814/0/334	4064/0/344
Goodness-of-fit on F^2	1.021	1.103
Final R indexes [I>=2σ (I)]	R_1 = 0.0344, wR_2 = 0.0886	R_1 = 0.0516, wR_2 = 0.1306
Final R indexes [all data]	= 0.0431, wR_2 = 0.0961	= 0.0606, wR_2 = 0.1375
Largest diff. peak/hole / e Å^{-3}	0.18/-0.21	0.61/-0.36

Experimental

X-ray crystallography data were acquired with an Agilent (now Rigaku) Gemini A Ultra diffractometer. Crystals of Ph_2N-TTz-Py were grown by vapor diffusion of pentane (non-solvent) into a nearly saturated solution in chloroform. Crystals of Ph_2N-TTz-NO_2 were grown from a chloroform solution by slow evaporation. Crystals of suitable size were coated with a thin layer of paratone-N oil, mounted on the diffractometer, and flash cooled to 100 K in the cold stream of the Cryojet XL liquid

nitrogen cooling device (Oxford Instruments) attached to the diffractometer. The diffractometer was equipped with sealed-tube long fine focus X-ray sources with Mo target (λ = 0.71073 Å) and Cu target (λ = 1.5418 Å), four-circle kappa goniometer, and CCD detector. The Cu target was used for both crystal structures. CrysAlisPro[9] software was used to control the diffractometer and perform data reduction. The crystal structure was solved with SHELXS.[10] All non-hydrogen atoms appeared in the E-map of the correct solution. Alternate cycles of model-building in Olex2[11] and refinement in SHELXL[10] followed. All non-hydrogen atoms were refined anisotropically. All hydrogen atom positions were calculated based on idealized geometry and recalculated after each cycle of least squares. During refinement, hydrogen atom – parent atom vectors were held fixed (riding motion constraint).

Disorder in the TTZ moiety of Ph_2N-TTz-Py was modeled using two orientations of TTZ models each fixed at 50% occupancy (only the heteroatoms were disordered – N and S, the carbon atoms of the TTZ moiety were not split). Likewise, disorder within the TTZ moiety of Ph_2N-TTz-NO_2 was modeled with two orientations of the TTZ moiety (partial occupancies of the two orientations were 0.8848 and 0.1152). The anisotropic displacement parameters of the disordered nitrogen atoms of Ph_2N-TTz-NO_2 were kept equivalent using the EADP constraint.

References.

1. Brouwer, A. M., Standards for photoluminescence quantum yield measurements in solution (IUPAC Technical Report). *Pure and Applied Chemistry* **2011,** *83* (12), 2213-2228.
2. Olorunyomi, J. F.; Sadiq, M. M.; Batten, M.; Konstas, K.; Chen, D.; Doherty, C. M.; Caruso, R. A., Advancing Metal-Organic Frameworks toward Smart Sensing: Enhanced Fluorescence by a Photonic Metal-Organic Framework for Organic Vapor Sensing. *Adv. Opt. Mater.* **2020,** *8* (19), 2000961.
3. Pandeeswari, R.; Jeyaprakash, B. G., High sensing response of β-Ga2O3 thin film towards ammonia vapours: Influencing factors at room temperature. *Sens. Act., B. Chem.* **2014,** *195*, 206-214.
4. Adamo, C.; Barone, V., Toward reliable density functional methods without adjustable parameters: The PBE0 model. *Journal of Chemical Physics* **1999,** *110* (13), 6158-6170.
5. Ditchfield, R.; Hehre, W. J.; Pople, J. A., SELF-CONSISTENT MOLECULAR-ORBITAL METHODS .9. EXTENDED GAUSSIAN-TYPE BASIS FOR MOLECULAR-ORBITAL STUDIES OF ORGANIC MOLECULES. *J. Chem. Phys.* **1971,** *54* (2), 724-+.
6. Seo, E. T.; Nelson, R. F.; Fritsch, J. M.; Marcoux, L. S.; Leedy, D. W.; Adams, R. N., ANODIC OXIDATION PATHWAYS OF AROMATIC AMINES . ELECTROCHEMICAL AND ELECTRON PARAMAGNETIC RESONANCE STUDIES. *J. Am. Chem. Soc.* **1966,** *88* (15), 3498-&.
7. Pham, T. T. T.; Chitose, Y.; Tam, T. T. T.; Tseng, W. L.; Lin, T. C.; Abe, M., Impact of Five-membered Heterocyclic Rings on Photophysical Properties Including Two-photon Absorption Character. *Chem. Lett* **2021,** *50* (10), 1810-1813.
8. Kawamura, Y.; Sasabe, H.; Adachi, C., Simple accurate system for measuring absolute photoluminescence quantum efficiency in organic solid-state thin films (vol 43, pg 7729, 2004). *Japanese Journal of Applied Physics Part 1-Regular Papers Brief Communications & Review Papers* **2005,** *44* (2), 1160-1160.

9. Kishore, V.; Narasimhan, K. L.; Periasamy, N., On the radiative lifetime, quantum yield and fluorescence decay of Alq in thin films. *Physical Chemistry Chemical Physics* **2003,** *5* (7), 1386-1391.
8. M. Thommes, K. Kaneko, A. V. Neimark, J. P. Olivier, F. Rodriguez-Reinoso, J. Rouquerol and K. S. W. Sing, Pure and Applied Chemistry, 2015, 87, 1051-1069.
9. Rigaku Oxford Diffraction, (2018), CrysAlisPro Software System, version 1.171.38.46, Rigaku Corporation, Oxford, UK.
10. G.M. Sheldrick, Acta Cryst. 2008, A64, 112-122.
11. O. V. Dolomanov, L. J. Bourhis, R. J. Gildea, J. A. K. Howard and H. Puschmann, J. Appl. Cryst. 2009, 42, 339-341.
